# Systematic review of the development and effectiveness of digital health information interventions, compared with usual care, in supporting patient preparation for paediatric hospital care, and the impact on their health outcomes

**DOI:** 10.3389/frhs.2023.1103624

**Published:** 2023-04-06

**Authors:** Marie-Claire Demblon, Colin Bicknell, Lisa Aufegger

**Affiliations:** Department of Surgery and Cancer, Imperial College London, London, United Kingdom

**Keywords:** digital technology, paediatric care, health outcomes, patient preparation, health information

## Abstract

**Background and aim:**

Elective surgery can be overwhelming for children, leading to pre-operative anxiety, which is associated with adverse clinical and behavioural outcomes. Evidence shows that paediatric preparation digital health interventions (DHIs) can contribute to reduced pre-operative anxiety and negative behavioural changes. However, this evidence does not consider their design and development in the context of behavioural science. This systematic review used the Theoretical Domains Framework (TDF) to evaluate the design and development of DHIs used to support children up to 14 years of age and their parents, prepare for hospital procedures, and determine any correlation to health outcomes. It also considered whether any behavioural frameworks and co-production were utilised in their design.

**Methods:**

A search of the MEDLINE, EMBASE, PsycINFO, and HMIC databases was carried out, looking for original, empirical research using digital paediatric preparation technologies to reduce pre-operative anxiety and behavioural changes. Limitations for the period (2000–2022), English language, and age applied.

**Results:**

Seventeen studies were included, sixteen randomised control trials and one before and after evaluation study. The results suggest that paediatric preparation DHIs that score highly against the TDF are (1) associated with improved health outcomes, (2) incorporate the use of co-production and behavioural science in their design, (3) are interactive, and (4) are used at home in advance of the planned procedure.

**Conclusion:**

Paediatric preparation DHIs that are co-produced and designed in the context of behavioural science are associated with reduced pre-operative anxiety and improved health outcomes and may be more cost-effective than other interventions.

**Systematic Review Registration:**

https://www.crd.york.ac.uk/prospero/, identifier: CRD42022274182.

## 1. Introduction

Over 500,000 children undergo elective surgery in the United Kingdom annually, with nearly 80% of these being planned day surgeries where the child is admitted and discharged on the same day (1). Anaesthesia, the surgical process, and the hospital environment can be overwhelming for children and their parents, with both often experiencing fear, stress, and apprehension. These emotions are associated with pre-operative anxiety (2, 3).

Heightened pre-operative anxiety can lead to poor anaesthesia induction, an increased risk of emergence delirium, pain, inconsolable crying, irritation, incoherency, and uncooperativeness (4). These frequently negatively impact the child's short- and long-term post-operative psychological and physiological outcomes and can trigger behavioural changes. These include aggression, sleep disturbances, eating problems, a more painful prolonged recovery (5–7), and longer-term maladaptive behaviours such as fear of healthcare professionals and medical environments, avoidance of treatment, separation anxiety, and persisting negative memories of anaesthesia (8, 9), all of which affect healthcare burden and costs.

Various pharmacological and non-pharmacological interventions have been used to reduce pre-operative anxiety in children and improve post-operative psychological and physiological outcomes. Pharmacological interventions include anti-anxiety and sedative drugs, but these commonly cause adverse effects such as drowsiness and can interfere with anaesthesia medication (10). Non-pharmacological interventions traditionally include routine hospital and procedural preparation information, hospital tours, child life specialists, therapeutic play interventions, music therapy, parental presence, clowns, games, and colouring books (10, 11). While non-pharmacological interventions are popular, they are not all readily available and cost-effective and some, like parental presence, have yielded mixed results (3, 11, 12). In addition, many are used as a distraction rather than a pre-operative preparation intervention.

The use of pre-operative preparation interventions indicates that well-prepared children have reduced pre-operative anxiety and negative responses to surgery or medical procedures (13–16). Pre-operative preparation provides information about the planned procedure, hospital environment, and post-operative care and can encompass information on how to cope with emotions, stress, and anxiety (1). Bray et al. (17) and Fortier et al. (18) found that children wish to receive detailed pre-operative information, but it is frequently received through their parents, hampering their direct access and understanding. In addition, children want information that is engaging, easily accessible, and child-friendly. Digital health interventions (DHIs) such as audio-visual, video games, virtual reality (VR), computer or web-based programs or presentations, educational interactive multi-media applications, and smartphone, tablet, or computer applications (Apps) provide a platform for delivering child-friendly, engaging, and accessible pre-operative preparation information. Evidence (19–23) is growing into their use as pre-operative preparation for children and as an intervention to reduce paediatric pre-operative anxiety. However, this evidence does not consider the design and development of DHIs in the context of behavioural science.

Behavioural science is interested in aspects such as behavioural change, in this case, the design and development of paediatric preparation DHIs and their impact on children's emotional, behavioural, and clinical outcomes. Due to the lack of understanding between the preparation DHIs and behavioural change, this systematic review builds upon this research. It looks specifically at the design and development of paediatric preparation DHIs through the application of the Theoretical Domains Framework (TDF). It applies the 14 domains of the TDF to assess the components of DHIs and examines whether there is a correlation to improved outcomes. The TDF was developed from the synthesis of 33 behaviour change theories into a framework comprising 14 domains and 84 behaviour constructs, founded on the Behaviour Change Wheel (24). The Behaviour Change Wheel connects environmental and psychological factors to interventions, established on the COM-B system (Capability, Opportunity, Motivation, Behaviour) where behaviour is produced when capability, motivation, and opportunity interact (25). In building on the Behaviour Change Wheel, the TDF provides a validated framework, developed by behavioural scientists and implementation researchers, to evaluate behaviour change. It can be used to assess implementation issues, support intervention design, and analyse interventions (26).

### 1.1. Current literature

Children undergoing medical procedures, anaesthesia, and surgery experience significant psychological and physiological reactions. The unfamiliar environment, the equipment and routines, fear of separation, needles, and the medical procedure are well documented as sources of these negative reactions (27–29). These reactions lead to short- and long-term maladaptive behaviours such as irritation, aggression, incoherency, uncooperativeness, eating problems, and sleep disturbances (4) and fear of healthcare professionals or medical treatment (8, 9). In addition, they are associated with poor anaesthesia induction compliance (IC) (30), emergence delirium (5), increased need for sedation or rescue analgesia (31), and prolonged pain and recovery (5). To address these psychological and physiological reactions, research has been undertaken on the use of pharmacological and non-pharmacological interventions to reduce pre-operative anxiety.

#### 1.1.1. Interventions to manage pre-operative anxiety

Pharmacological interventions include anti-anxiety and sedation medications, such as Midazolam, Fentanyl, Ketamine, and Clonidine. These are used as effective pre-operative anxiolytic and sedation medications in children, which reduce pre-operative nausea and vomiting, enable satisfactory separation from parents and anaesthesia induction, and reduced the need for post-operative analgesics (32–35). However, they are associated with an increased incidence of respiratory depression, drowsiness, agitation, and paradoxical reactions (32–35).

Due to these adverse side-effects, non-pharmacological interventions have increasingly been used to manage pre-operative anxiety. Research on the use of parental presence is mixed. Some papers suggest it has been used to provide reassurance and comfort, eliminate separation anxiety and reduce the need for medications, while other papers suggest it can increase anxiety if parents themselves are anxious (36–39). Distraction techniques such as videos, singing, reading, colouring, playing games, or controlled breathing are often used to reduce anxiety and shift the focus away from the procedure concerned or the pain experienced (40–43). In addition, complementary and alternative therapies and remedies such as music therapy, art therapy, hypnosis, and clowns (33, 37, 44), cognitive behavioural therapy (37), child life specialists (15), and therapeutic play interventions (45, 46) have shown positive impacts on reducing pre-operative anxiety, enabling self-regulation of emotions and behaviours and acting as a support for children and their families. Other non-pharmacological interventions include preparation programmes such as hospital and operating room tours including exposure to medical equipment and staff (37, 47). Many of these non-pharmacological interventions have a low risk of adverse effects and are minimally invasive (37), but not all are readily available and cost-effective, as they can be time-consuming, requiring staffing resources and planning (11, 12).

Within the last 20 years, there has been increased research into the use of digital technologies such as DHIs to manage pre-operative anxiety either through distraction (7, 48, 49) or through preparation (3, 11, 50, 51). These DHIs include audio-visual, computer games or video games, VR, computer or web-based programs or presentations, educational interactive multi-media applications, and smartphone or tablet applications. Their versatility in being able to tailor pre-operative information for different procedures and child ages, as well as incorporate virtual tours of the hospital environment and operating room, provide information on medical equipment and staff, and use exercises, games, or activities to support understanding and emotional self-regulation, have made them increasingly popular pre-operative preparation interventions. Consequently, this also aids in addressing the findings from research into what children and their parents want from pre-operative information, specifically child-centred, easily accessible, engaging, and informative information with coping strategies (17, 18, 21, 52). Various systematic reviews (6, 19, 20, 53) have been undertaken to consider the effectiveness of DHIs in managing pre-operative anxiety and improving health outcomes. These show that DHIs, as distraction and preparation programmes, can have a positive effect on reducing pre-operative anxiety and negative behavioural changes. However, they do not consider the design and development of DHIs. This is specifically in the context of behavioural science, which includes aspects such as behaviour change, which is important in improving healthcare and health outcomes (24). This systematic review aims to address this gap by using the TDF to assess the design and development of preparation DHIs and the impact on children's health outcomes.

#### 1.1.2. Theoretical domains framework

The TDF provides a validated framework, developed to provide a more accessible and usable tool to support improving the implementation of evidence-based practice. By bringing together a range of behaviour theories and key theoretical constructs, a simple and integrated framework is provided to inform and assess intervention design and implementation (54). The TDF originally included 33 theories and 128 key theoretical constructs, which were later simplified into a framework comprising 14 domains and 84 behaviour constructs. The revised TDF has been validated for use in assessing implementation issues, supporting intervention design, and analysing interventions (26).

This study aimed to evaluate the design and development of paediatric preparation DHIs used to support children up to 14 years of age, and their parents, to prepare for hospital procedures, and to understand their impact on their health outcomes. The primary objective was to evaluate the design and development of paediatric preparation DHIs against the TDF and ascertain whether any behavioural frameworks and co-production were used. A secondary objective, and in the context of the previous systematic reviews (6, 19, 20, 53), was to consider, compared with standard care, the extent to which paediatric preparation DHIs influenced the children's emotional and/or behavioural responses, and/or any impact on their clinical status and/or healthcare utilisation. Specifically, this study was interested in whether there was any correlation between the evaluation of the development of paediatric preparation DHIs and the reported results.

## 2. Methods

The study protocol is publicly available under registration number CRD42022274182 on the International Prospective Register of Systematic Reviews (PROSPERO). The inclusion and exclusion criteria (Supplementary Table S1 in Appendix A) were built using the Population, Intervention, Comparison, Outcome, and Study (PICOS) framework, which is a well-established framework for developing research questions and inclusion and exclusion criteria (55, 56). The population for this review constituted children up to 14 years of age, and their parents, without any cognitive impairments, who were prepared for hospital treatment using a paediatric preparation DHI. Studies were excluded if the DHI was solely aimed at parents or healthcare professionals. Children were excluded if they were aged 15 years and above in order to focus the review on the use of DHIs in younger children and early adolescents. The DHIs needed to be educational and focused on preparation for the procedure, providing information about the hospital environment, medical equipment, and healthcare staff roles and responsibilities. The type of digital interventions was broad, including audio-visual, VR, smartphone or tablet or computer applications, computer or video games, and websites or online programs or games. Any non-digitised health interventions, self-management applications, or digital interventions aimed at distraction were excluded. The studies that were included were randomised control trials, non-randomised control trials, and quasi-experimental studies such as before and after evaluations, to ensure the assessment of original, empirical research. The studies also needed to compare the DHI with usual care or be a head-to-head comparison of two DHIs. All other study types were excluded.

### 2.1. Search strategy and data extraction

The OVID databases that were selected were MEDLINE, EMBASE, PsycINFO, and HMIC. A mix of keywords and Medical Subject Headings (MESHs) was used to search for themes. The search was carried out in February 2022 using the complete syntax with truncation for each database as outlined in Appendix B. Limitations were added for the period (2000–2022), English language, and age.

### 2.2. Study selection

**Figure 1:**
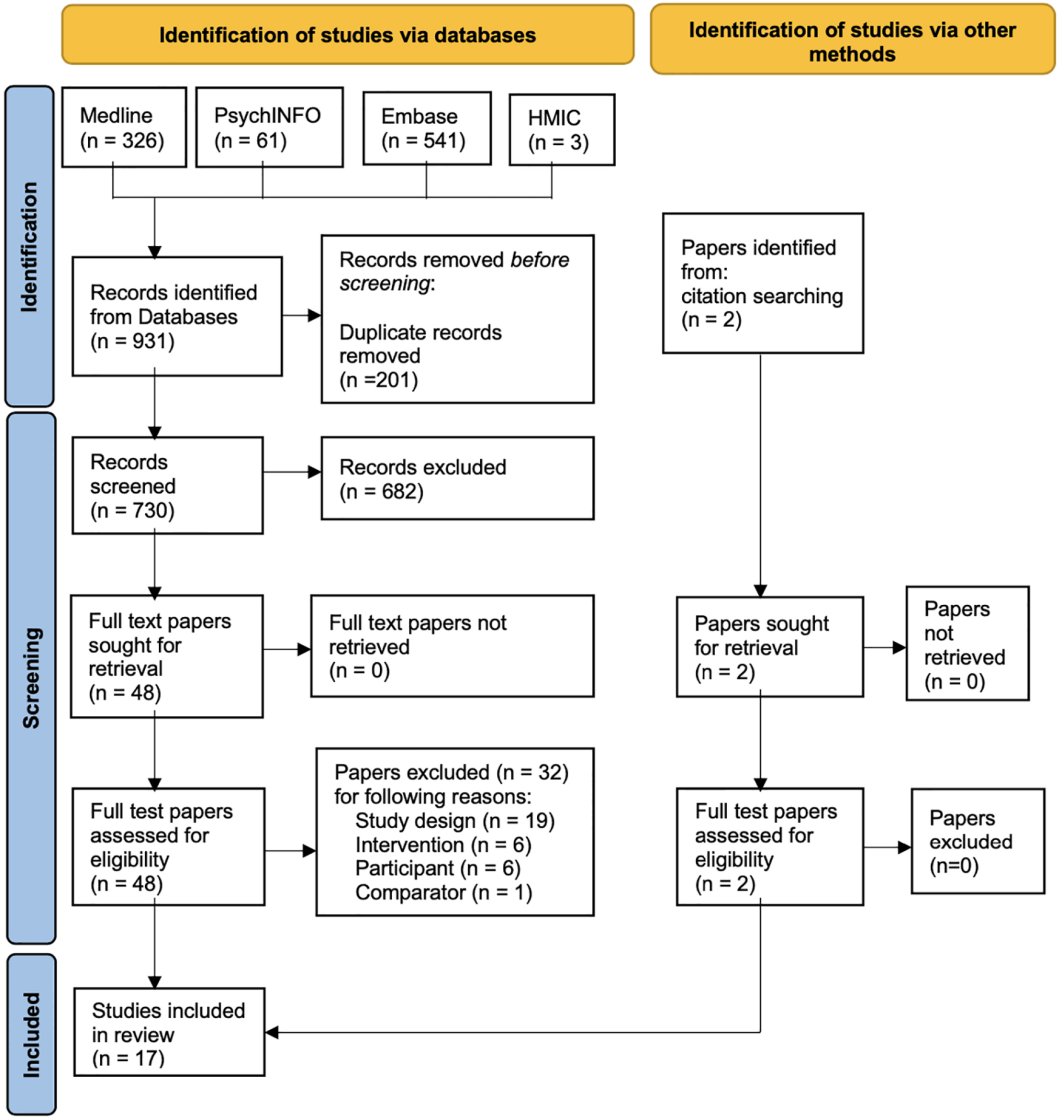
PRISMA flow chart (76) describing records obtained and reasons for exclusion.

The preliminary search returned 931 records; 363 duplicate records were identified, and 176 records were removed. A total of 730 records remained, and these progressed to the stage of title and abstract screening (57). Two reviewers screened titles and abstracts for the 730 records for eligibility against the PICOS, resulting in 655 articles for exclusion, 41 articles for stage full-text screening, and 34 conflicts. After consultation with a third reviewer, 17 (58–74) articles remained for full paper review. The Cohen's Kappa score (75) for the screened title and abstract was 0.682, with a 95% proportionate agreement, and for the full paper review, a score of 0.907 was obtained, with a 96% proportionate agreement, demonstrating substantial agreement among the reviewers. Figure 1 outlines the searching and screening process diagrammatically using the Preferred Reporting Items for Systematic Reviews and Meta-Analysis (PRISMA) (76) flow chart. Appendix C in the Supplementary material shows the full-text screening selection process questions.

Data relevant for extraction were considered against the aims and objectives of the review (77). Supplementary Table S2 in Appendix D sets out the data extraction fields. For any randomised control trials, version 2 of the Cochrane Risk of Bias tool for randomised trials (RoB2) (78) was chosen, given that it is the standard recommended for Cochrane Reviews. For any non-randomised control trials or included quasi-experimental study designs, the Critical Appraisal Skills Programme (CASP) (79) was chosen, given its wide use in systematic assessment of the relevance and results of research.

The synthesis and analysis were first assessed, on the basis of the degree of homogeneity (80, 81), in terms of four aspects: patient characteristics, the intervention and comparators of the studies, the reported outcomes and timeframes over which they were measured, and the similarity of the results. If homogeneity is determined to be insignificant and heterogeneity significant, then a narrative synthesis would be undertaken on the study and participant characteristics, findings from the quality assessment, and the measurements used and reported outcomes. To meet the primary objective of this review, an evaluation of the development (design) of the DHIs was also undertaken. The results were then used to determine any correlation to the evaluation of the DHI development and findings from the studies using a measure of effect. DHI descriptions were evaluated using the information provided within the relevant studies, and where this was insufficient, related articles were sought out. For some studies, no related information was available, and the DHIs were, therefore, assessed using only the information provided in the included paper.

The digital health interventions in the studies are aimed at changing behaviour to reduce pre-operative anxiety through education, information, and coping strategies. The TDF was chosen to evaluate the design and development of the digital health interventions within the context of behavioural science, as it is a validated tool for assessing implementation issues, supporting intervention design, and analysing interventions (24, 26, 82). The DHI evaluation was undertaken using a scoring system against a 16-domain framework. The 16 domains constituted the 14 domains from the TDF (24) and two additional domains. The definitions of the 14 domains from the TDF were adapted from Cane et al. (24) and Smalley et al. (82) with two additional domains added. The additional domains identified as relevant in assessing the development of the DHIs, and added to create a modified TDF, were

1. input from one or more healthcare professionals, children, and parents, and
2. use of any behavioural frameworks.

During pilot testing of the modified TDF against a few studies, it was decided that the TDF’s “social/ professional role and identity” domain was not applicable. This was attributed to its focus on the behaviours and displayed personal qualities in a social or work setting, whereas the TDF domains were being used to assess the design of digital intervention in respect of use by children and their parents. It was subsequently removed and the scoring for the evaluation of the DHIs was adjusted to be out of 15 domains.

For each domain in the modified TDF, the domain descriptions were used to develop a criteria checklist to guide the evaluation of the DHIs. The criteria checklist considered what information, activities, techniques, or actions the DHIs should incorporate to meet the domain descriptions. This was tested against a sample of the DHIs to refine the criteria checklist. Each DHI was then assessed against each domain criteria checklist and a score applied depending on whether the DHI fully met, partially met, or did not meet the requirements in the criteria checklist. Table 1 sets out the criteria checklist used to evaluate the DHIs against the modified 15-domain TDF. The scoring system applied to the 13 domains from the TDF was “1” if the DHI fully met the criteria, “0.5” if the DHI partially met the criteria, and “0” if either the DHI did not meet the criteria or insufficient information was provided. The scoring for the co-production domain (described in Table 1 as “input into the development of DHI”) was “1” if the paper evidenced development involved healthcare professionals, children, and parents, “0.5” if the paper evidence development only involved one or two of these groups, and “0” if the paper did not evidence involvement from these groups. The scoring applied to the domain for use of behavioural and/or design frameworks in DHI development was “1” if the paper explicitly evidenced their use and “0” if the paper did not evidence their use. The scores were summed to provide an overall score out of 15 for each of the DHIs in the included studies, with those scoring higher assumed to have optimal design and development through meeting more of the modified TDF domains. The scores were also summed to provide totals on how many of the DHIs scored fully (given a score of 1) or partially (given a score of 0.5) against each domain. These scores were then used to determine any correlation between the DHI designs and health outcomes.

**Table 1:** Theoretical and additional domains of the modified TDF demonstrated in the digital health interventions.

Domain	Explanation adapted from Cane et al. (24) and Smalley et al. (82)	Criteria for DHI to meet fully or partially meet the domain.
Knowledge	Awareness of the existence of something, including a knowledge of the condition and the procedure, and what will happen	DHI provides detailed information about the hospital environment, the equipment (e.g., monitoring devices, pulse oximeter, anaesthetic mask, etc.), and the staff. It guides the user through the process from admission to the operating room. Domain is partially met if information lacks detail.
Skills	Ability or proficiency acquired through practice (e.g., skills, ability, competence, practice)	DHI includes an element that is interactive and aimed at developing an understanding of the pre-operative process and/or coping skills (e.g., modelling or breathing). Domain is partially met if not interactive but includes information or support on coping or post-operative care.
Emotion	A pattern of experiential, behavioural, and physiological reactions to deal with significant events or matters (e.g., anxiety, fear, stress)	DHI includes information about emotions, how the child might feel, how to cope with being anxious or scared, and the likely sensory aspects. Domain is partially met whether about coping with anxiety or some consideration of feelings.
Behavioural Regulation	Supports or activities aimed at managing or changing objectively observed actions (e.g., action planning, self-monitoring, breaking habits)	DHI includes activities or techniques aimed at changing behaviours, whether there are coping strategies, behaviour training, or breathing. Domain is partially met if modelling, with no activities or techniques.
Memory, Attention, and Decision Processes	Ability to retain information and selectively focus and choose among options (e.g., decision making, attention, and attention span)	DHI is interactive and may include prompts or challenges. Domain is partially met if DHI noted as taking account of children’s memory and cognition but is not interactive. Also, partially met if DHI is short and provides information about how it is engaging.
Environmental Context and Resources	Circumstances of the environment that contribute (positively or negatively) to skill development, independence, and adaptive behaviour (e.g., organisational culture, resources, and environmental stressors)	DHI includes information on the hospital environment, staff, and equipment. Domain is partially met if all the information listed above is not provided.
Beliefs about Capabilities	Acceptance of one's true abilities, talents, or facilities (e.g., self-confidence, self-esteem, empowerment, self-efficacy, and perceived behavioural control)	DHI includes information or activities to help the child cope or manage behaviour or provides challenges. No partial scoring for this domain.
Beliefs about Consequences	Acceptance of true outcomes of behaviour in each situation (e.g., anticipated regrets and outcomes, beliefs, and consequences of actions)	DHI incorporates one or more of the following: (1) a step-by-step guide of what will happen and is involved, (2) what the outcome will be through information on the experience and how it might feel, and (3) using level progression or interactive games to check the level of understanding. Essentially, it provides sufficient information to create a level of understanding about the consequences of what will happen. Domain is partially met if the guide on what will happen is not a step-by-step one and does not include any other elements listed above.
Reinforcement	The increasing likelihood of desired behaviour by creating a stimulus and response dependency (e.g., incentives, rewards, punishments)	DHI is interactive or includes game elements to reinforce information. Domain partially met if the DHI can be used more than once.
Intentions	Consciously act in a certain way, or perform a certain behaviour	DHI includes feedback or rewards to drive action or behaviours or incorporates specific behavioural components. Domain is partially met if it includes exercises.
Goals	Outcomes or end states that an individual wants to achieve (e.g., setting a target, priorities, and action planning)	DHI requires specific action to progress levels, incorporates setting goals, and includes rewards. Domain is partially met if it includes actions to perform to achieve something specific.
Social influences	Interpersonal processes that can cause individuals to change their thoughts, feelings, or behaviours (e.g., social pressure, norms and support, group identity, and power)	DHI includes a parent element or considers familial influences on the child. Domain is partially met if it uses only famous characters or only partially considers familial influences.
Optimism	Confidence that desired goals will be attained (e.g., optimism, pessimism, identity)	DHI includes some form of reward or attainment. Domain is partially met if reward or attainment is indicated but not sufficiently detailed.
Input into the development of DHI	Does the DHI involve healthcare professionals, parents, and children in its development?	DHI is developed with the involvement of healthcare professionals, parents, and children. Domain is partially met if only one or two of these groups are involved in the development of the DHI.
Behaviour framework	Does the development of the DHI involve the use of any behavioural and/or design frameworks?	DHI is developed using a behaviour framework or tools or concepts. It considers the user and/or behaviour change. No partial scoring for this domain.

DHI, digital health intervention.

To determine any correlation between the evaluation of the development of the DHIs and the reported outcomes, quantitative data was converted into a summary statistic. Specifically, this examined what outcomes were measured and how, whether there was a noticeable measure of effect, and how it correlated to the scoring from the DHI evaluation. To ensure that the data analysis met the requirement of systematic review transparency, established reporting guidelines were followed (83).

The effect size measure and direction was calculated where feasible using a standardised mean difference, Cohen's *d*, Glass's delta, and Hedges’ *g* (84), or other appropriate statistical calculations such as a Chi-square *p*-value calculation (85). Where data are presented in studies using median and interquartile range (IQR) and where there is no evidence of significantly skewed data, median and IQR was converted to an estimated mean and standard deviation (SD), using an online calculator (86) developed from research by Wan et al. (87), Lou et al. (88), and Shi et al. (89, 90). Where estimate mean and SD can be derived, the results were used to calculate the effect size. Similarly, where the mean is provided but not SD, SD was calculated using the RevMan Calculator (91), with the subsequent effect size also calculated. Table 2 outlines the scoring criteria to determine the direction of the effect.

**Table 2:** Scoring criteria to determine the statistically significant direction of effect.

Effect size interpretation (Cohen's *d*, Glass's delta, or Hedges’ *g*) rounded to two decimal places	Direction of effect	
	Positive	Negative
No overall effect (no significance) < 0.20	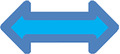	
Small = 0.20 to <0.50	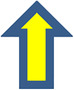	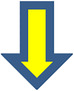
Medium = 0.50 to <0.80	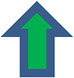	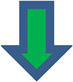
Large = 0.80 or more	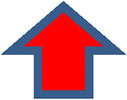	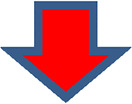

## 3. Results

A total of 17 studies were included in this review, of which 16 were prospective randomised controlled trials (59–74) and one was a before and after evaluation study (58). Of the randomised controlled trials, five were triple-arm parallel randomised control trials comparing the DHI with a control and comparator and one a Solomon four-group design. The rest were all two-arm parallel randomised control trials. The studies were carried out between 2002 and 2020. The publication dates ranged between 2015 and 2021 for 15 studies, with two published before this in 2005 and 2009. Supplementary Tables S5, S6 in Appendix E summarise the study characteristics, DHIs, and participant characteristics.

Homogeneity was observed in parts of the 17 studies. However, when examining the four key aspects that Brown and Richardson (81) consider are required to determine homogeneity, the overall assessment was that there was significant heterogeneity. This was notable in respect of participant age.

### 3.1. Study characteristics and DHIs

The studies were mostly conducted in developed countries, with three in the United Kingdom (58, 65, 72), two in Canada (59, 62), four in South Korea (60, 61, 63, 70), and one each in the United States (71), Thailand (64), Portugal (67), Turkey (66), the Netherlands (68), Italy (69), and Japan (73). The study by Dehghan et al. (74) was conducted in Iran. Study durations varied, with six studies being conducted over 8 months or less, eight being between 10 and 18 months, two at 20 and 23 months, respectively, and one not stating the duration. All DHIs were utilised pre-operatively. The length of the DHIs ranged from 344 s (66) to a maximum of 45 min (59), with four studies (58, 64, 65, 72) not stating the length and the rest being between 4 and 15 min.

The DHIs trialled in the studies are divided into four main types—VR (59–61, 63, 68, 70, 74), audio-visual presentations (64, 66, 69, 73), web-based programs or presentations (62, 65, 71, 72), and educational interactive multi-media applications (58, 67). All DHIs incorporated a tour or information, in varying levels of detail, about the hospital environment and equipment, but only 11 studies (58–61, 63, 66–68, 70, 71, 73) explicitly stated that the information included details of the staff involved. Of the seven studies using a VR-based DHI (59–61, 63, 68, 70, 74), Stunden et al. (59), Eijlers et al. (68), and Ryu et al. (70) incorporated interactive elements, with the rest being informational video tours. The DHIs by Bray et al. (58), Wright et al. (62), Wantanakorn et al. (64), Fernandes et al. (67), and Fortier et al. (71) also incorporated interactive elements such as games and chatbots.

Except for five studies (63, 67, 68, 73, 74), all other studies used usual care in the control group, and this comprised standard verbal information and/or information leaflets. Of those studies using usual care, four were three-arm parallel randomised control trials and involved a comparator, and these were a Child Life Program (CLP) (59), handwashing game (65), voice recording (66), and cartoon strip (72). Park et al. (63) used the same video tour for the control group but without the mirror display for parents to watch simultaneously as their child as used for the intervention. Fernandes et al. (67) used a video game as a comparator and no intervention as the control. Eijlers et al. (68) and Wakimizu et al. (73) used audio-visual tour/information as the control, with the latter being the same as for the DHI intervention group but only viewed once a week in advance of the procedure. Dehghan et al. (74) used parental presence as the control.

The setting for the studies was linked to where the intervention DHIs were used. The majority were used once in the hospital either on the day before the procedure (64, 69) or on the same day as the procedure (59–61, 63, 67, 68, 70, 72), with four of the same-day DHIs being one hour pre-operatively. Hatipoglu et al. (66) presented the DHI once, 1 week in advance of the procedure during hospital admission. The DHIs for the rest of the studies were used either at home (58, 62, 71) or both at home and in the hospital (65, 73), but for all five of these studies, the DHIs could be accessed by children and parents more than once. For the studies where the DHIs could be used at home, one (71) was made available a week before and up to 7 days after the procedure, three (62, 65, 73) were made available a week before the procedure, and one (58) 3 days before the procedure. It is unclear in the Dehghan et al. (74) study when the DHI was used relevant to the procedure, but it is assumed that the setting was in hospital post the randomisation of participants.

### 3.2. Participants

The total sample size across the 17 studies was 1,726 children, with sample sizes ranging between 40 and 200. The ages of the children ranged between 2 and 14 years, with three studies (65, 71, 73) including only younger children between the ages of 2 and 7 years. The reporting of sex across the studies was not consistent, with seven studies (59–62, 65, 68, 70) reporting the sex breakdown of only those included in the analysis and the rest reporting the sex breakdown of the children randomised. In total, of the sex breakdown reported, there were 980 males and 718 females. The only studies to report on child ethnicity were Wright et al. (62) and Fortier et al. (71). Eight studies (58, 59, 62, 64, 66, 67, 69, 73) included baseline information on the number of previous surgeries and/or hospitalisations by the children.

Inclusion criteria across all 17 studies were children within the studies specified age range, undergoing the relevant included procedures and without any cognitive impairments. Children were explicitly excluded from 11 studies (59–64, 67, 68, 70, 71, 73) with visual and/or developmental and/or auditory delays. Language was an exclusion in eight studies, with Stunden et al. (59), Wright et al. (62), Fortier et al. (71), and Campbell et al. (72) limited to English, Fernandes et al. (67) limited to Portuguese, Eijlers et al. (68) limited to Dutch, Liguori et al. (69) limited to Italian, and Wakimizu et al. (73) limited to Japanese. A history of seizures or epilepsy was an exclusion criterion in six (59–61, 63, 68, 70) of the seven VR DHIs, with Dehghan et al. (74) stating the only exclusion as “stress or special problems in using eyeglass or headphone in [virtual reality exposure therapy]” (p. 3).

Parents were included in 10 studies (58, 59, 62, 63, 65, 67, 68, 70, 71, 73). Six studies (58, 65–68, 71) reported baseline information on the educational socioeconomic status of the child's parents. Fortier et al. (71) also included information on parental income. Parental age was reported in seven studies (62, 65–67, 69, 71, 73) and parental ethnicity was reported only by Wright et al. (62).

The procedures that the children were undergoing across the studies were mostly for surgery, including elective and ambulatory surgery (60–63, 66–71, 74). The types of surgery differed across the studies, but the most noted were otolaryngology; ophthalmic; orthopaedic; dental; ear, nose, and throat (ENT); urology; herniorrhaphy; and tonsillectomy. The other procedures included tooth extractions (65, 72), magnetic resonance imaging (MRI) (59), and bone marrow aspirations (64). Bray et al. (58) included children undergoing both invasive (surgery, cannulation, and blood tests) and non-invasive procedures (x-ray or ultrasound). Wakimizu et al. (73) included only children undergoing herniorrhaphy.

### 3.3. Assessment of DHI development

There were 15 unique DHIs across the 17 included studies, with the same DHI used in three (60, 61, 63). The DHIs were scored against the 15 domains in the modified TDF, where 1, 0.5, or 0, respectively, meant that it either fully, partially, or did not demonstrate the domain. Supplementary Table S7 in Appendix F provides the results of the DHI assessment against the 15 domains in the modified TDF, while Table 3 offers a commentary for each.

**Table 3:** Further details on the m-TDF assessment.

Included papers Domain	Bray et al. (58)	Stunden et al. (59)	Ryu et al. (60, 61), Park et al. (63)	Wright et al. (62)	Wantanakorn et al. (64)	Huntington et al. (65)	Fernandes et al. (67)
Knowledge	The platform provides information about the procedure, hospital environment including wards and operating theatres, the key healthcare staff involved, and the hospital equipment (p. 3). It uses a customisable avatar as a guide and chatbot.	VR movie and App provides information about the MRI procedure, the hospital equipment, and the staff involved (p. 3). It starts by introducing a radiologist and a peer in the reception area and then leads the user through an interactive guided tour of the hospital reception area, imaging room, and the steps of a head scan.	VR movie provides information about the procedure, hospital environment including the wards and equipment, and the staff (p. 99 and 1629, respectively). It takes the viewer through the process from admission to the operating theatre, and Pororo explains the process throughout in detail. It starts with Pororo changing into a hospital gown, having an IV catheter placed in his forearm, and then going into the operating room.	I-PPP includes two modules on education and information about the day surgery process and anaesthesia and anaesthesia protocol. Delivered *via* an interactive, virtual tour of the hospital including the admission area, day surgery room, holding area, operating room, and activities that take place in those locations. For anaesthesia, it covers what it is, the types, the purpose, and the process (p. 628).	Short, animated video in Mobile App provides patient information about the procedure including equipment used, and what to expect through the whole process from positioning, local anaesthesia, and sedation to the post-recovery process (p. 644).	Online web-based information over several screens/slides setting out a story of a 6-year-old child called Scott going through the process of the procedure, with child and parents “mouse clicking” through (From p. 159 plus additional paper Reynolds et al. (2012).	The App provides information on (1) hospital admission; (2) healthcare staff and hospital rules; (3) medical instruments; (4) medical procedures; (5) surgery room; (6) recovery room; and (7) aftercare and going home (p. 1192).
Skills	The platform uses a Q&A chatbot and games to create an understanding of the procedure and enable interactive engagement (p. 3).	The App includes elements to help the user learn how to interact with the virtual environment and cues to activate the next steps (p. 3).	The movie is not interactive only informative (p. 99 and 1629, respectively).	I-PPP includes a module on skills development through instructions regarding the continued practice of shaping and exposure to an anaesthetic mask (p. 628).	The game element is interactive, with breathing skills developed through the relevant game, but the movie element is purely informational.	Partially. Includes two videos that model appropriate coping behaviour and teach coping skills (p. 159).	The application uses interactive game activities after each level to guarantee that the information provided is understood by the child (p. 1192)
Emotion	Provides information on the sensory aspects of the procedure, and what the child may experience or feel to help reduce fear and anxiety (p. 3).	Through the inclusion of familiar sounds using narratives to support the child to cope with the loud and noisy MRI sound, to provide comfort, and thus to reduce stress.	Unclear if the movie addresses emotion. Reference made to Pororo emphasising that children will undergo the same process without difficulty, but there is no reference to considering anything that addresses dealing with fear, anxiety, etc. (p. 99 and 1629, respectively).	I-PPP includes a module on the identification of emotions and thoughts associated with day surgery experiences such as anxiety and worry (p. 628).	The game section is included in the App to support the child to cope with anxiety (p. 644).	Partially. Includes two videos that model appropriate coping behaviour and teach coping skills (p. 159).	The application uses a facial expression of the game character for the child to choose whether they are sad, happy, angry, or fearful (p. 1192). The application starts with a brief video explaining how to report emotions.
Behavioural Regulation	Provides information on coping strategies to manage behaviour (p. 3).	The App uses “staying still” to progress through the levels in the VR-MRI app, so drives behavioural regulation to achieve stillness (p. 7).	No information is provided.	I-PPP includes a module on behavioural training through shaping and exposure to an anaesthetic mask with a mask provided (p. 628)	The game section included in the App provides breathing exercises and coping skills (p. 644).	Partially. Includes two videos that model appropriate coping behaviour and teach coping skills (p. 159).	No information is provided to judge if the application supports any behavioural regulation.
Memory, Attention, and Decision Processes	Partially. Designed with children to ensure that it is engaging, acceptable, and effective. Also considers ease of navigation (p. 3).	User-led tour of hospital, staff, and equipment, through interactive hotspots designed to activate transition between rooms or sequences and the use of a stalling sequence to deal with any distractions (p. 3 and 4).	Unclear. While the movie was short, being only 4 min long, it is not clear from the papers whether a child's attention and memory were factored into its development.	I-PPP is interactive and is initially provided sequentially, but once completed, the child and parents can return to any module (p. 628).	Unclear. It is not clear from the papers whether a child's attention and memory were factored into its development. No indication that the child could rewatch the video.	Unclear, as insufficient information is provided in the paper or other papers identified and reviewed.	The application uses interactive game activities after each level to guarantee that the information provided is understood by the child (p. 1192).
Environmental Context and Resources	Provides information and images of the hospital environment (wards and operating theatres), equipment, and key staff (p. 3).	Provides information and images of the hospital environment, equipment, and staff. Also includes stimulations of sounds during an MRI (p. 3 and 4).	Provides information and images of the hospital environment, equipment, and staff. Includes scenes of having an intravenous catheter inserted, pressure cuff and pulse oximeters placed, and a facial mask applied for anaesthesia (p. 99 and 1629, respectively).	Module 1 is an interactive, virtual tour of the hospital including the admission area, day surgery room, holding area, operating room, and activities that take place in those locations (p. 628).	Partially. Provides information on the instruments used, and the images suggest some context of the hospital setting (p. 644), but not as detailed as with other applications/ tools, so partially met.	Partially. Appears to provide information on the procedure but is unclear on the level of information provided (p. 159).	The application provides information on healthcare staff and hospital rules; medical instruments; surgery room; recovery room (p. 1192).
Beliefs about Capabilities	Assumed through reference to what the child may experience and building of coping strategies—empowerment, self-confidence (p. 3).	Assumed through levels becoming more challenging to achieve as feedback mechanism is reduced and children are required to continue independently (p. 7).	Unclear. The video narration is noted to emphasise that all children undergo the same process without difficulty, but there are no references to coping, emotions, or feelings (p. 99).	I-PPP includes a module on coping instructions for parents to support their child (p. 628) and behavioural training (p. 628).	Breathing exercises and games help in coping with anxiety, help build self-confidence, and provide empowerment (p. 644).	Unclear as insufficient information is provided in the paper or other papers identified and reviewed.	Unclear as insufficient information is provided in the paper, and there is no indication of building confidence or esteem through coping.
Beliefs about Consequences	Understanding of the procedure and what the outcome will be through information on how it may feel or what may be experienced (p. 3).	Assumed through not progressing the levels because of not staying still (p. 7).	Taking the child through a narrative guided tour explaining the processes and showing what equipment is used (mask, ECG, blood pressure cuff, etc.) are deemed to support building an understanding of the consequences of the processes.	Assumed through a detailed virtual tour of hospital and experience, and behavioural component, specifically shaping and exposure to anaesthesia mask (p. 628) and practice of the skill module.	Assumed through the provision of information on the whole bone marrow aspiration process through a step-by-step guided video (p. 644) showing what happens by using cartoon children and healthcare professionals.	Unclear as insufficient information is provided in the paper or other papers identified and reviewed.	The use of the interactive game to confirm if the child has understood the information provided and an explanation of procedural information including rules.
Reinforcement	Use of interactive games.	Stalling of the sequence until the child's attention is refocussed appropriately (p. 4). Use of stillness as a measure to progress through levels (p. 7).	Simple 4-minute video taking the child through a guided tour from admission to the operating theatre, but there is no use of interactive tools, nor is there any implication whether the child could rewatch the video.	I-PPP is interactive and is initially provided sequentially, but once completed, the child and parents can return to any module (p. 628). Participants are encouraged to use the I-PPP more than once, particularly the behavioural component. Modules on the practice of skills, coping instructions, and emotions reinforce desired behaviour.	Breathing exercises and games to help in coping with anxiety and help reinforce behaviour to reduce anxiety (p. 644).	Unclear as insufficient information is provided in the paper or other papers identified and reviewed.	The application uses interactive game activities after each level to guarantee that the information provided is understood by the child (p. 1192)
Intentions	Not demonstrated in the paper.	Uses interactive real-time feedback on the indicators of movement to support progression through the levels (p. 7).	No, due to the intervention being a video.	Assumed through behavioural components, specifically shaping and exposure to anaesthesia mask (p. 628).	Partially met through breathing exercises to promote specific behaviour.	Unclear as insufficient information is provided in the paper or other papers identified and reviewed.	Does not address this.
Goals	Not demonstrated in the paper.	Uses interactive real-time feedback on the indicators of movement to support progression through the levels (p. 7).	No, due to the intervention being a video.	Not demonstrated.	Not demonstrated.	Unclear as insufficient information is provided in the paper or other papers identified and reviewed.	Unclear as insufficient information is provided in the paper but suspect not.
Social influences	In a supplementary paper (081) covering information on parents being with a child or being supported by something familiar.	Unclear from the paper or methodology used in development.	Partially. The VR game incorporates a famous animated character to socialise the content in a child-friendly manner (p. 3).	Inclusion of a parent and child path in the program that would support discussion with the child (Wright et al. 2020, p. 306 and 307).	Not demonstrated.	Unclear as insufficient information is provided in the paper or other papers identified and reviewed.	Partially met through reference to information on parental separation (p. 1192).
Optimism	Not demonstrated.	Partially. Includes level attainment but unclear from the paper if level attainment is set out in advance for children to create optimism in achieving level 3.	No, due to the intervention being a video.	Not demonstrated.	Not demonstrated.	Not demonstrated.	Not demonstrated.
Co-production with: - healthcare professionals - children - parents	YES. Developed with all three.	PARTIAL: Developed with the research team, various healthcare professionals, and system administrators. No indication of development with the children or parents.	PARTIAL: The script for the movie is developed by an anaesthesiologist from Seoul National University Bundang Hospital, with doctors and nurses acting in the movie.	YES. Developed and tested with all three through separate studies (see Wright et al. 2017).	PARTIAL: The mobile App was tested with healthcare professionals and a small sample of children (p .645). No evidence that parents were involved.	YES: The paper notes that expert consultation and focus groups were used to develop the tool (p. 158 and 163), with this supported by information in Reynolds et al. (2012) and the study protocol.	PARTIAL: The paper notes that a pilot study involved 490 children and healthcare professionals to improve the application (p. 1192). No reference to the involvement of parents in the development of the tool.
Used a behaviour framework or	YES: Used a person-based approach as described by Yardley et al. (2015) [paper 082].	Unclear. Used an agile development methodology but unclear if a behavioural approach was used as part of this.	NO. No reference was provided to ascertain whether any behavioural frameworks were used.	YES. Wright et.al (2017) notes that cognitive behavioural intervention for anxiety disorders in children and behavioural preparation were used in developing the components of the I-PPP (p. 49).	Unclear. Reference made to the development team studying the characteristics and requirements of young children, but not to the use of behavioural frameworks or tools (p. 645).	Unclear as insufficient information is provided in the paper or other papers identified and reviewed.	Yes. The paper references the Social Learning Theory's theoretical framework (p. 1191) and that certain behaviours can be learned and reproduced, with modelling being effective to reinforce self-efficacy.

**Table 4:** 

Included Papers Domain	Hatipoglu et al. (66)	Eijlers et al. (68)	Liguori et al. (69) [Online video (92)]	Ryu et al. (70)	Fortier et al. (71) [Kain et al. (93)]	Campbell et al. (72)	Wakimizu et al. (73)	Dehghan et al. (74)
Knowledge	Video recording that informs about the surgery and anaesthesia methods, what the duties of the anaesthesiologist are, what will happen, and what equipment is used and some of the hospital staff involved in the procedure, and what the operating and recovery rooms look like (p. 794).	The virtual reality storyline shows the child information about the hospital environment, staff, and equipment and what will happen from admission to recovery (p. 730). Different instruments can be explained by the child by pointing towards them creating an interactive explanation.	Partially. The video uses two clowns, Dr Cloud and Dr Wisp, who, funnily and engagingly, show the operating room and explain some of the equipment used (p. 2). It does not provide any information on staff or the wider hospital environment.	Virtual reality storyline with game elements explaining the pre-operative process through a 360-degree, three-dimensional virtual environment in a first-person perspective. It uses famous characters to explain the pre-operative process from putting on the hospital gown to being transported into the operating room and includes information on equipment and staff, how to use the anaesthetic mask, etc. (p. 3).	The intervention provides web-based information across four modules: (1) at home before surgery, (2) holding area and anaesthesia induction, (3) recovery room, and (4) at home after surgery (p. 908). Provides information on the hospital environment, staff, and equipment through videos and games.	The computer package provides information on the process before dental extraction. It includes details of the staff involved by clicking on each of their images and explains that the child is sent to sleep when “magic wind from a space mask” is applied to the face. It provides some information on recovery when Scott wakes up and feels “fizzy” and provides aftercare information (p. 833).	The video provides information across 12 scenes from pre-hospital preparation (1, 2, and 3) to arriving at the hospital and meeting the staff, preparing for surgery by changing and leaving the caregiver to walking to the operating room (4, 5, 6, and 7), to confirming the child's identity, and getting ready for surgery having equipment applied (8, 9, 10, 11, and 12). It provides information on the whole pre-operative process, hospital environment, and staff (p. 395).	Partially. The intervention uses VR-simulated steps, viewed through eyeglasses in front of a computer monitor, of going to the operation room, with headphones placed on the child's ears to stimulate the sounds of the virtual environment (p. 3). The paper states information provided on the ward and operating room but otherwise lacks details to meet this domain fully.
Skills	Partially. The movie is not interactive but only informative (p. 794); however, the paper states (p. 789) that visuals modelling body language, together with auditory information, are two key elements for effective learning methods.	Partially. The child can point at different instruments with a motion-tracked controller and the staff in the environment then explain what they are for (p. 730).	The movie is not interactive but only informative (p. 2).	The VR incorporates games to challenge the child to defeat the germ monster and awards “health points” each time the child advances to the next pre-operative step (p. 3).	Use of games and videos to build coping skills, modelling behaviour, and tailoring the experience to the child's fear levels by providing information about the surgical process; the child character Anna displays more or less fearful responses (p. 909–911).	Partially. At the end of the computer package, a list of activities is provided to support the prevention of tooth decay to achieve improved oral health (p. 833). However, insufficient information is provided to determine if other skills are addressed such as coping mechanisms.	Partially. The video is available for multiple reviews in advance of surgery during the week before, allowing the child to develop an understanding of the pre-operative process. However, the provided booklet for caregivers contains information on coping techniques, which the child did not have direct access to (p. 395).	Insufficient information is provided to judge if the VR-stimulated environment develops skills and is viewed only once lasting approx. 5 min (p. 1).
Emotion	Unclear if the movie addresses emotion. Reference made to a child asking about pain and being told that pain relief is used, but there is no indication of any emotional coping (p. 794).	The VR includes a video with a nurse explaining what kind of feelings the child may experience, for example, nausea (p. 730)	Partially. Dr Wisp explains that he is scared and shivering, and Dr Cloud tells him it is ok and that you do not need to feel like that (reference: video clip online).	Unclear if emotions are addressed in this VR game.	The intervention addresses emotion by tailoring the information provided and the fear responses of the character Anna to the child accessing the site (p. 911)	No information to suggest emotions are addressed in the detail provided in the paper. Appears to largely be informative about the process and staff involved.	The video description in the paper appears to not address emotions, with this being covered by the booklet for the caregiver (p. 395). Therefore scored 0 as it is not a part of digital intervention.	Insufficient information is provided to judge if the VR-stimulated environment addresses emotions.
Behavioural Regulation	No information provided.	Does not appear that the VR includes any behavioural regulation and is purely informative.	The video does not address any behavioural regulation.	The VR game incorporates breathing practice (p. 3.)	The intervention provides techniques for the self-management of anxiety through deep breathing and guided imagery. This is aimed at helping to reduce anxiety and lessen feelings of nervousness (p. 909 and 910). Also uses distraction techniques to help the child manage anxiety (p. 910), and module 4 provides instruction on implementing behavioural strategies to minimise pain and distress (p. 910).	The computer package does not appear to address behavioural regulation.	The video description in the paper appears to not address behavioural regulation, with this being covered by the booklet for the caregiver (p. 395). Therefore scored 0 as it is not a part of digital intervention.	Insufficient information is provided to judge if the VR-stimulated environment addresses behavioural regulation.
Memory, Attention, and Decision Processes Memory	Partially. The paper states (p. 788 and 789) that behavioural programmes to teach coping skills through modelling need to consider the child's age, developmental stage, and previous experience. It is, therefore, assumed that this was factored into the development of this programme, but it is insufficient to fully meet the domain.	Partially. Two versions of the VR video were produced to address developmental differences in children aged 4 to 12 years, possibly considering memory retention ability and comprehension.	Partially. The video is short, approx. 6 min, simple, and engaging using humour and playfulness (p. 2 and online video clip).	The VR game is short, uses famous childhood characters from the animated film ‘Hello Carbot’ and has challenges and rewards to keep the child engaged (p. 3).	The intervention uses animated characters Billy Bot and his sidekick Tot Bot to help the child navigate through the modules, using videos, games, and humour to engage the child (p. 911). A memory game is included to introduce the child to the objects in the PACU. Additional printable resources are provided to reinforce learning from web-based modules. Website is available 5 days before surgery and up to 10 days after surgery and can be accessed 24 h a day, 7 days a week (p. 913)	Unclear. While the computer package seems to be short, it is not clear from the papers whether a child's attention and memory were factored into its development, nor how long it takes to navigate the package.	Partially. The video is short at approx. 9 min and provides a week in advance for children and caregivers to access as many times as they wish, enabling information to be absorbed in a relaxed environment (p. 395). However, there are no interactive elements for this tool.	Insufficient information is provided to judge if the VR-stimulated environment addresses this domain.
Environmental Context and Resources	The video recording shows the equipment, operating room, and recovery room. It also explains what to expect in terms of how the anaesthesia mask is used by demonstrating on a teddy bear. It includes the anaesthesiologist and nurse (p. 794).	The VR video provides detailed information and images of the hospital environment, staff, and equipment (p. 730).	Partially. The video includes information on the operating room and some of the equipment and explains the use of the pulse oximeter and anaesthesia mask, but there is no information on staff or the wider hospital environment, including the recovery process (p. 2 and online video clip).	The VR storyline and games provide detailed information and images of the operating room environment, equipment, and staff (p. 3).	The intervention provides information on the hospital environment, staff, and equipment through videos and games. It also uses a specific game to introduce the child to the objects in the operating room (p. 911).	Partially. The computer package includes information on staff and explains the use of the pulse oximeter and anaesthesia mask, but no information is provided on the wider environment or other equipment (p. 833).	The video provides information on the hospital environment, staff, and equipment (p. 395).	Partially. The intervention uses VR-stimulated steps, viewed through eyeglasses in front of a computer monitor, of going to the operation room, with headphones placed on the child's ears to stimulate the sounds of the virtual environment (p. 3). The paper states information provided on the ward and operating room but otherwise lacks details to meet this domain fully.
Beliefs about Capabilities	Unclear as insufficient information is provided in the paper, and there is no indication of building confidence or esteem through coping.	Unclear as insufficient information is provided in the paper, and there is no indication of building confidence or esteem through coping.	Does not address this domain. The video is purely information provision and does not incorporate any activities or interaction to enable building self-confidence, self-esteem, etc.	Assumed through the use of the challenging game to defeat germ monster (p. 3).	The games used are designed to “model, reinforce and practice strategies for keeping calm and reducing nervousness” (p. 911).	Does not address this domain. The computer package is purely information provision and does not incorporate any activities or interaction to enable building self-confidence, self-esteem, etc.	Does not address this domain. The video is purely information provision and does not incorporate any activities or interaction to enable building self-confidence, self-esteem, etc.	Does not address this.
Beliefs about Consequences	The movie explains that pain is managed by anaesthesia and that the child will fall asleep quickly and then awake after surgery with the parent in recovery. It shows how the anaesthesia will be administered through a vessel in the hand (p. 794).	Assumed through step-by-step storyline from admission to anaesthesia, including enabling interactive motion control to seek additional information on equipment (p. 730)	Partially. The video includes information on the operating room and some of the equipment and explains the use of the pulse oximeter and anaesthesia mask. These give a sense of the consequences, but they insufficiently address this domain to score a 1 (p. 2).	Assumed through the child advancing through a step-by-step pre-operative process and interaction of the operating room environment (p. 3).	Assumed using tailoring information to the child's level of fear, through the provision of information about the surgical process and use of video and games on objects in the operating room and PACU and placing of anaesthesia masks on animals (p. 911).	The computer package includes information about staff and some of the equipment and explains the use of the pulse oximeter and anaesthesia mask, giving a sense of the consequences. It also provides a list of activities to support the prevention of tooth decay to achieve improved oral health (p. 833).	Partially. The video includes information on the pre-operative process, including the equipment and staff, but insufficiently addresses this domain to score a 1 (p. 395).	Partially. The VR-stimulated environment includes information on the steps of going into the operating room but does not fully address this domain to score a 1 (p. 3).
Reinforcement	A simple video explaining specific information about the pre-operative expectations, but there is no use of interactive tools, nor is there any implication whether the child could rewatch the video.	Although the VR video uses interactive motion–controlled gesturing, this is simply to enable the further provision of information, and there is no indication that any behaviour reinforcement is applied.	A simple video explaining specific information about the pre-operative expectations, but there is no use of interactive tools, nor is there any implication whether the child could rewatch the video.	The VR uses interactive game activities after instructions to challenge the germ monster; it also includes the choice of a facial oxygen mask to practice breathing (p. 3).	The use of deep breathing exercises, the use of memory games for introducing the child to the objects in the PACU, and placing the anaesthesia mask on animals all provide reinforcing behaviours (p. 911). Additional printable activities, including colouring sheets, are also provided.	A simple cartoon computer package explaining specific information about staff and some of the processes, but there is no use of interactive tools, nor is there any implication whether the child could rewatch the sequence.	Partially. The video is available for multiple reviews in advance of surgery during the week before, allowing the child to develop an understanding of the pre-operative process (p. 395).	Does not address this.
Intentions	Does not address this.	Does not address this.	Does not address this.	Partially. Using breathing exercises and interactive gameplay with rewards (p. 3).	Partially. Using breathing and coping exercises for both child and parents (p. 909 and 911).	Does not address this.	Does not address this.	Does not address this.
Goals	Does not address this.	Does not address this.	Does not address this.	The VR game element uses rewards when the child advances to the next step (p. 3).	Provision of certificate for completion of the program, advancement through the modules by playing games (p. 911).	Partially. At the end of the computer package, a list of activities is provided to support the prevention of tooth decay to achieve improved oral health (p. 833).	Does not address this.	Does not address this.
Social influences	Does not address this.	The VR video confirms that parents can stay with the child all the time until they are anaesthetised; it includes images of the parent and child wearing hospital gowns (p. 730).	Does not address this.	Partially. The VR game incorporates a famous animated character to socialise the content in a child-friendly manner (p. 3).	Parental modules of the site aimed at teaching parents coping and modelling behaviours, with this part accessed before the child site, which parents then go through with their child (p. 908 and 909).	Does not address this.	Does not address this.	Does not address this.
Optimism	Not demonstrated.	Does not address this.	Does not address this.	Assumed through rewarding “health points” when child advances levels (p. 3).	Partially met. Use of tailoring to create feelings of being safe and managing anxiety levels. Use of memory games to introduce objects in the PACU and provision of completion certificate.	Does not address this.	Does not address this.	Does not address this.
Co-production with: - healthcare professionals - children - parents	No information is provided on the development of the video.	PARTIAL: A multidisciplinary team, consisting of child life specialists, child psychologists, a child psychiatrist, anaesthesiologists, a 3D acting director, and a 3D project manager, designed the script of the VRE (p. 2). The paper indicates that it is tested by children and adjusted to consider feedback, but there is no indication of parental involvement.	Beyond confirming that the video was produced in collaboration with the association of Soccorso Clown, it does not confirm if healthcare staff, children, or parents were involved. While this may be assumed, this is not stated.	PARTIAL: The study authors developed the video in collaboration with the VR game producing company, with the study authors comprising healthcare professionals (confirmed *via* searching).	YES: The programme was developed using a task force of anaesthesiologists, psychologists, surgeons, nurses, paediatricians, child life specialists, parents, and children [p. 906, Kain et al. (2015) paper].	No information is provided on the development of the computer package, and the referenced paper is not accessible.	YES: The video was developed and edited by various medical staff (outpatient unit, surgical ward, and operation department) and reviewed by families and experts (p. 395).	No information is provided on the development of the VR-stimulated environment.
Used a behaviour framework	No information is provided on the development of the video and therefore on the use of any behavioural frameworks.	Unclear. While various experts were used to inform development, including psychologists, it is unclear if any behaviour frameworks were used.	No information is provided on the development of the video and therefore on the use of any behavioural frameworks.	No information is provided on the development of the VR games and therefore on the use of any behavioural frameworks.	Yes. Paper Kain et al. (2015) states that the “conceptual framework and content of WebTIPS was examined by a behavioural medicine, interventions, outcomes expert panel” (p. 906).	No information is provided on the development of the computer package, and the referenced paper was not accessible.	No information is provided to determine of any theoretical frameworks were used.	No information is provided.

VR, virtual reality; App, smartphone, or tablet, or computer applications; 3D, three-dimensional; m-TDF, modified theoretical domains framework; PACU, Post Anaesthesia Care Unit; VRE, virtual reality exposure.

### 3.4. Overview of the domains met in DHIs

None of the 15 domains was fully evidenced across all the DHIs, with 35% evidencing eight or more domains and 65% evidencing seven or fewer domains. The DHIs by Wright et al. (62) and Fortier et al. (71) fully evidenced the most domains, with 13 met in each. The first nine domains outlined in Table 3 were met in each of these three studies, with differences occurring in the remaining six domains, namely, intentions, goals, social influence, optimism, co-production, and use of a behaviour framework. Stunden et al. (59) scored the next highest fully evidencing 11 domains, meeting the first nine and those for intentions and goals. Ryu et al. (70) scored the next highest, fully evidencing 10 domains, with all but that for emotion in the first nine being met, as well as goals and optimism. Dehghan et al. (74) did not evidence any domains fully, and the DHIs used by Huntington et al. (65), Campbell et al. (72), and Wakimizu et al. (73) fully evidenced only one domain and two domains each, respectively. This was attributed to insufficient information, as opposed to simply not meeting the domain. The remaining DHIs varied, with between three and nine domains fully evidenced. On average, the DHIs fully met 5.4 domains with a standard deviation of 4.17 and partially met 2.5 domains with a standard deviation of 1.19.

#### 3.4.1. Domains for knowledge, beliefs about consequences, and environmental context and resources

The highest scoring modified TDF domains were for knowledge, beliefs about consequences, and environmental context and resources, with these being fully evidenced in 13, 11, and 10 DHIs, respectively. The domains for knowledge and environmental context and resources were the only two domains to have either been fully or partially evidenced for all 15 DHIs. The belief about consequences domain was fully or partially evidenced for 14 DHIs. Two DHIs did not fully meet the domain for knowledge. Liguori et al. (69) provided information on the operating room and equipment, lacking detail on the staff involved and the wider hospital environment, including what to expect before and after the procedure. Dehghan et al. (74) simply stated that “[the DHI] presented the simulated steps of going to operation room … [with] simulated sounds …” (p. 3). It was, therefore, deduced that while some information on the hospital environment was provided, insufficient detail was available on the whole experience and what elements were contained within the simulated steps to score fully. For the same reasons, these two studies were two of the five DHIs not fully meeting the domain for environmental context and resources. In contrast, Wantanakorn et al. (64), Huntington et al. (65), and Campbell et al. (72) all scored fully on knowledge but partially on environmental context and resources. Compared with the other 10 DHIs, the information in these DHIs lacked a wider environmental context and less detailed descriptions of all resources involved in the procedure.

The criteria to assess the beliefs about the consequences domain were dependent on the level of information provided to create an understanding of what the child would experience. Of the 15 DHIs, this domain was evidenced fully in 11, partially in three, and inconclusively in one. The DHIs scoring fully (58–64, 66–68, 70–72) either gave a step-by-step guide of what would happen, what and who were involved, and often what feelings or experiences may occur or used level progression or interactive games to check understanding. The three DHIs (69, 73, 74) scoring partially provided some information on what would happen but lacked information on feelings or experiences. Insufficient information was available on the Huntington et al. (65) DHI to score this domain.

#### 3.4.2. Domains for optimism, intentions, goals, and social influences

The lowest scoring modified TDF domains were for optimism, intentions, and goals, with these being fully evidenced in 1, 2, and 3 DHIs, respectively. They were equally the lowest to score either fully or partially for all DHIs at 3, 5, and 4, respectively. The optimism domain was assessed on the basis of the inclusion of rewards or attainments in the DHI. It scored the least across all DHIs, with one (70) scoring fully because of awarding health points when the child advanced through the DHI levels and two (59, 71) scoring partially, as they separately incorporated level attainment and a completion certificate. The domains for intention, goals, and social influences were the next lowest scoring across all DHIs. Intentions were assessed on the basis of whether the DHI utilised feedback or rewards, goals if specific actions or behavioural changes were integrated, and social influences on whether something was aimed at parents or whether it used familial exposure or famous characters. The use of interactive real-time feedback to enable level progression and specific behavioural components scored two DHIs fully (59, 62), whereas the use of breathing or coping exercises partially scored three DHIs (64, 70, 71) for intention. The goals domain scored fully in three DHIs (59, 70, 71) that utilised feedback, level progression, and/or rewards and partially in one DHI (72). The social influences domain scored fully in four DHIs, with two specifically including a parental element in the DHI (62, 71) and two (58, 68) requiring parental involvement more broadly. Three DHIs scored partially on social influence either using famous characters (60, 61, 63, 70) or addressing parental separation (67).

#### 3.4.3. Domains for skills, reinforcement, emotion, behaviour regulation, beliefs about capabilities, and memory, attention, and decision processes

Except for emotions, these domains appertain to building skills or techniques to address behaviour and emotions, with this being achieved through interactive elements such as games, exercises, or activities. Emotion is linked both as a contribution to, and an outcome of, these domains. The scoring for the remaining modified TDF domains was mixed across the DHIs. The skills and reinforcement domains were evidenced fully in the same seven DHIs (58, 59, 62, 64, 67, 70, 71), as they integrated interactive games or actions, building skills, and understanding. However, five DHIs partially evidenced skills because of including modelling videos such as breathing exercises (65, 66) or information (68) or activities (72) or those that could be viewed multiple times (73), while only Wakimizu et al. (73) partially evidenced reinforcement. The domains for emotion, behavioural regulation, and beliefs about capabilities all scored fully in six DHIs, with the score being the same for four of them (58, 59, 62, 71). The full scoring DHIs for the other two in each of these domains differed, with Wantanakorn et al. (64) and Ryu et al. (70) fully evidencing behavioural regulation and beliefs about capabilities and Fernandes et al. (67) and Eijlers et al. (68) fully evidencing emotion. The domain for memory, attention, and decision processes scored fully (59, 62, 67, 70, 71) and partially (58, 66, 68, 69, 73) for five DHIs each.

#### 3.4.4. Domains for co-production and use of behaviour frameworks

Of the 15 DHIs, 11 reported the design that involved co-production, with the remaining four (66, 69, 72, 74) not stating anything. The use of co-production to design and/or test the DHI with healthcare professionals, parents, and children occurred for five DHIs (58, 62, 65, 71, 73). Partial co-production with healthcare professionals and testing with children occurred for three DHIs (64, 67, 68) and with only healthcare professionals for two DHIs across four studies (59–61, 63, 70). The use of a behaviour framework was applied in the development of four DHIs across six studies (58, 60, 61, 63, 67, 71). Insufficient information was commonly the reason for the remaining six DHIs scoring 0 in this domain.

### 3.5. Study measurements, outcomes, and direction of effect calculations

All studies assessed the outcomes of the intervention, with these being self-reported by children and parents, observed by clinicians or researchers, or extracted from medical records. The primary and secondary outcomes included assessments across five categories:

1. emotions and feelings,
2. behavioural responses,
3. physiological responses,
4. clinical status, and
5. assessment of the DHIs’ usability, satisfaction, and/or knowledge.

Supplementary Table S9 in Appendix F outlines the assessment types used in each category and the studies in which they were applied. These are further divided within these categories where feasible to show results with an effect and no effect for children and parents, with observations noted between the study findings and the assessment results of the DHI. Supplementary Table S10 in Appendix F provides details of the primary and secondary outcome measures for each study, including when and how the outcomes were measured. The table includes, where feasible, the results of the effect size calculations and the main findings. This information is summarised in the following sections.

#### 3.5.1. Emotions and feelings

Emotions and feelings were assessed using 10 different measures across 11 studies (58, 59, 62–65, 67, 68, 71–73), with most of these studies using a visual analogue scale (VAS) or the State-Trait Anxiety Inventory (STAI).

##### 3.5.1.1. Effect demonstrated

Bray et al. (58) revealed that the child's self-reported VAS trait and state anxiety before the procedure were comparable between the DHI group (DHIG) and the control group. No significant difference or effect was found in either the trait (*p* = 0.85, *d* = 0.07) or the state (*p* = 0.54, *d* = 0.14) anxiety between groups. State anxiety on arrival at the hospital was significantly lower in the DHIG with a negative medium effect (*p* = 0.008, *d* = 0.61) compared with that in the control group. Similarly, Wantanakorn et al. (64) revealed that self-reported anxiety VAS scores significantly changed from one hour before the intervention (*p* = 0.82) to after its application (*p* = 0.012) within the DHIG, with a negative medium effect (*d* = 0.6). This suggests that the DHIs positively impacted levels of anxiety in these two studies. It is noted that both DHIs included interactive elements and scored fully in the domains for behavioural regulation, beliefs about capabilities, and reinforcement. However, Wantanakorn et al. (64) only partially scored for co-production and provided no evidence of the use of a behavioural framework, whereas Bray et al. (58) scored fully in both of these domains. Wright et al. (62) showed that parental self-reported STAI for trait anxiety (STAI-T) was similar between the DHIG and the two control groups 1 week before the procedure (*d* = 0 and *d* = 0.03). Parental self-reported state anxiety (STAI-S) increased pre-procedure and decreased post-procedure but with a notable increase in anxiety in the DHIG compared with that in the two control groups. A medium positive effect occurred between the DHIG and control group 1 (*d* = 0.58) and a small positive effect between the DHIG and control group 2 (*d* = 0.48) pre-procedure, changing to a small positive effect compared with control group 1 (*d* = 0.43) and no effect compared with control group 2 (*d* = 0.15) post-procedure. Fernandes et al. (67) assessed child worry and feelings by using the Child Surgery Worries Questionnaire (CSWQ) and Self-Assessment Manikin (SAM). The CWSQ results showed that children in the DHIG had significantly lower mean levels of worry compared with the two controls (no intervention and video game) across all parts of the questionnaire (*p* < 0.001). This translated into a large negative effect between the DHIG and each of the controls. In addition, the video game control group had lower levels of worries compared with the no intervention control group. SAM results showed no significant differences in valence (calmness) or arousal (happiness) between the groups before and after the interventions. Despite this, a small effect (*d* = 0.25) was calculated between the DHIG and control group 1 for valence post-intervention. For arousal in the DHIG compared with the control groups, a small effect occurred pre-intervention (*d* = 0.20 and *d* = 0.4) and a medium effect post-intervention (*d* = 0.53, *d* = 0.64). Parental anxiety in the DHIG was significantly lower with a negative medium effect compared with that in control group 1 (*p* = 0.033, *d* = 0.53) but comparable with no effect compared with that in control group 2 (*d* = 0.08). This DHI was developed using a behavioural framework and co-production with children and healthcare professionals. It also met the modified TDF domains for emotion and reinforcement, scoring fully across seven domains. Fortier et al. (71) parental self-reported STAI anxiety was significantly lower (*p* = 004) in the DHIG than in the control group pre-procedure and post-intervention, with a medium negative effect (*d* = 0.65). Anxiety remained lower in the DHIG at separation but was not statistically significant and had a small negative effect (*d* = 0.25). Wakimizu et al. (73) showed child anxiety using the Wong–Baker Faces Scale (FACES) at seven time points from before intervention (baseline) to 1 month after the procedure. The results show that children in the DHIG had lower anxiety at all time points compared with those in the control group. However, a clear small effect occurred only pre-operatively (*d* = 0.45) and 1 month after the procedure (*d* = 0.27), and a partial small effect occurred at 1 week after the procedure (*d* = 0.2). Wakimizu et al. (73) also found that parental anxiety using the STAI was lower in the DHIG at all time points with a negative medium (*d* = 0.60) effect post-operatively and a negative small effect (*d* = 0.23) at 1 week after the procedure, and all other time points showed no effect. Campbell et al. (72) found self-reported child VAS anxiety scores comparable (*p* = 0.790) before the intervention across all three groups (usual care control group 1, cartoon control group 2, and web-based click-through presentation DHIG). However, during induction and recovery, the observer-rated child VAS to determine anxiety levels shows a decrease in anxiety across all groups over time. A significant change was noted between the DHIG and control group 1 at induction (*p* = 0.014) and between the DHIG and control group 2 at recovery (0.016). The effect could not be calculated because of a non-normal distribution of data. While the results of these two studies suggest that the DHI had some impact, albeit a small effect for Wakimizu et al. (73), it is noted that both scored poorly against the modified TDF, meeting two domains fully and four and three domains only partially. Neither was the DHI interactive nor did it include aspects related to emotions or behavioural regulation. Park et al. found that the Numerical Rating Scale (NRS) for parental anxiety decreased significantly (*p* = 0.009) in the DHIG post-intervention and with a negative medium effect (0.67).

##### 3.5.1.2. No effect demonstrated

Stunden et al. (59) found no change in child anxiety before the use of the three group interventions and after the MRI simulation, with control group 1 using the Standard Preparatory Manual (SPM), control group 2 using the CLP, and the DHIG using VR-MRI. The results before preparation were SPM (median 0, IQR 1, SD 1.521); CLP (median 0, IQR 0, SD 1.240); and VR-MRI (median 0, IQR 1, SD 1.311) and those after MRI simulation were SPM (median 0; IQR 1, SD 1.738); CLP (median 0, IQR 0, SD 0.468); and VR-MRI (median 0, IQR 1, SD 0.434). It is noted that median anxiety levels increased slightly in the SPM group after preparation (median 1, IQR 2, SD 2.311) compared with CLP (median 0, IQR 0, SD 1.350) and VR-MRI (median 0, IQR 1, SD 0.819). In contrast to child anxiety levels, Stunden et al. (59) found no significant difference in parental anxiety across the three time points, although it did increase from before to after preparation and decreased again after the MRI simulation in both control groups. Of interest to these findings is that this study scored highly against the modified TDF despite not demonstrating the use of a behavioural framework; however, the RoB2 results were high due to the potential for allocation sequence knowledge, potentially influencing the results. Huntington et al. (65) also found no change in child anxiety using the Facial Image Scale (FIS) over time, with the results comparable among all three groups, with control group 1 using usual care, control group 2 using a handwashing game, and the DHIG using a web-based click-through presentation. Eijlers et al. (68) found no significant difference in child self-reported VAS anxiety between the DHIG and the control groups at all four time points, measured before the intervention (*p* = 0.407) and after (*p* = 0.753, *p* = 0.735, *p* = 0.727). Likewise, self-reported STAI and observed VAS parental anxiety were comparable between the control group and the DHIG immediately after child induction with no effect observed in the STAI results (*d* = 0.01). Campbell et al. (72) parent-reported Modified Child Anxiety Scale (MCDAS) scores indicated higher child anxiety levels than those self-reported by children but were not statistically significant among the three groups.

#### 3.5.2. Behavioural responses

Behavioural responses were assessed using 11 different measures across 12 studies (60–66, 68–71, 74). All these 12 studies measured behaviour change using the Yale Preoperative Anxiety Scale (YPAS), with 11 of these using a modified YPAS (m-YPAS). Three studies (60, 68, 71) measured Paediatric Anaesthesia Emergence Delirium (PAED) and four studies (61–63, 70) measured IC.

##### 3.5.2.1. Effect demonstrated

The DHIs used by Wright et al. (62) and Fortier et al. (71) were both web-based programs available for multiple uses in the week before the child's procedure at home. Both DHIs scored fully for co-production and use of a behavioural framework. Wright et al. (62) observer-rated m-YPAS child anxiety scores were lower in the DHIG [I-Paediatric Preparation Programmes (PPP)] than in the two control groups (usual care and I-PPP + parent). This correlated to a small negative effect (*d* = 0.24) between the I-PPP and the usual care groups in the holding area and to a medium negative effect (*d* = 0.53) and small negative effect (*d* = 0.34) between the DHIG and the usual care and I-PPP + parent groups, respectively. The lower anxiety levels in both the I-PPP and the I-PPP + parent groups to the usual care group suggest that the DHI positively impacted anxiety levels. When considered against the higher parental anxiety STAI-S scores in the control groups, it was possible that parental anxiety may have impacted child anxiety. Fortier et al. (71) found a significant difference in observer-rated m-YPAS child anxiety scores across groups and time. At separation to the operating room scores were comparable among groups, but in the DHIG, anxiety decreased at the entrance to the operating room (*p* = 0.02, *d* = 0.59) and again considerably during induction (*p* = 0.01, *d* = 0.63). Parental STAI anxiety scores followed a similar trend to that of the children. The DHI used by Hatipoglu et al. (66) was a video viewed once, a week before the procedure in the hospital. Compared with the two control groups (usual care and voice recording), observer-rated m-YPAS child anxiety was significantly lower in the DHIG (*p* < 0.001). A large negative effect was calculated between the DHIG and the control groups, respectively (*d* = 3.34, *d* = 0.822). The DHIs used by Wantanakorn et al. (64) and Liguori et al. (69) were used the day before the child's procedure. Both studies showed a significant decrease (*p* = 0.001, *p* = 0.009) in observer-rated m-YPAS child anxiety after the use of the DHI in the DHIG compared with the control group. A medium negative effect (*d* = 0.6) and large negative effect (*d* = 0.9) were calculated. Ryu et al. (60, 61, 70) and Park et al. (63) measured pre-operative child anxiety using the Korean m-YPAS. All these studies found a significant difference (*p* = 0.022, *p* < 0.01, *p* < 0.001, and *p* = 0.025, respectively) between the DHIG and the control group after the use of the DHI 1 h before the procedure, with negative effects of small (*d* = 0.47) and large (*d* = 0.80) in the first two. The effect could not be calculated for Ryu et al. (70) and Park et al. (63) because of the non-normal distribution of data. Dehghan et al. (74) reported that child anxiety was significantly different in all domains, except in arousal, in the two DHIGs. No effect size could be calculated because of the nature of the reported data. For induction behaviour and compliance, Ryu et al. (61) found significantly lower Procedural Behaviour Rating Scale (PBRS) scores during induction in the DHIG (*p* = 0.01). Ryu et al. (61, 70) and Wright et al. (62) measured induction compliance using the Induction Compliance Checklist (ICC). A higher compliance was found in the DHIG than in the control groups (*d* = 0.86, *d* = 0.52, *d* = 0.54). Fortier et al. (71) measured emergence delirium using the PAED and found that it was significantly lower in the DHIG (*p* = 0.04), with a small negative effect (*d* = 0.45). Post-operative behaviour was measured by Hatipoglu et al. (66) using the Post-Hospitalisation Behaviour Questionnaire (PHBQ) 7 days post the procedure. They found a significant difference (*p* < 0.001) between control group 1 (usual care) and both control group 2 (voice recording) and the DHIG. The effect size between the DHIG was large to control group 1 (*d* = 2.049) and small to control group 2 (*d* = 0.31). In addition, they showed that anxious children had a 1.03 times greater risk of adopting negative post-operative behaviours.

##### 3.5.2.2. No effect demonstrated

Eijlers et al. (68) found no significant differences in self-reported or observer m-YPAS anxiety scores between the DHIG and the control group after intervention use on the same day, with results comparable across all time points. Equally, no effect was noted where it could be calculated because of the normal distribution of data, with *d* = 0.02 at admission before intervention and *d* = 0.01 in the holding area after the intervention. Although the intervention was used a week before the procedure, Huntington et al. (65) found no significant difference in m-YPAS child anxiety scores between the DHIG and the two control groups overall. A small positive effect (*d* = 0.21) was calculated between the DHIG and control group 2 (handwashing game) both pre- and at induction. For induction behaviour and compliance, Ryu et al. (70) found PBRS scores during induction comparable between the groups (*p* = 0.92). Huntington et al. (65) found no difference in induction behaviour using observer-rated VAS between the DHIG and the control groups, correlating with no effect (*d* = 0, *d* = 0.08). Park et al. (63) ICC results found compliance similar between the groups (*d* = 0.07). Ryu et al. (60) and Eijlers et al. (68) also measured emergence delirium. Both found no significant difference in PAED scores between the DHIG and the control group (*p* = 0.719, *p* = 0.266). For behaviour, Ryu et al. (60) used the PHBQ-AS at one and 14 days post-operatively, finding no significant difference (*p* = 0.671, *p* = 0.329) among children in the two groups. Eijlers et al. (68) used the Child Behaviour Checklist (CBCL) at admission, and no statistical significance was found between the groups (*p* = 0.251).

#### 3.5.3. Physiological responses

The study by Fernandes et al. (67) was the only one to measure physiological changes before and after the intervention and also after the SAM measurements. Blood pressure was similar, with no effect among all three groups, although mean values were lower in the DHIG. The heart rate was similar between the control groups and lower in the DHIG, with a small negative effect pre-intervention (*d* = 0.45, *d* = 0.36) increasing to a medium negative effect post-intervention (*d* = 0.53, *d* = 0.63) in the DHIG compared with the control groups.

#### 3.5.4. Clinical status

Clinical status was assessed in five studies (59, 64, 65, 68, 71) with measures including pain level, length of stay, medication usage, head movement in MRI simulation, and MRI preparation and assessment time. Child pain was measured by Eijlers et al. (68) using the observer-rated Face, Legs, Arms, Cry, Consolability scale (FLACC) in recovery, and the Parents’ Postoperative Pain Measure (PPPM) at home, and Fortier et al. (71) used an NRS. No statistical significance was found in any of these measures between the DHIG and the control group in both studies, with the results being *p* = 0.410, *p* = 0.454, and *p* = 0.30, respectively. For patient flow, Huntington et al. (65) measured anaesthetic induction time, recovery time, and ward time, finding no significant difference among the three groups. However, the DHIG had a slightly longer recovery time than control group 2 (handwashing game) with a small positive effect (*d* = 0.31) and spent less time on the ward compared with control group 1 (usual care) with a small negative effect (*d* = 0.28). Fortier et al. (71) similarly found no significance between the groups for surgery (*p* = 0.708) or recovery (*p* = 0.26) time. Medication usage for analgesic consumption was recorded by Fortier et al. (71) and Eijlers et al. (68) and for sedative drugs by Wantanakorn et al. (64), with all of them finding no significant difference between groups overall. Eijlers et al. (68) found that DHIG children undergoing an adenoidectomy and tonsillectomy needed significantly less rescue analgesic compared with the control group (*p* = 0.002, *d* = 0.46), and overall, a small effect (*d* = 0.22) was calculated between the need for rescue analgesia in the DHIG compared with the control group. Stunden et al. (59) used head movement in the MRI simulation to determine success with a threshold of 3–4 mm. They found no statistically significant difference in the number of participants scoring above the threshold (*p* = 0.07) nor among the three groups (*p* = 0.27). The chi-square *p*-value effect calculated a small effect (*d* = 0.43) in average successful MRI and a small negative effect (*d* = 0.26) between the groups, with the DHIG (VR-MRI) being on average less successful at 30% compared with control group 1 (SPM) at 47% and control group 2 (CLP) at 50%. Preparation time and assessment time were measured in minutes. Preparation time between the groups was significantly different (*p* < 0.001) and had a medium effect size (*η*^2 ^= 0.57), with the DHIG preparing the longest on average at 22.05 min. However, assessment time was comparable across the groups with no significant difference (*p* = 0.13).

#### 3.5.5. Assessment of the DHIs’ usability, satisfaction, and/or knowledge

DHI usability, satisfaction, and/or knowledge and understanding were assessed using seven different measures across eight studies (58, 59, 61–63, 65, 70, 73). Bray et al. (58) measured procedural knowledge and satisfaction of children and parents or caregivers using the VAS. Procedural knowledge was measured 3–5 days before the procedure and on arrival at the hospital, increasing significantly for both children (*p* < 0.001) and parents or caregivers (*p* = 0.01) in the DHIG compared with the control group. The calculated effect was positively large for children (*d* = 1.11) and positively medium (*d* = 0.59) for parents and caregivers. Procedural satisfaction in children and parents was not statistically significant (*p* = 0.10 and *p* = 0.72) but was higher in the DHIG than in the control group, with a small positive effect in children (*d* = 0.37). Stunden et al. (59) measured child satisfaction using the VAS and found that children in control group 2 (CLP) and the DHIG (VR-MRI) were on average more satisfied than children in control group 1 (SPM) at 90%, 80%, and 73.5%, respectively. Huntington et al. (65) measured parental satisfaction using the VAS, reporting results only for those scoring 9 or 10 across the three groups, but they found no difference with the scores comparable. In addition, Huntington et al. (65) evaluated treatment by applying the Treatment Evaluation Inventory 48 h after the procedure and found that the DHIG had a higher odds ratio (OR) for satisfaction relative to control group 1 and control group 2 for whether they found the information helpful for their child to handle the visit (OR = 12; 95% CI 4.7–32, *p* < 0.001 and OR = 8.2; 95% CI 3–22, *p* < 0.001) and whether it improved their child's ability to cope (OR = 21; 95% CI 8–56, *p* < 0.001 and OR = 13; 95% CI 5–34, *p* < 0.001). Ryu et al. (61, 70) used an NRS to measure parental satisfaction and found no significant difference between the DHIG and the control group (*p* = 0.198, *p* = 0.268). Park et al. (63) did find a significant difference in NRS scores for parental satisfaction (*p* = 0.008). Wright et al. (62) measured parental satisfaction using the Client Satisfaction Survey and found that parents in control group 2 (I-PPP + parent) were more satisfied than their counterparts in control group 1 (SPM) and the DHIG (I-PPP). With regard to the DHIG, a small positive effect (*d* = 0.20) was calculated against control group 1 and a medium negative effect (*d* = 0.50) was calculated against control group 2. Stunden et al. (59) assessed how fun children found the interventions using the Smilyometer, with children in control group 1 (SPM) finding it “okay” and those in control group 2 (CLP) and the DHIG (VR-MRI) finding it “really good”. They also assessed parental usability of the interventions using the Usefulness, Satisfaction and Ease of use (USE) questionnaire. No significant difference was found among the three groups, with control group 1 agreeing that it was somewhat useful but easy to use and learn and control group 2 and the DHIG agreeing that it was useful, easy to use, and learn. Bray et al. (58) used a 5-point Likert scale to measure self-reported child procedural involvement and a tick-box form against the parts of the App that the children looked at and liked. They found procedural involvement slightly higher in the DHIG than in the control group (*p* = 0.03), and of the 20 children who completed the form, they liked the different components. Wakimizu et al. (73) used a 4-point scale to measure parental satisfaction in the DHIG and found that the majority (*n* = 66, 91.7%) were satisfied.

### 3.6. Risk of bias assessment

**Figure 2:**
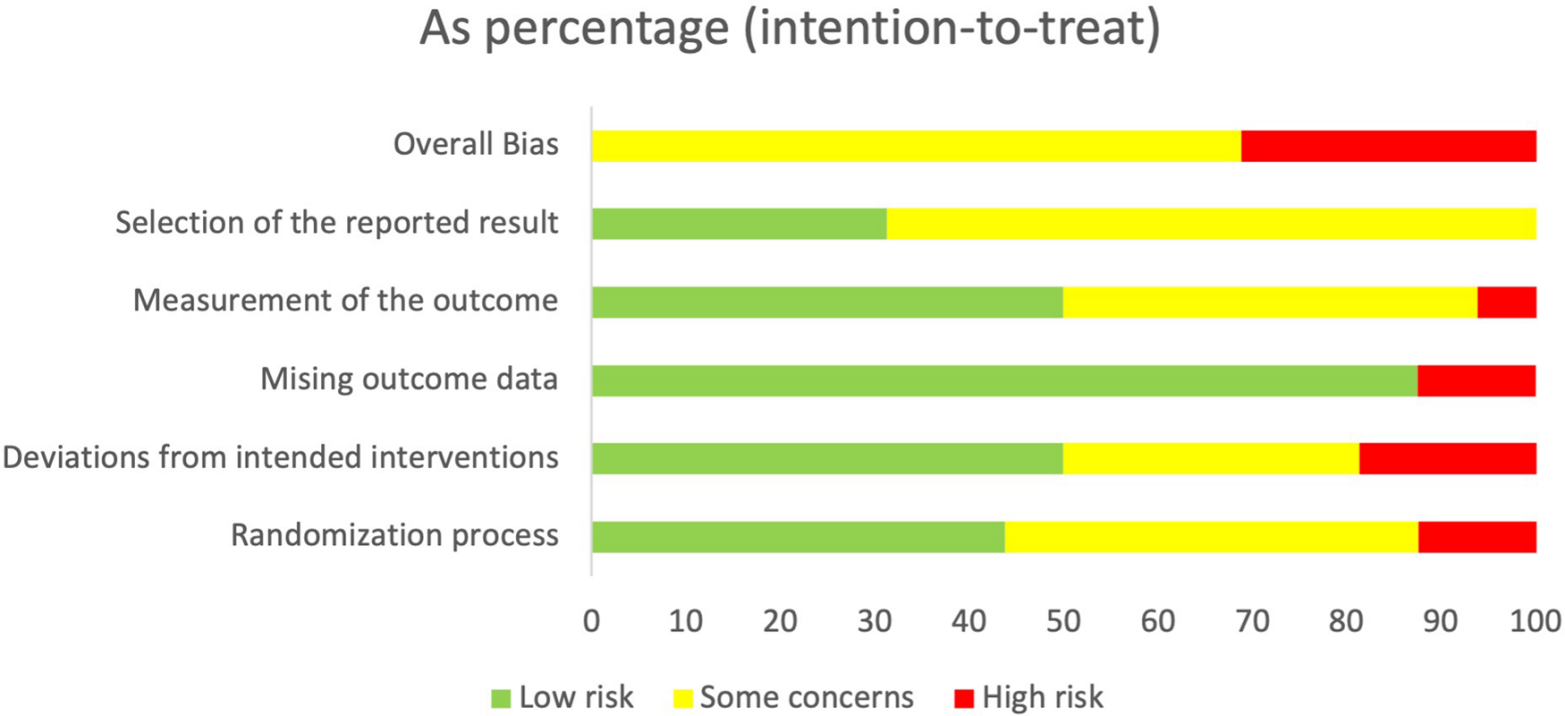
Risk of bias results as a percentage.

The risk of bias across the 16 randomised control trials was generally concerning, with 68.8% having an overall result of some concern (60–63, 65–70, 73) and 31.1% an overall result of high risk (59, 64, 71, 72, 74). Risks were linked to the process for randomisation or the inability to confirm whether a pre-specified analysis plan was finalised before the results were unblinded for analysis. Figure 2 provides an overall summary of bias as a percentage for the six domains.

**Figure 3:**
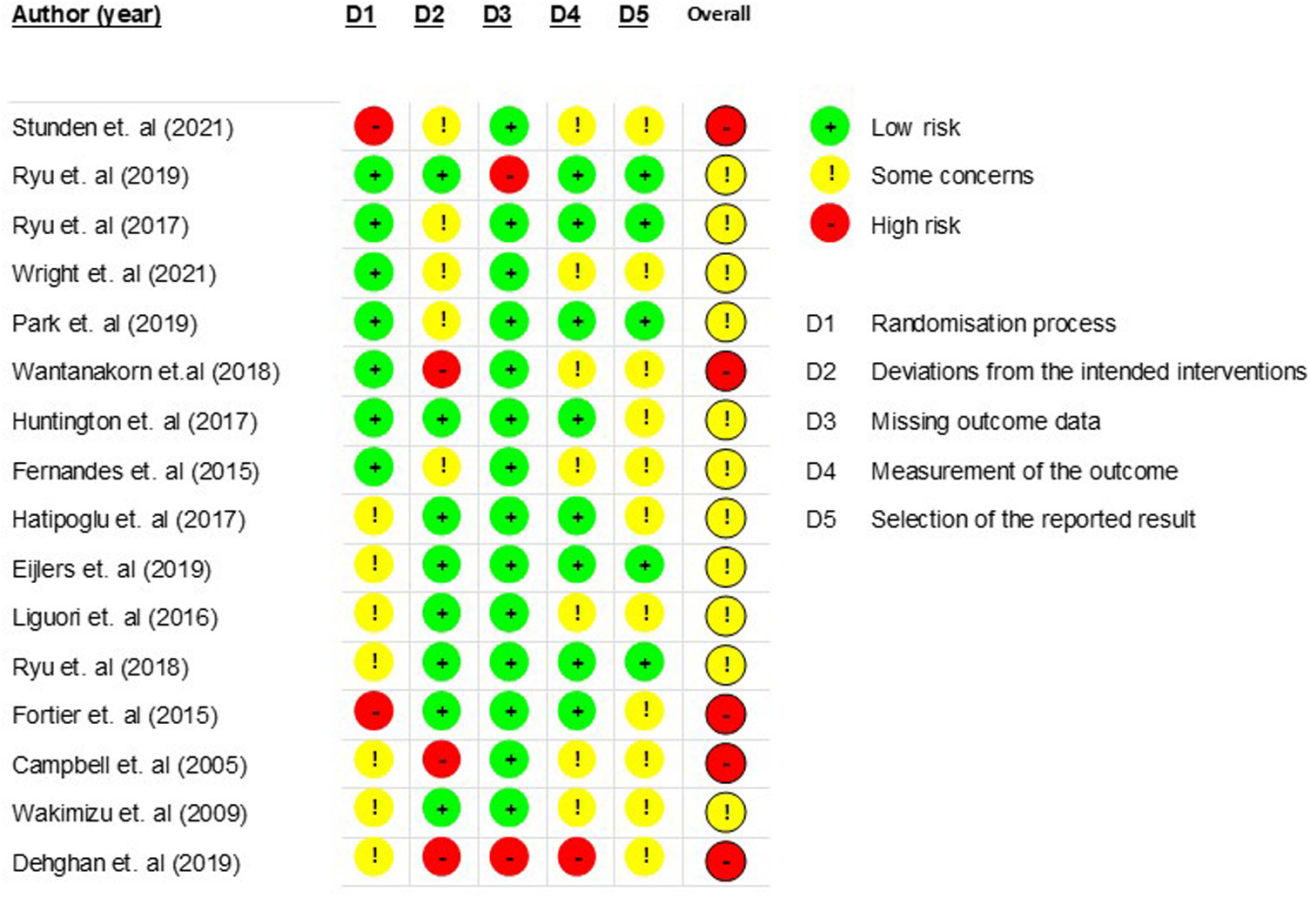
Risk of bias assessment for each included study.

Figure 3 provides a breakdown of the risk of bias for each study against the six domains, namely randomisation process (D1), deviations from intended interventions (D2), missing outcomes data (D3), measurement of the outcomes (D4), selection of the reported results (D5), and overall bias.

All studies used a random group allocation sequence, with this being computerised in eight studies (60–63, 66, 67, 70–72). The method of randomisation varied in the rest of the studies, including drawing lots, using concealed envelopes, allocating on bed numbers, or using an allocation ratio. Randomisation process bias (D1) for seven studies (60–65, 67) was low, with this being attributed to confirmed allocation sequence concealment and no noted baseline differences among the groups to suggest problems. Conversely, seven studies (66, 68–70, 72–74) were determined as having some concern due to insufficient information on the allocation sequence concealment but no notable baseline differences among the groups. Dehghan et al. (74) provided insufficient information to determine whether baseline differences among the groups suggested a problem with the randomisation process. Due to the potential for allocation knowledge to influence participant bias, the studies by Stunden et al. (59) and Fortier et al. (71) were determined to have a high risk of randomisation process bias, as both confirmed that blinding to allocation was not possible.

Bias due to deviations from intended interventions (D2) was low across 50% of studies. Of the five studies considered to have some concern of bias in this domain, three (59, 62, 63) were attributed to insufficient information on deviations from intended intervention groups. Ryu et al. (61) had one deviation from the DHI group due to dizziness using the VR, although the child was not reassigned and was excluded from the analysis. Fernandes et al. (67) reassigned 15 children after randomisation because of ethical concerns over children sharing the same ward and being in different groups. The potential bias from this change in the group was deemed to be of some concern but not high risk, as participants were unaware of their group allocation until receiving the intervention. Wantanakorn et al. (64), Campbell et al. (72), and Dehghan et al. (74) were considered at a high risk of bias in this domain because of insufficient information to determine whether participants, carers, and people delivering the interventions were aware of group assignment, whether any deviations from the intended groups occurred, and whether an appropriate analysis was used to estimate the effect of assignment to intervention.

Bias due to missing outcome data (D3) was low across 88% of the studies and considered high for two studies. Ryu et al. (60) excluded three participants from analysis because of a data collection failure, and given the small sample size, it was considered that this could have impacted the outcomes, thus having a potentially high risk of bias. Dehghan et al. (74) provided insufficient information on whether data were available for all or nearly all participants, thus also having a higher risk of bias.

Bias for measurement of outcome (D4) was deemed low in 50% of studies (60, 61, 63, 65, 66, 68, 70, 71) as the same appropriate outcome measures among the groups were used and the outcome assessors were blinded. In contrast, 43.8% of studies either provided insufficient information to conclude whether the outcome assessors were blinded (67, 69, 73) or provided evidence to suggest that they were not blinded (59, 62, 64), resulting in some concern of bias. Campbell et al. (72) likewise had some concern of bias in this domain, but this was due to an inability to align the sample size in the result data, meaning insufficient information was provided to decide whether measurement or ascertainment of the outcome differed among the groups. Although the same appropriate measures were used for the outcomes among the groups in Dehghan et al. (74), insufficient information was provided to determine whether the outcome assessors were blinded. As a knowledge of group interventions could lead to bias, and it was not possible to determine whether it was likely that the outcomes could have been influenced by this knowledge, it was considered that this study was at a high risk of bias.

Most studies (68.8%) had some concern for bias in D5 “selection of the reported results”. This was due to an inability to confirm whether the outcome data were analysed following a finalised pre-specified analysis plan before unblinded outcome data were made available for analysis. This according to Cochrane RoB2 guidelines (94) means that there is an unclear risk for reporting bias. For 10 studies (62, 64–67, 69, 71–74), a trial protocol was not obtained, and although the studies generally set out the analysis plan, it was not viable to confirm whether it was finalised before unblinded analysis. Five studies had a low risk of bias in this domain, with four (60, 61, 63, 70) due to a finalised pre-specified analysis plan being reported in the trial protocol and one (68) due to the analysis plan being followed and the outcome assessors being blinded.

Medical trials entail a comprehensive understanding of clinical ethics, with those involving children complicated by stricter standards than those involving adults (95). In addition, paediatric medical trials entail a careful balancing of benefit against risk and a consideration of the evolving stages of a child's development and an informed parental, often family-centred, decision making (96). These stricter ethical standards and requirements, together with fewer eligible participants, result in paediatric medical trials being more challenging and less frequent (95, 97). The outcome is that paediatric medical trials are often not supported by class I evidence, having a higher probability of bias and lower external validity. These issues correlate with the studies included in this systematic review and the overall higher risk of bias.

## 4. Discussion

DHIs are increasingly being used to prepare children and their parents for hospital procedures, aiming to reduce pre-operative anxiety and improve health outcomes. It is evidenced that well-prepared children are associated with reduced pre-operative anxiety and that DHIs can be an effective preparation method (13–16). This study aimed to use the TDF to evaluate the design and development of these paediatric preparation DHIs, determine whether a behavioural framework and co-production were used, and understand their impact on health outcomes. The four main findings of this review are discussed within the context of the modified TDF and the

1. health outcomes observed,
2. co-production and use of behaviour frameworks,
3. type of DHIs, and
4. timing and location of the DHIs used.

### 4.1. Health outcomes observed

All studies included in this review assessed child anxiety either as an emotion or as a feeling or behavioural response. Compared with children in the control group(s), 14 studies (82%) showed that children using the DHIs were associated with lower anxiety levels and the DHI had a positive impact, with this corresponding to the result of the effect size calculations where they could be calculated. This differed for three studies (17%), which showed anxiety levels were similar and the DHIs had no or little impact and effect. Given that higher pre-operative anxiety is a predictor of negative behavioural changes, the results for measures such as emergence delirium, induction behaviour, and induction compliance were mixed, although they were considered only in a small number of the included studies. For the three studies (60, 68, 71) that measured ED, only one found its occurrence lower in children prepared using the DHI. For the studies looking at induction behaviour (61, 65) and induction compliance (62, 63, 70), one study found improved induction behaviour and two found improved induction compliance in children using the DHI. Some of these health improvements are linked to higher scoring within the modified TDF and the first finding of this study.

The first finding is that paediatric preparation DHIs scoring higher against the modified TDF are more likely to be associated with reduced anxiety and reduced negative behavioural changes, as they will provide detailed information on the planned procedure and encompass information on coping with emotions, feelings, and anxiety (1, 13). Bray et al. (58), Stunden et al. (59), Wright et al. (62), Ryu et al. (70), and Fortier et al. (71) were the highest scoring studies against the modified TDF, having fully met 10 or more domains with 8 of these in common. The eight domains that were commonly met were knowledge, skills, behavioural regulation, environmental context and resources, belief about capabilities, beliefs about consequences, and reinforcement. This is attributed to the DHIs including (1) detailed information on the hospital environment, staff, equipment, and relevant procedure; (2) interactive elements such as games, quizzes, rewards, actions, or activities; and (3) breathing or coping exercises or modelling videos. The children using the DHIs in four of these studies were associated with lower anxiety levels (58, 62, 70, 71), lower occurrence of emergence delirium (71), and higher induction compliance (62, 70). This finding indicates that DHIs that incorporate these domains and are used as preparation interventions could be associated with reduced anxiety levels and other negative behavioural changes. An anomaly to this finding is the study by Stunden et al. (59). Stunden et al. (59) did not find any impact on child anxiety levels nor any difference in the key measure for head movement in their randomised control trial. This inconsistency is not likely to impact the first finding of this review for two reasons: (1) the trial was conducted with a simulated MRI and paid volunteers; and (2) all children reported no anxiety at baseline. This contrasts with the other four studies where the DHIs were used in preparation for real paediatric procedures, and varying levels of anxiety were reported at baseline. Nevertheless, the design of this DHI is considered relevant to the evaluation against the modified TDF. This finding cannot be extrapolated to all studies that reported positive health outcomes, given that the DHI scoring varied against the modified TDF. Despite this, the lack of meeting this finding can be attributed to either one or more of the remaining three findings, or insufficient information available in the study paper to make a judgement, thus resulting in a zero score.

### 4.2. Co-production and use of behaviour frameworks

The second finding is that preparation DHIs scoring higher against the modified TDF are more likely to have used co-production and a behavioural framework in their design and development. Aufegger et al. (21) stated that children and their parents prefer “easily digestible, non-medical explanations as to what to expect during the treatment process [together with information] on how to prepare”, whereas healthcare professionals suggest that information on policies, the hospital environment, staff roles and responsibilities, and patient flow timings are useful. In addition, Bray et al. (52) found that children valued coping strategy information as it enabled emotional self-regulation and provided more information about the procedure. Of the five DHIs scoring the highest against the modified TDF, three (58, 62, 71) fully met the co-production and behaviour framework domains. No information was provided in the papers by Stunden et al. (59) and Ryu et al. (70) to determine whether a behavioural framework was used, but both partially met the domain for co-production, having involved healthcare professionals in the DHI development. Fernandes et al. (67) used a behavioural framework and co-produced the DHI with healthcare professionals and children, with this DHI being the sixth highest scoring one. In the context of the findings from Aufegger et al. (21) and Bray et al. (52), the DHI in these studies all incorporated detailed information about the hospital environment, staff, equipment, and procedure, and the five highest scoring DHIs included interactive elements, coping strategies, or self-regulation feedback. The association between a higher modified TDF score and health outcomes is linked to the hypothesis in the primary objective of this review. Preparation DHIs that are co-designed and grounded in behavioural science can result in reduced pre-operative anxiety and improved health outcomes. However, further research is required to validate this finding.

The three higher scoring studies (58, 62, 71) that explicitly stated had used behavioural frameworks in designing and developing the DHIs were associated with lower levels of child anxiety, lower occurrence of emergence delirium, and higher induction compliance. In the context of theory-driven intervention design, execution, and reporting, behavioural frameworks such as the TDF offer an approach to understand and/or explain what influences the success of intervention implementation. Through understanding and explaining what influences will contribute to successful implementation, interventions aimed at changing behaviours can be designed and developed accordingly. Similarly, this study suggests that behavioural frameworks, such as the TDF, can be used to assess an intervention design and development in the context of implementation evaluation, thus supporting refinement of the intervention design.

### 4.3. Type of DHIs

The third finding is that the type of preparation DHI plays an important role in achieving a higher score against the modified TDF, with this being intrinsically linked to interactivity and rewards or achievements. In a previous qualitative study (17), children wanted preparation information that is easy to access, comprehensible, engaging, and child-friendly, as they believed that all of this will aid in the alleviation of their worries. This builds on the previous two findings, reiterating the value of interactive DHIs that incorporate games, quizzes, rewards, actions, or activities. Here again, the top six and the seventh highest scoring DHIs against the modified TDF were all interactive, being an educational multi-media App (58, 67), a VR-MRI App (59), a video App with games (64), a web-based program (62, 71), and a VR video game (70). An anomaly to this finding is the educational multi-media App by Huntington et al. (65) that scored very low against the modified TDF. However, this is due to the lack of information in the study paper to fully assess the DHI. The remaining DHIs were mostly non-interactive video tours, VR information, or web-based click-through presentations. Consequently, a correlation was further identified between the domain for optimism and the domains for skills, reinforcement, intentions, and goals. This was observed in three of the highest scoring DHIs by Stunden et al. (59), Ryu et al. (70), and Fortier et al. (71). All these scored fully or partially in the optimism, intention, and goal domains, and all fully scored in the reinforcement and skill domains. Stunden et al. (59) used interactive cues (skills) and real-time feedback on movement within the MRI stimulation (reinforcement and intention) to encourage stillness (goal), and when this was achieved, the child advanced to the next level (optimism). Ryu et al. (70) and Fortier et al. (71) used interactive games (skills) and breathing and coping exercises (reinforcement and intention) to advance through the steps or modules (goals), receiving health points and a completion certificate, respectively (optimism). Both these DHIs used interactive game elements to reinforce behaviour, such as chasing the germ monster after instructions in the recovery room and placing the anaesthesia mask on animals. These findings suggest that integrating interactive elements (skills and reinforcement) with feedback or rewards (intentions) could be used to drive certain actions or behavioural changes (goals) by creating the desire (optimism) to achieve the feedback or reward. Furthermore, for two of the studies, it is associated with improved outcomes. An irregularity to this correlation was the DHI by Campbell et al. (72). It failed to meet the reinforcement, intention, and optimism domains, but it partially met the skill and goal domains through the provision of a list of activities to prevent tooth decay at the end of the web-based presentation.

### 4.4. Timing and location of the DHI used

The fourth finding is that the timing and location of the preparation DHI lends itself to a higher score against the modified TDF. Three of the highest scoring DHIs, by Bray et al. (58), Wright et al. (62), and Fortier et al. (71), were provided for use at home by children and parents, as many times as they liked, between a week and 3 days before the procedure. These three DHIs were also associated with lower anxiety levels in the children using the DHI, and for two, lower occurrences of emergence delirium (71) and higher induction compliance, respectively (62). This suggests that the use of the DHI in the comfort of the child's own home, within a few or more days before the planned procedure, may contribute to reduced pre-operative anxiety and improved health outcomes.

### 4.5. Strengths and limitations

This study's strength is that it evaluates the design and development of DHIs used in preparing children for hospital procedures, correlating this against effectiveness in improving outcomes. Previous systematic reviews (6, 19, 20, 22, 23, 98–100) have predominately focused on the type of health interventions used and their effectiveness in improving health outcomes and/or reducing pre-operative anxiety, stress, and pain. In addition, some of these reviews included non-digital health interventions (98–100) and those used for distraction (6, 100). This study has specifically evaluated DHIs used for preparation.

There are limitations to this study. The first and second limitations relate to the search strategy and data extraction. While the search strategy was considered comprehensive, it was limited to papers in English published within the last 22 years, with the period being to ensure the relevance of the studies. When snowballing references of included papers and previous systematic reviews, a few papers published before the year 2000 may have been relevant for inclusion.

The third was the inability to conduct a meta-analysis because of the presence of heterogeneity across the included studies. Consequently, effect sizes were calculated, but not all studies reported the mean and standard deviation. It was, therefore, necessary to convert the median and interquartile ranges into a mean and standard deviation to then calculate the effect size. However, due to insufficient information to determine proximity to a normal distribution, the results may potentially be skewed. Some data reported in the studies were not amendable to calculating the effect size, and for these studies, the results were only narratively synthesised.

Finally, the level of information contained within some of the study papers to describe the DHIs was minimal, with supporting resources not found. This was a factor in the inability to draw meaningful conclusions against many of the modified TDF domains.

### 4.6. Quality of the studies

The quality of the studies was predominately moderate, with five studies having an overall high risk of bias. However, when considering the individual risk of bias in each of the five domains, it generally ranged from low to some concern, with most of the concerns linked either to an uncertainty of, or to a confirmed lack of, blinding of participants or those assessing the data, or to a lack of information in the papers to make a judgement. This was within the domains for “randomisation process” and “selection of reported results”, with the latter predominately linked to uncertainty on whether the analysis plan was finalised before results were assessed and the trial protocol not being readily available to verify, rather than the results being biased.

### 4.7. Implications for policy and future research

It is considered that this study is the first to use an adapted version of the TDF to assess the design and development of DHIs used to prepare children for hospital procedures. The four key findings from this study suggest that the TDF can be used to analyse the effects of preparation DHIs, and by using theory-driven behavioural science, their design can be redressed accordingly to improve health outcomes. While these findings contribute to this field of study, further research is required to validate the findings. Furthermore, research is required to understand the developmental costs of these preparation DHIs and whether they are cost-effective against the traditional form of pre-operative preparation.

## 5. Conclusion

The Theoretical Domains Framework is a validated tool designed to enable the evaluation of behaviour change and can be used to assess implementation issues, support intervention design, and analyse interventions. This study applied an adapted version of the Theoretical Domains Framework to assess the design and development of DHIs used to prepare children for hospital procedures.

The main findings from this assessment are that DHIs scoring highly against the modified TDF are

1. associated with positive health outcomes,
2. influenced by the use of co-production and behavioural science in their design and development,
3. interactive,
4. used a few days to a week in advance of the planned procedure within the comfort of the child's own home.

These four findings together are associated with reduced anxiety and reduced negative behavioural changes in the DHIs that scored the highest against the modified TDF. Furthermore, well-designed and developed DHIs that can be used in the child's own home and in advance of the planned procedure may be more cost-effective. This is in respect of the reduced staff time for on-the-day preparation and the potential longer-term reduced healthcare utilisation.

Paediatric preparation DHIs that are designed in the context of behavioural science and with co-development from healthcare professionals, children, and their parents are more likely to be associated with reduced pre-operative anxiety and have the potential for improving health outcomes. Furthermore, the use of paediatric preparation DHIs well in advance of planned invasive and non-invasive procedures may be more cost-effective than traditional preparation programmes such as Child Life Specialists or hospital tours that require staff time, resourcing, and planning around the child's procedure. By enabling pre-operative information to be provided digitally in the child's own home, these costs could be reduced. However, further research is required into the cost–benefit of this weighed against the developmental costs associated with the DHIs, particularly those that have shown to be more effective in reducing pre-operative anxiety.

## Data Availability

The original contributions presented in the study are included in the article/Supplementary Material, further inquiries can be directed to the corresponding author.

## References

[1] Kent Surrey Sussex Academic Health Science Network. *Evaluation report of the little journey mobile application*. Eastern AHSN. (2020). Available at: https://www.easternahsn.org/wp-content/uploads/2020/05/Little-Journey-evaluation.pdf (Accessed May 23, 2022).

[2] Yilmaz M Sezer H Gürler H Bekar M. Predictors of preoperative anxiety in surgical inpatients. J Clin Nurs. (2012) 21(7–8):956–64. 10.1111/j.1365-2702.2011.03799.x 21812848

[3] Gold JI Annick ET Lane AS Ho K Marty RT Espinoza JC. “Doc McStuffins: doctor for a day” virtual reality (DocVR) for pediatric preoperative anxiety and satisfaction: pediatric medical technology feasibility study. J Med Internet Res. (2021) 23(4):e25504. 10.2196/25504 33730687 PMC8094020

[4] Chorney JM Kain ZN. Behavioral analysis of children’s response to induction of anesthesia. Anesth Analg. (2009) 109(5):1434–40. 10.1213/ane.0b013e3181b412cf 19713262

[5] Kain ZN Mayes LC Caldwell-Andrews AA Karas DE McClain BC. Preoperative anxiety, postoperative pain, and behavioral recovery in young children undergoing surgery. Pediatrics. (2006) 118(2):651–8. 10.1542/peds.2005-2920 16882820

[6] Eijlers R Utens EMWJ Staals LM de Nijs PFA Berghmans JM Wijnen RMH Systematic review and meta-analysis of virtual reality in pediatrics: effects on pain and anxiety. Anesth Analg. (2019) 129(5):1344–53. 10.1213/ANE.0000000000004165 31136330 PMC6791566

[7] Messina M Molinaro F Meucci D Angotti R Giuntini L Cerchia E Preoperative distraction in children: hand-held videogames vs. clown therapy. Pediatr Med Chir. (2014) 36(5–6):204–6. 10.4081/pmc.2014.98 25669889

[8] Kain Z Mayes L O'Connor T Cicchetti D. Preoperative anxiety in children: predictors and outcomes. Arch Pediatr Adolesc Med. (1996) 150(12):1238–45. 10.1001/archpedi.1996.02170370016002 8953995

[9] Watson AT Visram A. Children's preoperative anxiety and postoperative behaviour. Paediatr Anaesth. (2003) 13(3):188–204. 10.1046/j.1460-9592.2003.00848.x 12641680

[10] Wang R Huang X Wang Y Akbari M. Non-pharmacologic approaches in preoperative anxiety, a comprehensive review. Front Public Health. (2022) 10:854673. 10.3389/fpubh.2022.854673 35480569 PMC9035831

[11] Chow CHT Van Lieshout RJ Schmidt LA Buckley N. Tablet-based intervention for reducing children's preoperative anxiety: a pilot study. J Dev Behav Pediatr. (2017) 38(6):409–16. 10.1097/dbp.0000000000000454 28661955

[12] Wright KD Stewart SH Finley GA Buffett-Jerrott SE. Prevention and intervention strategies to alleviate preoperative anxiety in children: a critical review. Behav Modif. (2007) 31(1):52–79. 10.1177/0145445506295055 17179531

[13] Ellerton M-L Merriam C. Preparing children and families psychologically for day surgery: an evaluation. J Adv Nurs. (1994) 19(6):1057–62. 10.1111/j.1365-2648.1994.tb01188.x 7930085

[14] Schwartz BH Albino JE. Effects of psychological preparation on children hospitalized for dental operations. J Pediatr. (1983) 102(4):634–8. 10.1016/S0022-3476(83)80211-X 6834205

[15] Brewer S Gleditsch SL Syblik D Tietjens ME Vacik HW. Pediatric anxiety: child life intervention in day surgery. J Pediatr Nurs. (2006) 21(1):13–22. 10.1016/j.pedn.2005.06.004 16428010

[16] Kain ZN Mayes LC Caramico LA. Preoperative preparation in children: a cross-sectional study. J Clin Anesth. (1996) 8(6):508–14. 10.1016/0952-8180(96)00115-8 8872693

[17] Bray L Appleton V Sharpe A. ‘If I knew what was going to happen, it wouldn’t worry me so much’: children’s, parents’ and health professionals’ perspectives on information for children undergoing a procedure. J Child Health Care. (2019) 23(4):626–38. 10.1177/1367493519870654 31431048

[18] Fortier MA Chorney JM Rony RYZ Perret-Karimi D Rinehart JB Camilon FS Children’s desire for perioperative information. Anesth Analg. (2009) 109(4):1085–90. 10.1213/ane.0b013e3181b1dd48 19762736 PMC2910260

[19] Chow CH Van Lieshout RJ Schmidt LA Dobson KG Buckley N. Systematic review: audiovisual interventions for reducing preoperative anxiety in children undergoing elective surgery. J Pediatr Psychol. (2016) 41(2):182–203. 10.1093/jpepsy/jsv094 26476281 PMC4884908

[20] Dai Y Livesley J. A mixed-method systematic review of the effectiveness and acceptability of preoperative psychological preparation programmes to reduce paediatric preoperative anxiety in elective surgery. J Adv Nurs. (2018) 74(9):2022–37. 10.1111/jan.13713 29754399

[21] Aufegger L Bùi KH Bicknell C Darzi A. Designing a paediatric hospital information tool with children, parents, and healthcare staff: a UX study. BMC Pediatr. (2020) 20(1):469. 10.1186/s12887-020-02361-w 33032549 PMC7542856

[22] Adegboro CO Choudhury A Asan O Kelly MM. Artificial intelligence to improve health outcomes in the NICU and PICU: a systematic review. Hosp Pediatr. (2021) 12(1):93–110. 10.1542/hpeds.2021-006094 34890453

[23] Cunningham A McPolin O Fallis R Coyle C Best P McKenna G. A systematic review of the use of virtual reality or dental smartphone applications as interventions for management of paediatric dental anxiety. BMC Oral Health. (2021) 21(1):244. 10.1186/s12903-021-01602-3 33962624 PMC8103574

[24] Cane J O’Connor D Michie S. Validation of the theoretical domains framework for use in behaviour change and implementation research. Implement Sci. (2012) 7(1):37. 10.1186/1748-5908-7-37 22530986 PMC3483008

[25] Michie S van Stralen MM West R. The behaviour change wheel: a new method for characterising and designing behaviour change interventions. Implement Sci. (2011) 6(1):42. 10.1186/1748-5908-6-42 21513547 PMC3096582

[26] Atkins L Francis J Islam R O’Connor D Patey A Ivers N A guide to using the theoretical domains framework of behaviour change to investigate implementation problems. Implement Sci. (2017) 12(1):77. 10.1186/s13012-017-0605-9 28637486 PMC5480145

[27] Corman HH Hornick EJ Kritchman M Terestman N. Emotional reactions of surgical patients to hospitalization, anesthesia and surgery. Am J Surg. (1958) 96(5):646–53. 10.1016/0002-9610(58)90466-5 13583329

[28] Yap JN-K. The effects of hospitalization and surgery on children: a critical review. J Appl Dev Psychol. (1988) 9(3):349–58. 10.1016/0193-3973(88)90035-4

[29] Vernon DTA Foley JM Sipowicz RR. The psychological responses of children to hospitalization and illness: A review of the literature. Springfield, IL: Charles C. Thomas (1965).

[30] Kain ZN Wang SM Mayes LC Caramico LA Hofstadter MB. Distress during the induction of anesthesia and postoperative behavioral outcomes. Anesth Analg. (1999) 88(5):1042–7. 10.1213/00000539-199905000-00013 10320165

[31] Munafò MR Stevenson J. Anxiety and surgical recovery: reinterpreting the literature. J Psychosom Res. (2001) 51(4):589–96. 10.1016/S0022-3999(01)00258-6 11595247

[32] Cao J Shi X Miao X Xu J. Effects of premedication of midazolam or clonidine on perioperative anxiety and pain in children. Biosci Trends. (2009) 3(3):115–8. Available at: https://www.biosciencetrends.com/article/217. 20103833

[33] O'Sullivan M Wong GK. Preinduction techniques to relieve anxiety in children undergoing general anaesthesia. Contin Educ Anaesth Crit Care Pain. (2013) 13:196–9. 10.1093/BJACEACCP/MKT014

[34] McCann ME Kain ZN. The management of preoperative anxiety in children: an update. Anesth Analg. (2001) 93(1):98–105. 10.1097/00000539-200107000-00022 11429348

[35] Heikal S Stuart G. Anxiolytic premedication for children. BJA Educ. (2020) 20(7):220–5. 10.1016/j.bjae.2020.02.006 33456954 PMC7807914

[36] Piira T Sugiura T Champion GD Donnelly N Cole AS. The role of parental presence in the context of children's medical procedures: a systematic review. Child Care Health Dev. (2005) 31(2):233–43. 10.1111/j.1365-2214.2004.00466.x 15715702

[37] Agbayani C-JG Fortier MA Kain ZN. Non-pharmacological methods of reducing perioperative anxiety in children. BJA Educ. (2020) 20(12):424–30. 10.1016/j.bjae.2020.08.003 33456927 PMC7807851

[38] Kain ZN Mayes LC Caramico LA Silver D Spieker M Nygren MM Parental presence during induction of anesthesia. A randomized controlled trial. Anesthesiology. (1996) 84(5):1060–7. 10.1097/00000542-199605000-00007 8623999

[39] Hannallah RS Rosales JK. Experience with parents’ presence during anaesthesia induction in children. Can J Anaesth. (1983) 30(3):286–9. 10.1007/BF03013809 6336550

[40] Cohen LL Bernard RS McClellan CB Piazza-Waggoner C Taylor BK MacLaren JE. Topical anesthesia versus distraction for infants’ immunization distress: evaluation with 6-month follow-up. Child Health Care. (2006) 35(2):103–21. 10.1207/s15326888chc3502_1

[41] Schechter NL Zempsky WT Cohen LL McGrath PJ McMurtry CM Bright NS. Pain reduction during pediatric immunizations: evidence-based review and recommendations. Pediatrics. (2007) 119(5):e1184–98. 10.1542/peds.2006-1107 17473085

[42] Sahiner NC Bal MD. The effects of three different distraction methods on pain and anxiety in children. J Child Health Care. (2016) 20(3):277–85. 10.1177/1367493515587062 26040282

[43] Karimi R Fadaiy Z Nikbakht Nasrabadi A Godarzi Z Mehran A. Effectiveness of orientation tour on children’s anxiety before elective surgeries. Jpn J Nurs Sci. (2014) 11(1):1110–15. 10.1111/j.1742-7924.2012.00223.x 24460597

[44] Spitzer P. The clown doctors. Aust Fam Physician. (2001) 30(1):12–6. 11211705

[45] Great Ormond Street Hospital for Children. *Play as a therapeutic tool*. Available at: https://www.gosh.nhs.uk/news/play-therapeutic-tool/#:∼:text=a%20therapeutic%20tool-,Play%20as%20a%20therapeutic%20tool,-12%20Sep%202019 (Accessed 22 May 2022).

[46] Koukourikos K Tzeha L Pantelidou P Tsaloglidou A. The importance of play during hospitalization of children. Mater Sociomed. (2015) 27(6):438–41. 10.5455/msm.2015.27.438-441 26889107 PMC4733554

[47] Kain Zeev N Caldwell-Andrews Alison A Mayes Linda C Weinberg Megan E Wang S-M MacLaren Jill E Family-centered preparation for surgery improves perioperative outcomes in children: a randomized controlled trial. Anesthesiology. (2007) 106(1):65–74. 10.1097/00000542-200701000-00013 17197846

[48] Rassin M Gutman Y Silner D. Developing a computer game to prepare children for surgery. AORN J. (2004) 80(6):1095–102. 10.1016/S0001-2092(06)60689-3 15641663

[49] Patel A Schieble T Davidson M Tran MCJ Schoenberg C Delphin E Distraction with a hand-held video game reduces pediatric preoperative anxiety. Pediat Anesth. (2006) 16(10):1019–27. 10.1111/j.1460-9592.2006.01914.x 16972829

[50] Kim J Chiesa N Raazi M Wright KD. A systematic review of technology-based preoperative preparation interventions for child and parent anxiety. Can J Anaesth. (2019) 66(8):966–86. 10.1007/s12630-019-01387-8 31098960

[51] O'Conner-Von S. Preparation of adolescents for outpatient surgery: using an internet program. AORN J. (2008) 87(2):374–98. 10.1016/j.aorn.2007.07.024 18262002

[52] Bray L Appleton V Sharpe A. The information needs of children having clinical procedures in hospital: will it hurt? Will I feel scared? What can I do to stay calm? Child Care Health Dev. (2019) 45(5):737–43. 10.1111/cch.12692 31163093 PMC6851850

[53] Alqudimat M Mesaroli G Lalloo C Stinson J Matava C. State of the art: immersive technologies for perioperative anxiety, acute, and chronic pain management in pediatric patients. Curr Anesthesiol Rep. (2021) 11(3):265–74. 10.1007/s40140-021-00472-3 34276254 PMC8277426

[54] Michie S Johnston M Abraham C Lawton R Parker D Walker A Making psychological theory useful for implementing evidence based practice: a consensus approach. Qual Saf Health Care. (2005) 14(1):26–33. 10.1136/qshc.2004.011155 15692000 PMC1743963

[55] Oxford Centre for Evidence-Based Medicine. *Asking focused questions*. Available at: https://www.cebm.ox.ac.uk/resources/ebm-tools/asking-focused-questions (Accessed August 10, 2022).

[56] Thomas J Kneale D McKenzie JE Brennan SE Bhaumik S. Chapter 2: determining the scope of the review and the questions it will address. In: Higgins JPT Thomas J Chandler J Cumpston M Li T Page MJ Welch VA, editors. Cochrane handbook for systematic reviews of interventions version 63 (updated February 2022). Cochrane (2022). Available at: www.training.cochrane.org/handbook (Accessed 18 March 2022).

[57] Veritas Health Innovation. Covidence systematic review software. Melbourne (2022). Available at: www.covidence.org (Accessed 10 August 2021).

[58] Bray L Sharpe A Gichuru P Fortune P-M Blake L Appleton V. The acceptability and impact of the Xploro digital therapeutic platform to inform and prepare children for planned procedures in a hospital: before and after evaluation study. J Med Internet Res. (2020) 22(8):e17367. 10.2196/17367 32780025 PMC7448172

[59] Stunden C Stratton K Zakani S Jacob J. Comparing a virtual reality–based simulation app (VR-MRI) with a standard preparatory manual and child life program for improving success and reducing anxiety during pediatric medical imaging: randomized clinical trial. J Med Internet Res. (2021) 23(9):e22942. 10.2196/22942 34550072 PMC8495586

[60] Ryu JH Oh AY Yoo HJ Kim JH Park JW Han SH. The effect of an immersive virtual reality tour of the operating theater on emergence delirium in children undergoing general anesthesia: a randomized controlled trial. Paediatr Anaesth. (2019) 29(1):98–105. 10.1111/pan.13535 30365231

[61] Ryu JH Park SJ Park JW Kim JW Yoo HJ Kim TW Randomized clinical trial of immersive virtual reality tour of the operating theatre in children before anaesthesia. Br J Surg. (2017) 104(12):1628–33. 10.1002/bjs.10684 28975600

[62] Wright KD Kim J Ratcliffe CRD Walker KL Sharpe D Wilson S Pilot examination of the efficacy of the internet-delivered, preoperative, preparation program (I-PPP). J Clin Psychol Med Settings. (2021) 28(3):627–36. 10.1007/s10880-020-09754-0 33247796

[63] Park JW Nahm FS Kim JH Jeon YT Ryu JH Han SH. The effect of mirroring display of virtual reality tour of the operating theatre on preoperative anxiety: a randomized controlled trial. IEEE J Biomed Health Inform. (2019) 23(6):2655–60. 10.1109/JBHI.2019.2892485 30640637

[64] Wantanakorn P Harintajinda S Chuthapisith J Anurathapan U Rattanatamrong P. A new mobile application to reduce anxiety in pediatric patients before bone marrow aspiration procedures. Hosp Pediatr. (2018) 8(10):643–50. 10.1542/hpeds.2018-0073 30213798

[65] Huntington C Liossi C Donaldson AN Newton JT Reynolds PA Alharatani R On-line preparatory information for children and their families undergoing dental extractions under general anesthesia: a phase III randomized controlled trial. Paediatr Anaesth. (2018) 28(2):157–66. 10.1111/pan.13307 29280239 PMC5814894

[66] Hatipoglu Z Gulec E Lafli D Ozcengiz D. Effects of auditory and audiovisual presentations on anxiety and behavioral changes in children undergoing elective surgery. Niger J Clin Pract. (2018) 21(6):788–94. 10.4103/njcp.njcp_227_17 29888729

[67] Fernandes S Arriaga P Esteves F. Using an educational multimedia application to prepare children for outpatient surgeries. Health Commun. (2015) 30(12):1190–200. 10.1080/10410236.2014.896446 25144403

[68] Eijlers R Dierckx B Staals LM Berghmans JM van der Schroeff MP Strabbing EM Virtual reality exposure before elective day care surgery to reduce anxiety and pain in children: a randomised controlled trial. Eur J Anaesthesiol. (2019) 36(10):728–37. 10.1097/EJA.0000000000001059 31356373 PMC6738544

[69] Liguori S Stacchini M Ciofi D Olivini N Bisogni S Festini F. Effectiveness of an app for reducing preoperative anxiety in children: a randomized clinical trial. JAMA Pediatr. (2016) 170(8):e160533. 10.1001/jamapediatrics.2016.0533 27294708

[70] Ryu JH Park JW Nahm FS Jeon YT Oh AY Lee HJ The effect of gamification through a virtual reality on preoperative anxiety in pediatric patients undergoing general anesthesia: a prospective, randomized, and controlled trial. J Clin Med. (2018) 7(9):284. 10.3390/jcm7090284 30227602 PMC6162739

[71] Fortier MA Bunzli E Walthall J Olshansky E Saadat H Santistevan R Web-based tailored intervention for preparation of parents and children for outpatient surgery (WebTIPS): formative evaluation and randomized controlled trial. Anesth Analg. (2015) 120(4):915–22. 10.1213/ANE.0000000000000632 25790213 PMC4367120

[72] Campbell C Hosey M Creanor S. Facilitating coping behaviour in children prior to dental general anaesthesia: a randomised controlled trial. Paediatr Anaesth. (2005) 15:831–8. 10.1111/j.1460-9592.2004.01565.x 16176310

[73] Wakimizu R Kamagata S Kuwabara T Kamibeppu K. A randomized controlled trial of an at-home preparation programme for Japanese preschool children: effects on children's and caregivers’ anxiety associated with surgery. J Eval Clin Pract. (2009) 15(2):393–401. 10.1111/j.1365-2753.2008.01082.x 19335503

[74] Dehghan F Jalali R Bashiri H. The effect of virtual reality technology on preoperative anxiety in children: a Solomon four-group randomized clinical trial. Perioper Med. (2019) 8(5):1–7. 10.1186/s13741-019-0116-0 PMC6549331 31171963

[75] McHugh ML. Interrater reliability: the kappa statistic. Biochem Med (Zagreb). (2012) 22(3):276–82. 10.11613/BM.2012.031 23092060 PMC3900052

[76] Page MJ McKenzie JE Bossuyt PM Boutron I Hoffmann TC Mulrow CD The PRISMA 2020 statement: an updated guideline for reporting systematic reviews. Br Med J. (2021) 372:n71. 10.1136/bmj.n71 33782057 PMC8005924

[77] Cochrane Effective Practice and Organisation of Care (EPOC). *Data collection form*. Available at: https://epoc.cochrane.org/sites/epoc.cochrane.org/files/public/uploads/Resources-for-authors2017/good_practice_data_extraction_form.doc (Accessed 10 June 2022).

[78] Sterne J Savović J Page M Elbers R Blencowe N Boutron I Rob 2: a revised tool for assessing risk of bias in randomised trials. Br Med J. (2019) 366:l4898. Available at: https://www.riskofbias.info/welcome/rob-2-0-tool (Accessed June 21, 2022). 10.1136/bmj.l4898 31462531

[79] Critical Appraisal Skills Programme. *CASP checklists*. Available at: https://casp-uk.net/casp-tools-checklists/ (Accessed June 21, 2022).

[80] McKenzie J Brennan S. Chapter 12: synthesizing and presenting findings using other methods. In: Higgins J Thomas J Chandler J Cumpston M Li T Page M, editors. Cochrane handbook for systematic reviews of interventions version 63 (updated February 2022). Cochrane (2022). Available at: https://training.cochrane.org/handbook (Accessed April 09, 2022).

[81] Brown M Richardson M. Understanding and synthesizing numerical data from intervention studies. In: Boland A Cherry MG Dickson R, editors. Doing a systematic review: a student's guide. 2nd ed. Los Angeles, CA: SAGE (2017). p. 131–54.

[82] Smalley KR Aufegger L Flott K Mayer EK Darzi A. Can self-management programmes change healthcare utilisation in COPD?: a systematic review and framework analysis. Patient Educ Couns. (2021) 104(1):50–63. 10.1016/j.pec.2020.08.015 32912809 PMC7762718

[83] Campbell M McKenzie JE Sowden A Katikireddi SV Brennan SE Ellis S Synthesis without meta-analysis (SWiM) in systematic reviews: reporting guideline. Br Med J. (2020) 368:l6890. 10.1136/bmj.l6890 31948937 PMC7190266

[84] Social Science Statistics. *Effect size calculator for t-test*. Available at: https://www.socscistatistics.com/effectsize/default3.aspx (Accessed September 5, 2022).

[85] Wilson DB. *Practical meta-analysis effect size calculator [online calculator]*. Available from: https://www.campbellcollaboration.org/escalc/html/EffectSizeCalculator-Home.php (Accessed September 1, 2022).

[86] Estimating the sample mean and standard deviation from the sample size m, range and/or interquartile range. Available from: https://www.math.hkbu.edu.hk/∼tongt/papers/median2mean.html (Accessed September 5, 2022).

[87] Wan X Wang W Liu J Tong T. Estimating the sample mean and standard deviation from the sample size, median, range and/or interquartile range. BMC Med Res Methodol. (2014) 14(1):135. 10.1186/1471-2288-14-135 25524443 PMC4383202

[88] Luo D Wan X Liu J Tong T. Optimally estimating the sample mean from the sample size, median, mid-range, and/or mid-quartile range. Stat Methods Med Res. (2018) 27(6):1785–805. 10.1177/0962280216669183 27683581

[89] Shi J Luo D Weng H Zeng X-T Lin L Chu H Optimally estimating the sample standard deviation from the five-number summary. Res Synth Methods. (2020) 11(5):641–54. 10.1002/jrsm.1429 32562361

[90] Shi J Lou D Wan X Liu Y Liu J Bian Z *Detecting the skewness of data from the sample size and the five-number summary*. (2020). p. 1–36. Available at: https://arxiv.org/pdf/2010.05749.pdf (Accessed September 1, 2022).

[91] Drahota A Beller E. *RevMan calculator*. Available at: https://training.cochrane.org/resource/revman-calculator (Accessed September 12, 2022).

[92] JAMA Pediatrics. “*Clickamico”: a video to reduce preoperative anxiety in children*. Available from: https://www.facebook.com/watch/?v=10155045680624863 (Accessed July 25, 2022).

[93] Kain ZN Fortier MA Chorney JM Mayes L. Web-based tailored intervention for preparation of parents and children for outpatient surgery (WebTIPS): development. Anesth Analg. (2015) 120(4):905–14. 10.1213/ane.0000000000000610 25790212 PMC4367194

[94] RoB2 Development Group, Higgins JP Savović J Page MJ Sterne JA, editors. Revised cochrane risk-of-bias tool for randomized trials (RoB 2). Cochrane (2019). Available at: https://drive.google.com/file/d/19R9savfPdCHC8XLz2iiMvL_71lPJERWK/view (Accessed 21 June 2021).

[95] Balak N Sandvik U Honeybul S. Paediatric neurosurgery. In: Honeybul S, editor. Ethics in neurosurgical practice. Cambridge: Cambridge University Press (2020). p. 154–67.

[96] Sammons H Starkey E. Ethical issues of clinical trials in children. Paediatr Child Health (Oxford). (2016) 26(3):95–8. 10.1016/j.paed.2015.09.003

[97] Caldwell PHY Murphy SB Butow PN Craig JC. Clinical trials in children. Lancet. (2004) 364(9436):803–11. 10.1016/S0140-6736(04)16942-0 15337409

[98] Kerimaa H Ruotsalainen H Kyngas H Miettunen J Polkki T. Effectiveness of interventions used to prepare preschool children and their parents for day surgery: a systematic review and meta-analysis of randomised controlled trials. J Clin Nurs. (2021) 0(0):1–18. 10.1111/jocn.16156 34870345

[99] Levin M Seligman NL Hardy H Mohajeri S Maclean JA. Pediatric pre-tonsillectomy education programs: a systematic review. Int J Pediatr Otorhinolaryngol. (2019) 122:6–11. 10.1016/j.ijporl.2019.03.024 30921630

[100] Mathias EG Pai MS Guddattu V Bramhagen AC. Non-pharmacological interventions to reduce anxiety among children undergoing surgery: a systematic review. J Child Health Care. (2022):0(0). 10.1177/13674935211062336 35098734

