# Supplementary material for: Systematic review of the development and effectiveness of digital health information interventions, compared with usual care, in supporting patient preparation for paediatric hospital care, and the impact on their health outcomes
Source: Front Health Serv. 2023 Apr 6;3:1103624. doi: 10.3389/frhs.2023.1103624 (PMC10117991; doi:10.3389/frhs.2023.1103624)
Supplement: Supplementary file 1 [file Datasheet1.zip › Supplementary files/Appendix A.DOCX]

# Appendix A

# Table 1. Inclusion and exclusion criteria using PICOS.

|  | **Population** | **Intervention** | **Comparison** | **Outcome** | **Study type** |
| --- | --- | --- | --- | --- | --- |
| **Include** | Children aged 1 to and including 14 years and their parents/ caregivers | Digital education or preparation programmes.  Digital interventions are used as preparation tools to inform children and their parents/ caregivers about the medical procedure, including the hospital environment, equipment, and staff.  Digital interventions included:   - Audio-visual tools - Virtual reality - Smartphone or tablet or computer applications - Computer or video games - Website or online programmes and/or games. | Usual care (no intervention)  Head-to-head comparison of two information interventions in the same population | Changes in cognitive outcomes of children and parents/ caregivers such as stress, anxiety, confidence etc.  AND/OR  Changes in behavioural outcomes of children  AND/OR  Any impact on clinical status (length of stay, recovery rates) and/or healthcare utilisation (GP visits, medication use or readmission) | Randomised controlled trials  Non-randomised controlled trails  Quasi-experimental designs |
| **Exclude** | Children aged under 1 and 15 years and over  Interventions only directed at parents/ caregivers, or healthcare professionals  Children with cognitive impairments | Non-digitalised intervention (pictures, images, pamphlets, and brochures).  Digitalised self-management applications for chronic diseases  Digital distraction tools or play, including the use of relaxation techniques only to decrease anxiety and stress | Research into the comparison between two modes of interventions, such as distraction versus information intervention. | Studies focusing on parents/ carers and/or healthcare staff only | All other study types, including:   - systematic or scoping reviews - mini reviews - pilot studies - cohort studies - case-control studies - case studies - feasibility studies - commentary - ethnography - qualitative results only - no results - protocols - conference proceeding |
