# Supplementary material for: Systematic review of the development and effectiveness of digital health information interventions, compared with usual care, in supporting patient preparation for paediatric hospital care, and the impact on their health outcomes
Source: Front Health Serv. 2023 Apr 6;3:1103624. doi: 10.3389/frhs.2023.1103624 (PMC10117991; doi:10.3389/frhs.2023.1103624)
Supplement: Supplementary file 1 [file Datasheet1.zip › Supplementary files/Appendix B.DOCX]

# Appendix B: Search syntax for each database

| **Database** | **Search terms** |
| --- | --- |
| MEDLINE | 1. child*.mp. 2. exp Child/ 3. adolescent*.mp. 4. Adolescent/ 5. p?ediatric*.mp. 6. youth*.mp. 7. kids.mp. 8. young person.mp. 9. (young adj2 person).mp. 10. young people.mp. 11. (young adj2 people).mp. 12. young.mp. 13. (young adj2 child*).mp. 14. 1 or 2 or 3 or 4 or 5 or 6 or 7 or 8 or 9 or 10 or 11 or 12 or 13 15. healthcare utili?ation.mp. 16. (healthcare adj2 utili?ation).mp. 17. "patient acceptance of healthcare".mp. 18. "Patient Acceptance of Health Care".mp. 19. "Patient Acceptance of Health Care"/ 20. "patient acceptance".mp. 21. length of stay.mp. 22. "Length of Stay"/ 23. recover* rate*.mp. 24. (recover* adj2 rate*).mp. 25. health literac*.mp. 26. exp Health Literacy/ 27. Stress, Psychological/ 28. psychol* stress.mp. 29. anxiet*.mp. 30. Anxiety/ 31. behav* change*.mp. 32. (behav* adj1 change*).mp. 33. worr*.mp. 34. health outcome*.mp. 35. 15 or 16 or 17 or 18 or 19 or 20 or 21 or 22 or 23 or 24 or 25 or 26 or 27 or 28 or 29 or 30 or 31 or 32 or 33 or 34 36. preoperative.mp. 37. pre-operative.mp. 38. preadmission.mp. 39. pre-admission.mp. 40. prepar*.mp. 41. inpatient.mp. 42. 36 or 37 or 38 or 39 or 40 or 41 43. digit* education program*.mp. 44. digit* educat*.mp. 45. digit* education.mp. 46. (digit* adj1 educat*).mp. 47. (digit* adj2 educat*).mp. 48. digit* preparation*.mp. 49. digit* prepar*.mp. 50. digit* adj2 prepar*).mp. 51. 43 or 44 or 45 or 46 or 47 or 48 or 49 or 50 52. virtual realit*.mp. 53. exp virtual reality/ 54. ehealth.mp. 55. e-health.mp. 56. e?health.mp. 57. mhealth.mp. 58. m-health.mp. 59. m?health.mp. 60. "mobile health".mp. 61. apps.mp. 62. internet.mp. 63. internet/ or internet-based intervention/ 64. online.mp. 65. digit*.mp. 66. mobile applications.mp. 67. exp Mobile Applications/ 68. website*.mp. 69. web.mp. 70. 52 or 53 or 54 or 55 or 56 or 57 or 58 or 59 or 60 or 61 or 62 or 63 or 64 or 65 or 66 or 67 or 68 or 69 71. computer game*.mp.   Game terms   1. video game*.mp. 2. Video Games/ 3. 43 or 44 or 45 or 46 or 47 or 48 or 49 or 50 or 52 or 53 or 54 or 55 or 56 or 57 or 58 or 59 or 60 or 61 or 62 or 63 or 64 or 65 or 66 or 67 or 68 or 69 or 71 or 72 or 73 (with game terms) 4. 51 or 70 (without game terms) 5. 14 and 35 and 42 and 75 (without game terms) 6. 14 and 35 and 42 and 74 (with game terms) 7. limit 76 to yr="2000 -Current" (without game terms) 8. limit 77 to yr="2000 -Current" (with game terms) 9. limit 79 to English 10. limit 81 to (("preschool child (2 to 5 years)" or "child (6 to 12 years)" or "adolescent (13 to 18 years)") and English) |
| EMBASE | 1. child*.mp. 2. exp Child/ 3. adolescent*.mp. 4. exp adolescent/ 5. p?ediatric*.mp. 6. youth*.mp. 7. kids.mp. 8. young person.mp. 9. (young adj2 person).mp. 10. young people.mp. 11. (young adj2 people).mp. 12. young.mp. 13. (young adj2 child*).mp. 14. child patient.mp. 15. 1 or 2 or 3 or 4 or 5 or 6 or 7 or 8 or 9 or 10 or 11 or 12 or 13 or 14 16. healthcare utili?ation.mp. 17. (healthcare adj2 utili?ation).mp. 18. "patient acceptance of healthcare".mp. 19. "patient acceptance of health care".mp. 20. exp patient attitude/ 21. patient acceptance.mp. 22. length of stay.mp. 23. "length of stay"/ 24. recover* rate*.mp. 25. (recover* adj2 rate*).mp. 26. health literac*.mp. 27. exp health literacy/ 28. exp physiological stress/ 29. exp mental stress/ 30. psychol* stress.mp. 31. anxiet*.mp. 32. Anxiety/ 33. behav* change*.mp. 34. (behav* adj1 change*).mp. 35. worr*.mp. 36. health outcome*.mp. 37. 16 or 17 or 18 or 19 or 20 or 21 or 22 or 23 or 24 or 25 or 26 or 27 or 28 or 29 or 30 or 31 or 32 or 33 or 34 or 35 or 36 38. preoperative.mp. 39. pre-operative.mp. 40. pre?operative.mp. 41. preadmission.mp. 42. pre-admission.mp. 43. prepar*.mp. 44. 38 or 39 or 40 or 41 or 42 or 43 45. digit* education program*.mp. 46. digit* educat*.mp. 47. digit* education.mp. 48. (digit* adj1 educat*).mp. 49. (digit* adj2 educat*).mp. 50. digit* preparation*.mp. 51. digit* prepar*.mp. 52. digit* adj2 prepar*).mp. 53. 45 or 46 or 47 or 48 or 49 or 50 or 51 or 52 54. virtual realit*.mp. 55. exp virtual reality/ 56. ehealth.mp. 57. e-health.mp. 58. e?health.mp. 59. mhealth.mp. 60. m-health.mp. 61. m?health.mp. 62. "mobile health".mp. 63. apps.mp. 64. internet.mp. 65. internet/ or internet-based intervention/ 66. online.mp. 67. digit*.mp. 68. mobile application*.mp. 69. exp mobile application/ 70. website*.mp. 71. web.mp. 72. 54 or 55 or 56 or 57 or 58 or 59 or 60 or 61 or 62 or 63 or 64 or 65 or 66 or 67 or 68 or 69 or 70 or 71 73. computer game*.mp.   Game terms   1. video game*.mp. 2. video game/ 3. 45 or 46 or 47 or 48 or 49 or 50 or 51 or 52 or 54 or 55 or 56 or 57 or 58 or 59 or 60 or 61 or 62 or 63 or 64 or 65 or 66 or 67 or 68 or 69 or 70 or 71 or 73 or 74 or 75 (with game terms) 4. 53 or 72 (without game terms) 5. 15 and 37 and 44 and 77 (without game terms) 6. 15 and 37 and 44 and 76 (with game terms) 7. limit 78 to yr="2000 -Current" (without game terms) 8. limit 79 to yr="2000 -Current" (with game terms) 9. limit 81 to English 10. limit 82 to (English and (child <unspecified age> or preschool child <1 to 6 years> or school child <7 to 12 years> or adolescent <13 to 17 years>)) |
| HMIC | 1. child*.mp. 2. exp child/ 3. adolescent*.mp. 4. exp adolescent/ 5. p?ediatric*.mp. 6. youth*.mp. 7. kids.mp. 8. young person.mp. 9. (young adj2 person).mp. 10. young people.mp. 11. (young adj2 people).mp. 12. young.mp. 13. (young adj2 child*).mp. 14. child patient.mp. 15. 1 or 2 or 3 or 4 or 5 or 6 or 7 or 8 or 9 or 10 or 11 or 12 or 13 or 14 16. healthcare utili?ation.mp. 17. (healthcare adj2 utili?ation).mp. 18. "patient acceptance of healthcare".mp. 19. "Patient Acceptance of health care".mp. 20. exp patient attitude/ 21. patient acceptance.mp. 22. length of stay.mp. 23. "length of stay"/ 24. recover* rate*.mp. 25. (recover* adj2 rate*).mp. 26. health literac*.mp. 27. exp health literacy/ 28. exp physiological stress/ 29. exp mental stress/ 30. psychol* stress.mp. 31. anxiet*.mp. 32. anxiety/ 33. behav* change*.mp. 34. (behav* adj1 change*).mp. 35. worr*.mp. 36. health outcome*.mp. 37. 16 or 17 or 18 or 19 or 20 or 21 or 22 or 23 or 24 or 25 or 26 or 27 or 28 or 29 or 30 or 31 or 32 or 33 or 34 or 35 or 36 38. preoperative.mp. 39. pre-operative.mp. 40. pre?operative.mp. 41. preadmission.mp. 42. pre-admission.mp. 43. prepar*.mp. 44. 38 or 39 or 40 or 41 or 42 or 43 45. digit* education program*.mp. 46. digit* educat*.mp. 47. digit* education.mp. 48. (digit* adj1 educat*).mp. 49. (digit* adj2 educat*).mp. 50. digit* preparation*.mp. 51. digit* prepar*.mp. 52. digit* adj2 prepar*).mp. 53. 45 or 46 or 47 or 48 or 49 or 50 or 51 or 52 54. virtual realit*.mp. 55. exp virtual reality/ 56. ehealth.mp. 57. e-health.mp. 58. e?health.mp. 59. mhealth.mp. 60. m-health.mp. 61. m?health.mp. 62. "mobile health".mp. 63. apps.mp. 64. internet.mp. 65. internet/ or internet-based intervention/ 66. online.mp. 67. digit*.mp. 68. mobile application.mp. 69. exp mobile Application/ 70. website*.mp. 71. web.mp. 72. 54 or 55 or 56 or 57 or 58 or 59 or 60 or 61 or 62 or 63 or 64 or 65 or 66 or 67 or 68 or 69 or 70 or 71 73. computer game*.mp.   Game terms   1. video game*.mp. 2. video game/ 3. 45 or 46 or 47 or 48 or 49 or 50 or 51 or 52 or 54 or 55 or 56 or 57 or 58 or 59 or 60 or 61 or 62 or 63 or 64 or 65 or 66 or 67 or 68 or 69 or 70 or 71 or 73 or 74 or 75 (with game terms) 4. 53 or 72 (without game terms) 5. 15 and 37 and 44 and 77 (without game terms) 6. 15 and 37 and 44 and 76 (with game terms) 7. limit 78 to yr="2000 -Current" (without game terms) 8. limit 79 to yr="2000 -Current" (with game terms) 9. limit 81 to English |
| PsycINFO | 1. child*.mp. 2. exp child/ 3. adolescent*.mp. 4. exp adolescent/ 5. p?ediatric*.mp. 6. youth*.mp. 7. kids.mp. 8. young person.mp. 9. (young adj2 person).mp. 10. young people.mp. 11. (young adj2 people).mp. 12. young.mp. 13. (young adj2 child*).mp. 14. child patient.mp. 15. 1 or 2 or 3 or 4 or 5 or 6 or 7 or 8 or 9 or 10 or 11 or 12 or 13 or 14 16. healthcare utili?ation.mp. 17. (healthcare adj2 utili?ation).mp. 18. "patient acceptance of healthcare".mp. 19. "patient acceptance of health care".mp. 20. exp patient attitude/ 21. patient acceptance.mp. 22. length of stay.mp. 23. "length of stay"/ 24. recover* rate*.mp. 25. (recover* adj2 rate*).mp. 26. health literac*.mp. 27. exp health literacy/ 28. exp physiological stress/ 29. exp mental stress/ 30. psychol* stress.mp. 31. anxiet*.mp. 32. Anxiety/ 33. behav* change*.mp. 34. (behav* adj1 change*).mp. 35. worr*.mp. 36. health outcome*.mp. 37. 16 or 17 or 18 or 19 or 20 or 21 or 22 or 23 or 24 or 25 or 26 or 27 or 28 or 29 or 30 or 31 or 32 or 33 or 34 or 35 or 36 38. preoperative.mp. 39. pre-operative.mp. 40. pre?operative.mp. 41. preadmission.mp. 42. pre-admission.mp. 43. prepar*.mp. 44. 38 or 39 or 40 or 41 or 42 or 43 45. digit* education program*.mp. 46. digit* educat*.mp. 47. digit* education.mp. 48. (digit* adj1 educat*).mp. 49. (digit* adj2 educat*).mp. 50. digit* preparation*.mp. 51. digit* prepar*.mp. 52. digit* adj2 prepar*).mp. 53. 45 or 46 or 47 or 48 or 49 or 50 or 51 or 52 54. virtual realit*.mp. 55. exp virtual reality/ 56. ehealth.mp. 57. e-health.mp. 58. e?health.mp. 59. mhealth.mp. 60. m-health.mp. 61. m?health.mp. 62. "mobile health".mp. 63. apps.mp. 64. internet.mp. 65. internet/ or internet-based intervention/ 66. online.mp. 67. digit*.mp. 68. mobile application*.mp. 69. exp mobile application/ 70. website*.mp. 71. web.mp. 72. 54 or 55 or 56 or 57 or 58 or 59 or 60 or 61 or 62 or 63 or 64 or 65 or 66 or 67 or 68 or 69 or 70 or 71 73. computer game*.mp.   Game terms   1. video game*.mp. 2. video game/ 3. 45 or 46 or 47 or 48 or 49 or 50 or 51 or 52 or 54 or 55 or 56 or 57 or 58 or 59 or 60 or 61 or 62 or 63 or 64 or 65 or 66 or 67 or 68 or 69 or 70 or 71 or 73 or 74 or 75 (with game terms) 4. 53 or 72 (without game terms) 5. 15 and 37 and 44 and 77 (without game terms) 6. 15 and 37 and 44 and 76 (with game terms) 7. limit 78 to yr="2000 -Current" (without game terms) 8. limit 79 to yr="2000 -Current" (with game terms) 9. limit 81 to English 10. limit 82 to ((childhood <birth to 12 years> or adolescence <13 to 17 years>) and English) |
