# Supplementary material for: Systematic review of the development and effectiveness of digital health information interventions, compared with usual care, in supporting patient preparation for paediatric hospital care, and the impact on their health outcomes
Source: Front Health Serv. 2023 Apr 6;3:1103624. doi: 10.3389/frhs.2023.1103624 (PMC10117991; doi:10.3389/frhs.2023.1103624)
Supplement: Supplementary file 1 [file Datasheet1.zip › Supplementary files/Appendix C.DOCX]

# Appendix C: Full-Text Screening Selection Process Questions

1. Is the article published in English?
2. If yes, proceed to 2.
3. If no, exclude with code LANGUAGE.
4. Does the article describe an original, empirical study?
5. If yes, proceed to 3.
6. If commentary, editorial, or theoretical work, exclude with code STUDY DESIGN.
7. Is the article’s study design a randomised control trial (RCT) or non-randomised control trial (Non-RCT) or a quasi-experimental design?
8. If it is an RCT, proceed to 4.
9. If it is a Non-RCT or quasi-experimental design then proceed to 5.
10. If no, exclude with code STUDY DESIGN.
11. If the study is an RCT, is the RCT a pilot or feasibility study?
12. If yes, exclude with code STUDY DESIGN.
13. If no, proceed to 5.
14. Does the article articulate a clear control group/comparator?
15. If yes, proceed to 6.
16. If no, exclude with code COMPARATOR.
17. Does the article pertain to patients aged up to 14 years, and their parents or caregivers?
18. If yes, proceed to 7.
19. If no, exclude with code PARTICIPANT.
20. Does the article pertain to patients who reside in an institutional setting?
21. If yes, exclude with code PARTICIPANT.
22. If no, proceed to 8.
23. Does the article pertain to patients with a primary diagnosis of mental illness, learning disability, or physical disability?
24. If yes, exclude with code PARTICIPANT.
25. If no, proceed to 9.
26. Does the article evaluate the use of a digital intervention to educate, prepare and inform the patient (and their parent or caregiver) about treatment and/or a procedure, providing information and/or education about the hospital environment, treatment, or procedure to be undertaken, medical equipment, or healthcare staff roles and responsibilities?
27. If yes, proceed to 10.
28. If no, exclude with code INTERVENTION.
29. Does the article evaluate the use of digital interventions' effect on emotional (cognitive) and behavioural responses, AND/OR impact on healthcare utilisation AND/OR clinical status?
30. If yes, proceed to 11.
31. If no, exclude with code INTERVENTION.
32. Is the digital intervention aimed at parents or caregivers and/or healthcare staff only?
33. If yes, exclude with code INTERVENTION.
34. If no, proceed to 12.
35. Does the study report an outcome related to ‘change in healthcare utilisation’, such as hospital admissions, readmissions, GP visits, or similar OR a change related to ‘clinical status’, such as length of stay or recovery rate, AND/OR a change in cognition AND/OR behaviour?
36. If yes, include and complete the data extraction form.
37. If no, exclude with code OUTCOME.
