# Supplementary material for: Systematic review of the development and effectiveness of digital health information interventions, compared with usual care, in supporting patient preparation for paediatric hospital care, and the impact on their health outcomes
Source: Front Health Serv. 2023 Apr 6;3:1103624. doi: 10.3389/frhs.2023.1103624 (PMC10117991; doi:10.3389/frhs.2023.1103624)
Supplement: Supplementary file 1 [file Datasheet1.zip › Supplementary files/Appendix D.DOCX]

# Appendix D

# Table 2. Data extraction fields.

| Data extraction table | Fields |
| --- | --- |
| Study characteristics | Study ID, author, publication year, title, study funding source, conflicts of interest, ethics approval, the aim of the study, study design, country, start and end dates, inclusion criteria, exclusion criteria, setting, type of DHI and details, intervention to surgery/procedure time, length of intervention, and comparator (usual care or control). |
| Participant characteristics | Study ID, author, publication year, population, method of recruitment, total number of participants, control/usual care group (age, sample size, percentage and retention rate), intervention group (age, sample size, percentage and retention rate), child sex (male sample size and percentage, female sample size and percentage), child ethnicity, surgery or procedure, parent/caregiver age, additional information. |
| Measurements and outcomes | Study ID, author, publication year, type of outcome measures, the timing of measures, analysis information, measure details, baseline data (mean and SD or median and interquartile range, P value, other data, participants), outcomes data (mean and SD or median and interquartile range, P value, other data, participants), other reported outcomes, information on healthcare utilisation and/or clinical status, key conclusions and limitations. |
| DHI assessment | Used the domains and definitions from the Theoretical Domains Framework by Cane et al. (24) |
