# Supplementary material for: Systematic review of the development and effectiveness of digital health information interventions, compared with usual care, in supporting patient preparation for paediatric hospital care, and the impact on their health outcomes
Source: Front Health Serv. 2023 Apr 6;3:1103624. doi: 10.3389/frhs.2023.1103624 (PMC10117991; doi:10.3389/frhs.2023.1103624)
Supplement: Supplementary file 1 [file Datasheet1.zip › Supplementary files/Appendix E.DOCX]

# Appendix E

# Table 5. study characteristics and DHIs details.

| **Author (year)** | **Country** | **Study design** | **Timing of study** | **Setting for DHI** | **Procedures** | **Inclusion criteria**  ***Exclusion criteria*** | **Type of DHI and details** | **DHI usage and timing** | **Length of DHI** | **Comparator(s)** |
| --- | --- | --- | --- | --- | --- | --- | --- | --- | --- | --- |
| Bray et al. (58) | UK | Before and after evaluation study | Control:  Sept 2018 to January 2019  DHI: January to-Jun 2019 | Home | Non-invasive (x-ray or ultrasound)  Invasive (surgery cannulation and blood tests) | Children aged 8-14 years having a planned clinical procedure.  *Children with a moderate or severe cognitive impairment, or a referral to psychological services for procedural anxiety.* | **Educational interactive multi-media application called Xploro Digital Therapeutic (DTx) platform**. Provided as a preloaded App on iPad and uses augmented reality, gameplay and artificial intelligence to deliver health information about the hospital environment (wards and operating room), key staff, hospital equipment, sensory aspects of the procedure and information on coping strategies. | Multiple, a minimum 3 days before the procedure | Not stated | **Usual care.** Normal hospital information was provided. |
| Stunden et al. (59) | Canada | Triple-arm unblinded, RCT^1^ | July 2019 to Feb 2020 | Hospital | MRI | Children aged 4-13 years.  *Children with mental disability, current concussion, significant visual or auditory impairment, inability to speak and understand English, history of seizures or epilepsy, facial or head wounds, or inability to move their head in all directions.* | **Virtual reality simulation mobile application.** VR guided tour of the hospital environment, the imaging room, and steps of a head scan with interactive user-activated hotspots.  Used MERGE VR headset and Samsung S9, with mobile phone inserted into the front panel of a headset. Parents watched on a tablet device in parallel to the child using AirServer Connect (App Dynamic ehf) to mirror the VR-MRI sequence. | Once on the same day | Max 45 mins | **Usual care.** Hospital’s standard preparatory manual for non-sedated MRI is provided. It contains a series of photos showing the MRI experience step-by-step.  **Child Life Program (CLP)**. The child receives conventional care, where a Certified Child Life Specialist (CCLS) supports preparing the child and parent, individualised, and adapted to the child. |
| Ryu et al. (60) | South Korea | Prospective RCT | June to Oct 2017 | Hospital | Surgery:  -otolaryngology  -ophthalmic  -orthopaedic  -dental  -other | Children aged 4-10 years with ASA^2^ physical status of I or II, undergoing general anaesthesia and elective surgery.  *Children requiring major surgery or postoperative intensive care, with a history of prematurity or congenital disease, a hearing impairment, cognitive impairments, anaesthesia experience, taking psychoactive medications or a history of epilepsy or seizure.* | **Virtual reality 360-degree video tour.** Video provides information about the procedure, hospital environment including the wards and equipment, and the staff. It takes the viewer through the process from admission to the operating theatre, and Pororo explains the process throughout in detail. It starts with Pororo changing into a hospital gown, having an IV catheter placed in his forearm and then going into the operating room. VR tour is provided as a mobile App using a smartphone (Samsung Galaxy S6) and VR headset Samsung VR Gear). | Once, 1 hour before going into operating room in a separate room | 4 mins | **Usual care.** Standard information regarding the process of anaesthesia and surgery was provided verbally. |
| Ryu et al. (61) | South Korea | RCT | Jan to April 2017 | Hospital | Elective surgery and general anaesthesia | See Ryu et al. (2017) for details. | **Virtual reality (VR) 360-degree video tour.**  See Ryu et al. (2017) for details. | Once, 1 hour before going into operating room in a separate room | 4 mins | **Usual care.** Standard information regarding the process of anaesthesia and surgery was provided verbally. |
| Wright et al. (62) | Canada | Triple-arm RCT | June 2016 to June 2018 | Home | Day procedures under general anaesthesia via mask induction  -ENT^3^ -Urology -General -Orthopaedic -Dental  -Ophthalmic | Children aged 9-11 years undergoing common paediatric day surgery procedures who the research team felt could benefit from preparation provided by the I-PPP.  *Children with a history of central nervous system disease, liver disease, renal disease, cancer, neurological or cognitive impairment or disease, or presence of participation barriers (e.g., unable to read or understand English, lack of access to the Internet).* | **Interactive web-based program called Interactive, internet-delivered preoperative preparation program (I-PPP)**. I-PPP is an interactive, internet-delivered preoperative preparation program providing details across 5 modules for both children and parents: (1) education about the day surgery process via an interactive, virtual tour of the hospital (including admission area, day surgery room, holding area, operating room, and activities that take place in those locations); (2) education about anaesthesia and anaesthesia protocol (i.e., what is anaesthesia, types, purpose, process);  (3) identification of emotions and thoughts associated with the day surgery experience (e.g., anxiety, worry);  (4) behavioural training (i.e., shaping and exposure to anaesthetic mask with mask provided by the researcher; and coping instructions for parents to support their child); and  (5) practice of skills (i.e., instructions regarding the continued practice of shaping and exposure to an anaesthetic mask).  Modules are reviewed sequentially with parent direction and can be logged into at different times. | Multiple, 1 week before surgery | Each module is 5-10 mins | **Usual care.** Details are not stated beyond treatment as usual.  **I-PPP+p.** The I-PPP+p group included parental presence during anaesthesia. This group received additional instruction on the induction process on the day of surgery. |
| Park et al. (63) | South Korea | Prospective RCT | Jan and Feb 2018 | Hospital | Surgery:  -otolaryngology  -ophthalmic  -orthopaedic  -dental | See Ryu et al. (2017) for details. | **Virtual reality (VR) 360-degree video tour with mirroring display.**  See Ryu et al. (2017) for details of VR video. Includes mirroring display for parents to watch simultaneously.  VR tour is provided as a mobile App using a smartphone (Samsung Galaxy S6) and VR headset Samsung VR Gear). Mirroring display provided using Smart Mirroring 2.0 SE. | Once, 1 hour before going into operating room in a separate room | 4 mins | **Virtual reality (VR) 360-degree video.** Children only use VR tours, no mirroring display for parents. |
| Wantanakorn et al. (64) | Thailand | Single-blinded, RCT | May 2015 and May 2016 | Two medical centres | Bone marrow aspiration | Children aged 5-12 years undergoing bone marrow aspiration in paediatric wards of either medical centre.  *Children with unstable vital signs, impaired consciousness, or an intellectual disability.* | **Audio-visual (video) via Mobile application with games**. Short, animated video with cartoon child patients and doctors using easy words about bone marrow aspiration procedures. It provides information on the whole process of bone marrow aspiration from positioning, local anaesthesia, and sedation to post-procedure recovery procedure, and instruments used. It includes a game section to help children cope with anxiety including breathing exercises and matching the medical instruments.  Participants in the intervention group also received routine information. | Once, the day before the procedure | Not stated | **Usual Care.** Paediatric residents and oncological fellows provide verbal patient information about bone marrow aspiration and cover preparation for post-procedure management on the day before the procedure. |
| Huntington et al. (65) | UK | Triple-arm double-blinded RCT | July 2012 to Dec 2013 | Home and Hospital | Tooth extraction under general anaesthesia | Children aged 5-7 years with ASA physical status of I scheduled for GA tooth extraction.  *Children excluded with known medical or behavioural conditions, if the family had insufficient internet capability for "YouTube", and children who had already had a GA when older than 2 years.* | **Web-based click-through presentation and standard care (fasting and wound care instructions)**. Intervention-video group provided access to www.scottga.org which had 22 screens for families to click through and a cartoon story, and 2 videos which model appropriate behaviour and teach coping skills | Multiple, 1 week before procedure and again on arrival on the ward | Not stated | **Usual care.** Standard information pack on fasting and wound care, a colouring book about healthy food choices.  **Handwashing game (placebo-video).** Same standard care materials plus access to a hand-washing video of similar length and target age to the DHI group, 1 week before the procedure and on the ward on the day of the procedure. |
| Fernandes et al. (67) | Portugal | Triple-arm RCT | Jan 2012 to Feb 2013 | Hospital | Minor ambulatory surgery:  -circumcisions  -excisions -herniorrhaphies | Children 8-12 years scheduled for minor ambulatory surgery, accompanied by a parent.  *Non-Portuguese speaking children or children with non-Portuguese speaking parents, children with other underlying complicating conditions, and developmental delays.* | **Educational interactive multimedia application "An Adventure at the Hospital".** Multimedia application with seven levels to illustrate hospital procedures and stages for (1) admission, (2) staff and hospital rules, (3) medical instruments, (4) medical procedures, (5) surgery room, (6) recovery room, and (7) aftercare and going home. Each level has a brief video with a child of the same age explaining a specific topic of the intervention, involving sensorial (e.g., the appearance of the operating room) and procedural information (e.g., rules regarding food and clothing). Includes interactive games related to previous modelling videos for each level with a report of the child's feelings by choosing the facial expression of the game character at the end of each interactive activity (sadness, happiness, anger and fear).  Run on a tablet personal computer (PC) device. | Once, on the day of surgery | 15 mins | **Playing popular video games**. Used in the experimental (comparison) group and children choose which game they want to play (super Mario, angry birds, and FIFA/PES).  **No intervention**. Used as the control group. |
| Hatipoglu et al. (66) | Turkey | Triple-arm RCT | March 2015 to Feb 2016 | Hospital | Selective ambulatory surgery: -orchiopexy,  -hypospadias surgery,  -inguinal hernia,  -tonsillectomy,  -adenoidectomy, -strabismus surgery | Children aged 5-12 years ASA physical Status I-II, scheduled for outpatient surgery.  *Children with chronic illness, undergoing emergency surgery, cognitive disorders, and parents who refused to participate* | **Audio-visual (video) presentation.** The video consists of two parts recorded in the anaesthesia clinic and operating room with hospital doctors, nurses, an 11-year-old female and her mother. It provides information about preoperative preparation, anaesthesia induction and postoperative recovery period, covering what will happen, equipment used, some staff involved and what operating room and recovery rooms look like. Part 1: is 300s verbal explanation by the anaesthesiologist of preoperative information, anaesthesia management and the postoperative period. Part 2 is 44s and provides information on preoperative preparation, anaesthesia induction and recovery period. | Once, 1 week before surgery | 344 s | **Voice recording.** The child listens to the same information provided in the video to the intervention group.  **Usual care.** Standard information is provided verbally on anaesthesia practice of the hospital on anaesthesia and analgesia management, preoperative fasting and regular use of the drug to be administered after surgery. |
| Eijlers et al. (68) | Netherlands | Single-blinded RCT | March 2017 to Oct 2018 | Hospital | Surgery: -Adenoidectomy and/or tonsillectomy  -Tympanostomy tubes -Maxillofacial and dental procedures -Other ENT procedures | Children aged 4-12 years undergoing elective maxillofacial, dental or ENT Day care surgery.  *Children with mental retardation, inability of parents to read or write Dutch, epilepsy, visual impairment, ASA physical status at least III and need for pre-operative anxiolytic medication.* | **Virtual reality exposure (VRE).** Computer-generated virtual reality hospital storyline showing child-friendly information about the hospital environment, staff, equipment and what will happen from admission to recovery. Motion-activated explanation of different instruments when a child points towards them.  Two versions were developed, one for 4- to 7-year-olds and one for 8- to 12-year-olds to attune to developmental level.   Used HTC Vive (HTC Corporation, Xindian, New Taipei, Taiwan) head-mounted display and displayed on a personal computer monitor for the parent to see simultaneously. | Once, on the day of surgery | 15 mins | **Informative online video**. During the preoperative screening consultation, the anaesthesiologist recommends that the child watches an informative online movie of the Erasmus MC-Sophia Children's Hospital general anaesthesia before surgery. |
| Liguori et al. (69) | Italy | Unblinded RCT | Dec 2013 to Sep 2014 | Hospital | Surgical intervention (e.g., abdominal hernia, phimosis, or orthopaedic corrections) | Native Italian-speaking children aged 6-11 years without any cognitive impairment who are having a surgical intervention the next day, with native Italian-speaking parents.  *Non-native Italian children over 12 years under 6 years, with any cognitive impairments, not admitted afternoon before surgery, with non-native Italian speaking parents.* | **Audio-visual (video) tour.** Tour of operating room by two clowns, explaining what the operating room is like and what the equipment is in a joking and funny but technically correct way.  The video was provided via an App on a tablet and questions were answered by a nurse. | Once, the afternoon preceding the planned procedure | 6 mins | **Usual care.** Standard information regarding surgical procedures is provided verbally on the day before the planned procedure, with any questions answered by a nurse. |
| Ryu et al. (70) | South Korea | Prospective RCT | Feb to April 2018 | Hospital | Elective day surgery: -Otolaryngology, -Ophthalmic, -Dental, -other | See Ryu et al. (2017) for details. | **Virtual reality (VR) game**: experience the preoperative process and general anaesthesia induction in a 360-degree, three-dimensional virtual environment in the first person. The game includes a virtual world, progression, exploration, challenges and rewards. It starts with the child changing into a surgical gown and then being transported to the operating theatre and allows the child to interact with the operating room environment, including equipment (ECG^4^, saturation monitor, non-invasive blood pressure etc.) The child can select devices and a detailed description is provided and can practice breathing in a facial oxygen mask chosen based on fragrance. After each instruction in the game, the child is faced with challenges to defeat the germ monster and advances to the next preoperative step being rewarded with health points. The VR uses famous animated characters to explain the process in detail.  A head-mounted VR display, Oculus rift (Oculus VR, Menlo Park, CA, USA), and a hand and finger motion controller, Leap Motion Controller (Leap Motion, San Francisco, CA, USA) were used to play the VR game. | Once, 1 hour before entering operating room | 5 mins | **Usual care.** The conventional mode of education about the pre-operative process is provided verbally. |
| Fortier et al. (71) | US | RCT | Aug 2011 to Aug 2012 | Home | Outpatient surgery: -ENT -general -urology -ophthalmology -plastics -orthopaedic | Children 2-7 years in good health, born at =32 weeks gestational age, and without any developmental delays or cognitive impairment.  *Children with ASA=3, with developmental delay or cognitive impairment that would preclude participating in WebTIPS and may impact surgical recovery, or non-English speaking.* | **Web-based program called Web-based Tailored Intervention for Preparation of parents and children undergoing Surgery (WebTIPS).** The web-based program is individually customisable to personality and baseline characteristic, and surgical characteristics. It is accessible from a computer or laptop, comprising education, skills training and interactive games to prepare children for what to expect before, during and after surgery and to teach coping strategies to manage perioperative anxiety and pain. The parent modules provide information, skills training, anxiety management, and modelling techniques to prepare parents for the day of surgery.  Received standard of care information as well. | Multiple, 7 days before and 7 days after surgery | Various | **Usual care of the hospital.** |
| Campbell et al. (72) | UK | Triple-arm RCT | Aug 2002 to July 2003 | Hospital | Tooth extraction under general anaesthesia | Children 3-10 years with no previous experience of medical or dental general anaesthesia, with English as the first language and their parent/carer.  *Not stated.* | **Web-based click-through presentation.** Composed of 8 screens relating to a child's view of the DGA^5^ visit narrated by a child with a Glasgow dialect. | Once, directly before entering the DGA theatre | Not stated. | **Cartoon strip.** Depicting 12 scenes from the DGA visit laminated onto cardboard.  **Usual care.** Involving verbal preparation and pre-anaesthetic assessment by a nurse, with parents present and able to escort their child into the theatre. |
| Wakimizu et al. (73) | Japan | RCT | Oct 2005 to May 2006 | Home and Hospital | Herniorrhaphy | Children aged 3-6 years undergoing elective herniorrhaphy for inguinal hernia and hydrocele testis.  *Children or their caregivers with chronic pain, problems with any of the five senses, cognitive impairments or other diseases that require special treatments, problems with communication or challenges with reading and writing in Japanese.* | **Audio-visual (video) tour.** Patient-educational video 'Shujutsu ni ikou' uses a modelling approach of the experience of a 5-year-old boy hospitalised for an inguinal hernia. Composed of 12 scenes setting out the preoperative process from packing and preparing at home for the hospital to admission, walking to the operating room and what to expect once in the operating room and providing information on staff and equipment.  Video available for review as many times as the child and parents/caregivers wish in the week before surgery.  Information booklet provided to the caregiver covering basic regulations and guidelines for at-home preparation including how to watch the video and example answers to anticipated questions. | Multiple, a week before surgery | Approx. 9 minutes | **Video.** The Control group watched the same video but only once a week before surgery. |
| Dehghan et al. (74) | Iran | Solomon four-group RCT | Not stated | Hospital | Abdominal surgery | Children 6-12 years having abdominal surgery, with no history of abdominal surgery or cognitive impairments.  *Children struggling to use eyeglasses or headphones in VRET^67^.* | **Virtual reality exposure therapy (VRET).** Simulated steps of going into the operating room through applying eyeglasses in front of a computer monitor, with sounds played through headphones. | Once, timing is not stated | 5 minutes | **Parental presence.** Parents in the control group requested to touch and caress their children before surgery. |
| 1. RCT - randomised controlled trial 2. ASA - American Society of Anaesthesiologist 3. ENT - ear, nose and throat 4. ECG - electrocardiogram 5. DGA - dental general anaesthetic 6. VRET – virtual reality exposure | | | | | | | | | | |

# Table 6. Participant characteristics.

| **Author (year)** | **Population description** | **Number of participants, retention rate (%)**  **The number included for analysis** | **Control(s)**  **Child age, sample size, percentage (%), and retention rate (%)**  **Child sex: male (n,%), female (n,%)** | **Intervention(s)**  **Child age, sample size (n), percentage (%), and retention rate (%)**  **Child sex: male (n,%), female (n,%)** | **Child Ethnicity** | **Parental/ caregiver age** |
| --- | --- | --- | --- | --- | --- | --- |
| Bray et al. (58) | Children aged 8-14 years and their parents/ caregivers | 80 randomised, 100% retention rate  80 included for analysis | Two control groups average age of 10.5 years:   1. 8-10 years, n=16, 40% (rr=100%) 2. 11-14 years: n=24, 60% (rr=100%)   Sex: male n=20 (50%), female n=20 (50%) | Two DHI groups average age of 12 years:   1. 8-10 years: n=20, 50% (rr100%) 2. 11-14 years: n=20, 50% (rr100%)   Sex: male n=16 (40%), female n=24 (60%) | Not provided | Not provided |
| Stunden et al. (59) | Children aged 4-13 years and their parents/ caregivers | 89 randomised, 94% retention rate  84 included for analysis. Of the 89 consenting, 5% were excluded due to equipment malfunction. | Control groups mean ± SD age:  SPM: 8.9 ± 2.8, n=26 allocated, n=24 (28%) included for analysis, (rr=92%) CLP: 9.2 ± 2.7, n=31 allocated, n=30 (36%) included for analysis, (rr=97%)  Sex:  SMP: male n=13 (54%), female n=11 (46%)  CLP: male n=20 (67%), female n=10 (33%) | DHI group mean ± SD age:  VR-MRI: 9.3 ± 2.7, n=32 allocated, n=30 (36%) included for analysis, (rr=94%)  Sex: male n=18 (60%), female n=12 (40%) | Not provided | Not provided |
| Ryu et al. (60) | Children aged 4-10 years | 86 randomised, 93% retention rate  80 included for analysis. Of 86 consenting, 3 had operations cancelled and 3 were excluded due to failure to check PAED score. | Control group median (IQR) age:  6 (5-8), n=43 allocated, n=39 (49%) included for analysis (rr 91%)  Sex: male n=21 (54%), female n=18 (46%) | DHI group median (IQR) age:  7 (5-7), 43 allocated, n=41 (51%) included for analysis (rr 95%)  Sex: male n=29 (71%), female n=12 (29%) | Not provided | Not provided |
| Ryu et al. (61) | Children aged 4-10 years | 70 randomised, 98% retention rate  69 included for analysis. Of the 70 consenting, 1 was excluded due to the child refusing to complete VR due to dizziness. | Control group median (IQR) age:  6 (5-9), n=35 allocated, n=35 (50%) included for analysis (rr 100%)  Sex: male n=24 (69%), female n=11 (31%) | DHI group median (IQR) age:  6 (5-7), n=35 allocated, n=34 (49%) included for analysis (rr 97%)  Sex: male n=17 (50%), female n=17 (50%) | Not provided | Not provided |
| Wright et al. (62) | Children aged 9 to 11 years and their parents/ caregivers | 108 randomised, 98% retention rate  104 included for analysis. Of the 108 consenting, 4 were excluded due to failure to collect data or IV induction) | Control group mean ± SD age:  5.9 ± 2.5, n=38 (35%), n=34 (32%) included for analysis (rr=89%)  Sex: male n= 21 (62%), female n=13 (38%) | Two DHI groups mean ± SD age:  I-PPP: 5.9 ±2.2, n=36 (34%), n=36 (34%) included for analysis (rr 100%) I-PPP +parent: 6.3 ± 2.6, n= 34 (31%), n=34 (32%) included for analysis (rr=100%)  Sex: I-PPP: male n=18 (50%), female n=18 (50%) Sex: I-PPP+parent: male n= 22 (65%), female n=12 35%) | Control: 58.8% Caucasian  I-PPP: 66.7% Caucasian  I-PP+parent: 70.6% Caucasian | Control: 35.5±5.2  I-PPP: 36.3±5.3 (one parent did not give age)  I-PPP+parent: 37.0±6.8 |
| Park et al. (63) | Children aged 4-10 years and their parents/ caregivers | 80 randomised, 100% retention rate  80 included for analysis | Control group median (IQR) age:  81 (64-105), n=40, 50%, (rr=100%)  Sex: male n=20 (50%), female n=20 (50%) | DHI group median (IQR) age:  85 (69-101), n=40, 50%, (rr=100%)  Sex: male n=27 (68%), female n=13 (32%) | Not provided | Not provided |
| Wantanakorn et al. (64) | Children aged 5-12 years | 60 randomised, 100% retention rate  60 included in the analysis | Control group mean ± SD age:  9.57 ± 3.06, n=30, 50%, (rr=100%)  Sex: male n=19 (63%), female n=11 (37%) | DHI group mean ± SD age:  9.22 ± -3.43, n=30, 50%, (rr=100%)  Sex: male n=19 (63%), female n=11 (37%) | Not provided | Not provided |
| Huntington et al. (65) | Children aged 5-7 years and their families | 176 randomised, 94% retention rate  166 were included in the analysis. Of the 176 consenting, 10 were excluded due to GA date changes (n=6) or non-attendance (n=4). Of these, 4 were in the usual care group, 2 in the hand-washing group and 4 in the DHI group. | Control groups mean ± SD age:  Usual care: 6 ± 0.83, n=59 allocated, n=55 (33%) included in analysis (rr=93%) Handwashing: 6 ± 0.80, n=57 allocated, n=55 (33%) included in analysis (rr=96%)  Sex:  Usual care: male n=29 (49%), female n=30 (51%) Handwashing: male n=28 (51%), female n=27 (49%) | DHI group mean ± SD age:  6 ± 0.80, n=60, n=56 (34%) included in analysis (rr=93%)  Sex: male n=29 (48%), female: n=31 (52%) | Not provided | DHI: 19-24: 3 (5%) 25-29: 8 (13.3%) 30-34: 15 (25%) 35-39: 12 (20%) 40+: 19 (31.7%)  Usual care:  19-24: 1 (1.7%) 25-29: 10 (16.9%) 30-34: 9 (15.3%) 35-39: 14 (23.7%) 40+: 14 (23.7%)  Handwashing: 19-24:1 (1.8%) 25-29: 6 (10.9%) 30-34: 10 (18.2%) 35-39: 12 (21.8%)  40+: 12 (21.8%) |
| Fernandes et al. (67) | Children aged 8-12 years and their parents/ caregivers | 90 randomised, unable to confirm retention rate | Age: not stated per group but overall, from 8-12 years 10.20 (1.54)  Entertainment (comparison): n=30 (33.33%)  Control group: n=30 (33.33%)  Sex: not stated per group but overall, 69 (77%) males and 21 (23%) females | Age: not stated per group but overall, from 8-12 years 10.20 (1.54)  DHI: n=30 (33.33%)  Sex: not stated per group but overall, 69 (77%) males and 21 (23%) females | Not provided | Mean age 38.43±5.56 |
| Hatipoglu et al. (66) | Children aged 5-12 years | 99 randomised, 100% retention rate  99 included in the analysis | Control groups mean ± SD age  Control: 7.6 ± 2.3, n=33, 33.34%, (rr=100%) Audio: 7.4 ± 1.9, n=33, 33.34%, (rr=100%)  Sex:  Control: male n=18 (54%), female n=15 (45%) Audio: male n=16 (49%), female n=17 (51%) | DHI group mean ± SD age  7.6 ± 2.0, n=33, 33.34%, (rr=100%)  Sex: male n=17 (51%), female n=16 (49%) | Not provided | Control: male n=13 (39%), female n=20 (61%), age 36.7±5.3  Audio: male n=12 (36%), female n=21 (64%), age 34.6±5.1  DHI: male n=14 (42%), female n=19 (58%), age 36.9±5.4 |
| Eijlers et al. (68) | Children aged 4-12 years and their parents/ caregivers | 200 randomised, 96% retention rate  191 included in the analysis. Of the 200 consenting, 9 excluded due accidental unblinding (n=5), non-compliance with anaesthetic protocol (n=2), no data collected (n=1) and cancelled surgery (n=1). Of these 3 were in the control and 6 in the DHI group.  21 in DHI discontinued intervention as took off the headset. | Control group median (IQR) age:  7.5 (IQR5.6 to 10.7), n=100 allocated, n=97 (51%) included in analysis (rr = 97%)  Sex: male n=56 (57.7%), female n=41 (42.3%) | DHI group median (IQR) age:  8.3 (IQR 5.7 to 10.2), n=100 allocated, n=94 (49%) included in ITT analysis (rr = 94%)   Sex: male n=45 (47.9%), female n=49 (52.1%)  Data excluding those who discontinued VRE: age: 9.0 [IQR 6.4 to 10.7], n=100 allocated, n=73 (77%) included in per-protocol analysis (rr. 73%) | Not provided | Not provided |
| Liguori et al. (69) | Children aged 6-11 years | 40 randomised, 98% retention rate  39 included in the analysis. Of the 40 consenting, 1 was excluded from control due to the operation being cancelled. | Control group mean ± SD age:  8.6 ± 2.2, n=20 allocated, n=19 (48%) included in analysis (rr=95%)  Sex: male n=11 (55%), female n=9 (45%) | DHI group mean ± SD age:  8.8 ± 2.5, n=20, 50%, (rr=100%)  Sex: male n=9 (45%), female n=11 (55%) | Not provided | Control: 41.3 (5.0), n=20, 50%, (rr=95%)  DHI: 41.8 (SD 6.2), n=20, 50%, (rr=100%) |
| Ryu et al. (70) | Children aged 4-10 years and their parents/ caregivers | 70 randomised  69 included in the analysis. Of the 70 consenting, 1 was excluded from DHI due to the operation being cancelled. | Control group median (IQR) age:  6 (IQR 5-8), n=35, 50% (rr=100%)  Sex: male n=22 (63%), female n=13 (37%) | DHI group median (IQR) age:  5 (IQR 5-7), n=35 allocated, n=34 (47%) included for analysis (rr=97%)  Sex: male n=18 (53%), female n=16 (47%) | Not provided | Not provided |
| Fortier et al. (71) | Children aged 2-7 years and their parents/ caregivers | 82 randomised, 100% retention rate  82 included in the analysis. | Control group mean ± SD age:  4.4 ± 1.7, n=44, 53.7%, (rr=100%)  Sex: male n= 22 (27%), female n=22 (27%) | DHI group mean ± SD age:  4.3 ± 1.8, n=38, 46.3%, (rr=100%)  Sex: male n=24 (29%), female n=14 (17%) | Mixed (Caucasian, Hispanic or Latino, Asian, African American, or Multiracial other) | Control: 33.1± 6.8  DHI: 34.7 ± 8.0 |
| Campbell et al. (72) | Children aged 3-10 years | 198 randomised, unable to confirm retention rate | Control groups median (range) age:  Control: 5 years (3-10 years), n=66, 38%, rr not able to calculate  Cartoon: 5 years (3-9 years), n=63, 36%, rr not able to calculate  Sex:  Control: male n=41 (62%), female n=25 (38%) Cartoon: male n=33 (52%), female n=30 (48%) | DHI group median (IQR) age:  5 years (3-8 years), n=63 (36%) rr not able to calculate    Sex: male n=35 (56%), female: n=28 (44%) | Not provided | Not provided |
| Wakimizu et al. (73) | Children aged 3-6 years and their parents/ caregivers | 158 randomised, 91% retention rate  144 were included in the analysis. Of 158 consenting, 14 were excluded due to cancellation of operation (n=8) and 6 due to dropouts. Of these, 6 were in the DHI group and 8 in the control group. | Control group mean ± SD age:  Age in months: 61.1 ± 16.9, n=81 allocated, n=73 (51%) included in analysis, rr=90.1%  3-year-old: n=20 (24.7%) 4-year-old: n=19 (23.5%) 5-year-old: n=21 (25.9%) 6-year-old: n=21 (25.9%)  Sex: male n=50 (59.2%), female n=31 (40.8%) | DHI group mean ± SD age:  age in months: 59.8 ± 15.8, n=77 allocated, n=71 (92%) included in analysis, rr=92.2%  3-year-old: n=21 (27.3%) 4-year-old: n=18 (23.4%)  5-year-old: n=19 (24.7%)  6-year-old: n=19 (24.7%)   Sex: male n=49 (63.6%), female n=28 (36.4%) | Not provided | Control: 20s: n=9 (11.1%) 30s: n=56 (69.1%) 40s: n=13 (16.0%) Others: n=1 (1.2%) No answer: n=2 (2.5%)  DHI: 20s: n=10 (13.0%) 30s: n=53 (68.8%) 40s: n=11 (14.3%) Others: n=1 (1.3%) No answer: n=2 (2.6%) |
| Dehghan et al. (74) | Children aged 6-12 years | 40 randomised, unable to confirm retention rate | Age 7/35 ± 2/05 years, n=20, two groups n=10 in the group without pre-test and n=10 in the group with pre-test  Sex: not stated per group but overall males n=31 (77%), females n=9 (23%) | Age 7/35 ± 2/05 years, n=20 two groups n=10 in the group without pre-test and n=10 in the group with pre-test  Sex: not stated per group but overall males n=31 (77%), females n=9 (23%) | Not provided | Not provided |
