# Supplementary material for: Systematic review of the development and effectiveness of digital health information interventions, compared with usual care, in supporting patient preparation for paediatric hospital care, and the impact on their health outcomes
Source: Front Health Serv. 2023 Apr 6;3:1103624. doi: 10.3389/frhs.2023.1103624 (PMC10117991; doi:10.3389/frhs.2023.1103624)
Supplement: Supplementary file 1 [file Datasheet1.zip › Supplementary files/Appendix F.DOCX]

# Appendix F

# Table 7. Theoretical domains scored in DHIs.

| **Included papers**  **[additional**  **resources]**  **Domains** | **Bray et al. (58)** | **Stunden et al. (59)** | **Ryu et al. (60) Ryu et al. (61)**  **Park et al. (63)** | **Wright et al.**  **(62)** | **Wantanakorn et al. (64)** | **Huntington**  **et al. (65)** | **Fernandes**  **et al. (67)** | **Hatipoglu**  **et al. (66)** | **Eijlers**  **et al. (68)** | **Liguori et al. (69)**  **[Online video (99)]** | **Ryu et al.**  **(70)** | **Fortier et al.**  **(71)**  **[Kain et al. (100)]** | **Campbell**  **et al. (72)** | **Wakimizu**  **et al. (73)** | **Dehghan**  **et al. (74)** | **Number of DHIs scoring 1** | **Number of DHIs scoring 0.5** | **Total DHIs scoring 1 or 0.5** |
| --- | --- | --- | --- | --- | --- | --- | --- | --- | --- | --- | --- | --- | --- | --- | --- | --- | --- | --- |
| 1. **Knowledge** | 1 | 1 | 1 | 1 | 1 | 1 | 1 | 1 | 1 | 0.5 | 1 | 1 | 1 | 1 | 0.5 | **13** | **2** | **15** |
| 1. **Skills** | 1 | 1 | 0 | 1 | 1 | 0.5 | 1 | 0.5 | 0.5 | 0 | 1 | 1 | 0.5 | 0.5 | 0 | **7** | **5** | **12** |
| 1. **Emotion** | 1 | 1 | 0 | 1 | 0.5 | 0.5 | 1 | 0 | 1 | 0.5 | 0 | 1 | 0 | 0 | 0 | **6** | **3** | **9** |
| 1. **Behavioural Regulation** | 1 | 1 | 0 | 1 | 1 | 0.5 | 0 | 0 | 0 | 0 | 1 | 1 | 0 | 0 | 0 | **6** | **1** | **7** |
| 1. **Memory, Attention and Decision Processes** | 0.5 | 1 | 0 | 1 | 0 | 0 | 1 | 0.5 | 0.5 | 0.5 | 1 | 1 | 0 | 0.5 | 0 | **5** | **5** | **10** |
| 1. **Environmental Context and Resources** | 1 | 1 | 1 | 1 | 0.5 | 0.5 | 1 | 1 | 1 | 0.5 | 1 | 1 | 0.5 | 1 | 0.5 | **10** | **5** | **15** |
| 1. **Beliefs about Capabilities** | 1 | 1 | 0 | 1 | 1 | 0 | 0 | 0 | 0 | 0 | 1 | 1 | 0 | 0 | 0 | **6** | **0** | **6** |
| 1. **Beliefs about Consequences** | 1 | 1 | 1 | 1 | 1 | 0 | 1 | 1 | 1 | 0.5 | 1 | 1 | 1 | 0.5 | 0.5 | **11** | **3** | **14** |
| 1. **Reinforcement** | 1 | 1 | 0 | 1 | 1 | 0 | 1 | 0 | 0 | 0 | 1 | 1 | 0 | 0.5 | 0 | **7** | **1** | **8** |
| 1. **Intentions** | 0 | 1 | 0 | 1 | 0.5 | 0 | 0 | 0 | 0 | 0 | 0.5 | 0.5 | 0 | 0 | 0 | **2** | **3** | **5** |
| 1. **Goals** | 0 | 1 | 0 | 0 | 0 | 0 | 0 | 0 | 0 | 0 | 1 | 1 | 0.5 | 0 | 0 | **3** | **1** | **4** |
| 1. **Social influences** | 1 | 0 | 0.5 | 1 | 0 | 0 | 0.5 | 0 | 1 | 0 | 0.5 | 1 | 0 | 0 | 0 | **4** | **3** | **7** |
| 1. **Optimism** | 0 | 0.5 | 0 | 0 | 0 | 0 | 0 | 0 | 0 | 0 | 1 | 0.5 | 0 | 0 | 0 | **1** | **2** | **3** |
| 1. **Co-production** | 1 | 0.5 | 0.5 | 1 | 0.5 | 1 | 0.5 | 0 | 0.5 | 0 | 0.5 | 1 | 0 | 1 | 0 | **5** | **6** | **11** |
| 1. **Use of behaviour framework or approach** | 1 | 0 | 0 | 1 | 0 | 0 | 1 | 0 | 0 | 0 | 0 | 1 | 0 | 0 | 0 | **4** | **0** | **4** |
| **Domains met fully** | 11 | 11 | 3 | 13 | 6 | 2 | 8 | 3 | 5 | 0 | 10 | 13 | 2 | 3 | 0 |  |  |  |
| **Domains met partially** | 1 | 2 | 2 | 0 | 4 | 4 | 2 | 2 | 3 | 4 | 3 | 2 | 3 | 4 | 2 |  |  |  |
| **Total domains scored fully or partially** | 12 | 13 | 5 | 13 | 10 | 6 | 10 | 5 | 8 | 4 | 13 | 15 | 5 | 7 | 2 |  |  |  |

Table 8. Further details on the m-TDF assessment.

| **Included**  **papers**  **Domain** | **Bray**  **(58)** | **Stunden**  **(59)** | **Ryu (60), Ryu (61)**  **Park (63)** | **Wright**  **(62)** | **Wantanakorn**  **(64)** | **Huntington**  **(65)** | **Fernandes**  **(67)** |
| --- | --- | --- | --- | --- | --- | --- | --- |
| **Knowledge** | The platform provides information about the procedure, hospital environment including wards and operating theatres, the key healthcare staff involved and the hospital equipment (p. 3). It uses a customisable avatar as a guide and chatbot. | VR movie and App provides information about the MRI procedure, the hospital equipment and the staff involved (p. 3). It starts by introducing a radiologist and a peer in the reception area and then leads the user through an interactive guided tour of the hospital reception area, imaging room and the steps of a head scan. | VR movie provides information about the procedure, hospital environment including the wards and equipment, and the staff (p. 99 and 1629 respectively). It takes the viewer through the process from admission to the operating theatre, and Pororo explains the process throughout in detail. It starts with Pororo changing into a hospital gown, having an IV catheter placed in his forearm and then going into the operating room. | I-PPP includes two modules on education and information about the day surgery process and anaesthesia and anaesthesia protocol. Delivered via an interactive, virtual tour of the hospital including the admission area, day surgery room, holding area, operating room, and activities that take place in those locations. For anaesthesia, it covers what it is, the types, purpose and process (p.628) | Short, animated video in Mobile App provides patient information about the procedure including equipment used, and what to expect through the whole process from positioning, local anaesthesia, and sedation to post recovery process (p.644). | Online web-based information over several screens/slides setting out a story of a 6-year-old child called Scott going through the process of the procedure, with child and parents 'mouse clicking' through (From p.159 plus additional paper Reynolds et al. (2012). | The App provides information on (1) hospital admission; (2) health care staff and hospital rules; (3) medical instruments; (4) medical procedures; (5) surgery room; (6) recovery room; and (7) aftercare and going home (p.1192). |
| **Skills** | The platform uses a Q&A chatbot and games to create an understanding of the procedure and enable interactive engagement (p. 3) | The App includes elements to help the user learn how to interact with the virtual environment and cues to activate the next steps (p.3) | The movie is not interactive only informative (p. 99 and 1629 respectively). | I-PPP includes a module on skills development through instructions regarding the continued practice of shaping and exposure to an anaesthetic mask (p.628). | The game element is interactive, with breathing skills developed through the relevant game but the movie element is purely informational. | Partially. Included 2 videos which model appropriate coping behaviour and teach coping skills (p.159) | The application uses interactive game activities after each level to guarantee that the information provided was understood by the child (p.1192) |
| **Emotion** | Information provided on sensory aspects of the procedure, and what child may experience or feel to help reduce fear and anxiety (p. 3) | Through the inclusion of familiar sounds using narrative to support the child coping with the loud and noisy MRI sound, to provide comfort thus reducing stress. | Unclear if the movie addresses emotion. Reference made to Pororo emphasising that children will undergo the same process without difficulty, but no reference to considering anything that addresses dealing with fear, anxiety etc (p. 99 and 1629 respectively). | I-PPP includes a module on the identification of emotions and thoughts associated with day surgery experiences like anxiety, and worry (p.628). | The game section is included in App to support child cope with anxiety (p.644) | Partially. Included 2 videos which model appropriate coping behaviour and teach coping skills (p.159) | The application uses a facial expression of the game character for the child to choose whether they are sad, happy, angry or fearful (p.1192).   The application starts with a brief video explaining how to report emotions. |
| **Behavioural Regulation** | Information provided on coping strategies to manage behaviour (p. 3) | App uses staying still to progress through the levels in the VR-MRI app, so drives behavioural regulation to achieve stillness (p.7). | No information was provided. | I-PPP includes a module on behavioural training through shaping and exposure to an anaesthetic mask with a mask provided (p.628) | The game section included in App provides breathing exercises and coping skills (p.644) | Partially. Included 2 videos which model appropriate coping behaviour and teach coping skills (p.159) | No information was provided to judge if the application supports any behavioural regulation. |
| **Memory, Attention and Decision Processes** | Partially. Designed with children to ensure is engaging, acceptable and effective. Also considers ease of navigation. (p. 3) | User-led tour of hospital, staff and equipment through interactive hotspots designed to activate transition between rooms or sequences, use of stalling sequence to deal with any distractions (p.3 and 4). | Unclear. While the movie was short being only 4-minutes long it is not clear from the papers whether a child's attention and memory were factored into its development. | I-PPP is interactive and initially provided sequentially but once completed, the child and parents can return to any module (p.628). | Unclear. It is not clear from the papers whether a child's attention and memory were factored into its development. No indication that the child could re-watch the video. | Unclear as insufficient information provided in paper or other papers identified and reviewed. | The application uses interactive game activities after each level to guarantee that the information provided was understood by the child (p.1192) |
| **Environmental Context and Resources** | Provides information and images of the hospital environment (wards and operating theatres), equipment and key staff. (p. 3) | Provides information and images of the hospital environment, equipment, and staff. Also includes stimulations of sounds during an MRI. (p.3 and 4) | Provides information and images of the hospital environment, equipment, and staff. Includes scenes of having an intravenous catheter inserted, pressure cuff and pulse oximeters placed, and a facial mask applied for anaesthesia (p. 99 and 1629 respectively). | Module 1 is an interactive, virtual tour of the hospital including the admission area, day surgery room, holding area, operating room, and activities that take place in those locations (p.628). | Partially. Provides information on instruments used and images suggest some context of the hospital setting (p.644), but not as detailed as with other applications/ tools, so partially met. | Partially. Appears to provide information on the procedure but is unclear on the level of information provided (p.159) | The application provides information on health care staff and hospital rules; medical instruments; surgery room; recovery room (p.1192). |
| **Beliefs about Capabilities** | Assumed through reference to what child may experience and building of coping strategies - empowerment, self-confidence (p. 3) | Assumed through levels becoming more challenging to achieve as feedback mechanism reduced and children required to continue independently (p.7) | Unclear. The video narration is noted to emphasise that all children undergo the same process without difficulty but no references to coping, emotions, or feelings (p.99) | I-PPP includes a module on coping instructions for parents to support their child (p.628), and behavioural training (p.628) | Breathing exercises and games help in coping with anxiety, help build self-confidence and provide empowerment (p.644) | Unclear as insufficient information provided in paper or other papers identified and reviewed. | Unclear as insufficient information provided in the paper, and no indication of building confidence or esteem through coping. |
| **Beliefs about Consequences** | Understanding of the procedure and what the outcome will be through information on how it may feel or what may be experienced (p. 3) | Assumed through not progressing the levels due to not staying still (p.7) | Taking the child through a narrative guided tour explaining the processes and showing what equipment is used (mask, ECG, blood pressure cuff etc.) is deemed to support building an understanding of the consequences of the process. | Assumed through detailed virtual tour of hospital and experience, and behavioural component, specifically shaping and exposure to anaesthesia mask (p.628) and practice of skills module. | Assumed through the provision of information on the whole bone marrow aspiration process through step-by-step guided video (p.644) showing what happens by using cartoon children and healthcare professionals. | Unclear as insufficient information provided in paper or other papers identified and reviewed. | The use of the interactive game to confirm if the child has understood the information provided and explanation of procedural information including rules. |
| **Reinforcement** | Use of interactive games | Stalling of the sequence until the child's attention is refocussed appropriately (p.4). Use of stillness as a measure to progress through levels (p.7). | Simple 4-minute video taking the child through a guided tour from admission to the operating theatre, but no use of interactive tools nor implied if the child could rewatch the video. | I-PPP is interactive and initially provided sequentially but once completed, the child and parents can return to any module (p.628). Participants were encouraged to use the I-PPP more than once, particularly the behavioural component. Modules on the practice of skills, coping instructions and emotions reinforce desired behaviour. | Breathing exercises and games to help in coping with anxiety, help reinforce behaviour to reduce anxiety (p.644) | Unclear as insufficient information provided in paper or other papers identified and reviewed. | The application uses interactive game activities after each level to guarantee that the information provided was understood by the child (p.1192) |
| **Intentions** | Not demonstrated in the paper | Used interactive real-time feedback on indicators of movement to support progression through the levels (p.7) | No due to the intervention being a video | Assumed through behavioural components, specifically shaping and exposure to anaesthesia mask (p.628). | Partially met through breathing exercises to promote specific behaviour. | Unclear as insufficient information provided in paper or other papers identified and reviewed. | Does not address this. |
| **Goals** | Not demonstrated in the paper | Used interactive real-time feedback on indicators of movement to support progression through the levels (p.7) | No due to the intervention being a video | Not demonstrated | Not demonstrated | Unclear as insufficient information provided in paper or other papers identified and reviewed. | Unclear as insufficient information provided in the paper but suspect not. |
| **Social influences** | In a supplementary paper (081) covering information on parents being with a child or being supported by something familiar. | Unclear from paper or methodology used in development. | Partially. VR game incorporates a famous animated character to socialise the content in a child-friendly manner (p.3) | Inclusion of a parent and child path in the program that would support discussion with the child (Wright et al. 2020, p.306 and 307) | Not demonstrated | Unclear as insufficient information provided in paper or other papers identified and reviewed. | Partially met through reference to information on parental separation (p.1192). |
| **Optimism** | Not demonstrated. | Partially. Includes level attainment but unclear from the paper if level attainment is set out in advance for children to create optimism in achieving level 3. | No due to the intervention being a video | Not demonstrated. | Not demonstrated. | Not demonstrated. | Not demonstrated. |
| **Co-production with: - healthcare professionals - children - parents** | YES. Developed with all three. | PARTIAL: Developed with the research team, various healthcare professionals and system administrators. No indication of development with children or parents. | PARTIAL: Script for movie developed by anaesthesiologist from Seoul National University Bundang Hospital, with doctors and nurses acting in the movie. | YES. Developed and tested with all three through separate studies (see Wright et al. 2017) | PARTIAL: Mobile App was tested with healthcare professionals and a small sample of children (p.645). No evidence that parents were involved. | YES: The paper notes that expert consultation and focus groups were used to develop the tool (p.158 and 163) with this supported by information in Reynolds et al. (2012) and the study protocol. | PARTIAL: The paper notes that a pilot study involved 490 children and healthcare professionals to improve the application (p.1192). No reference to the involvement of parents in the development of the tool. |
| **Used a behaviour framework or** | YES: Used person-based approach as described by Yardley et al. (2015) [paper 082] | Unclear. Used an agile development methodology but unclear if a behavioural approach was used as part of this. | NO. No reference was provided to ascertain whether any behavioural frameworks were used. | YES. Paper Wright et.al (2017) notes that cognitive behavioural intervention for anxiety disorders in children and behavioural preparation was used in developing the components of I-PPP (p.49). | Unclear. Reference made to development team studying characteristics and requirements of young children, but not to use of behavioural frameworks or tools (p.645). | Unclear as insufficient information provided in paper or other papers identified and reviewed. | Yes. Paper references Social Learning Theory's theoretical framework (p. 1191) and that certain behaviours can be learned and reproduced, with modelling being effective to reinforce self-efficacy. |

| **Included**  **papers**  **Domain** | **Hatipoglu**  **et al. (66)** | **Eijlers**  **et al. (68)** | **Liguori et al. (69)**  **[Online video (99)]** | **Ryu et al.**  **(70)** | **Fortier et al.**  **(71)**  **[Kain et al. (100)]** | **Campbell**  **et al. (72)** | **Wakimizu**  **et al. (73)** | **Dehghan**  **et al. (74)** |
| --- | --- | --- | --- | --- | --- | --- | --- | --- |
| **Knowledge** | Video recording that informs about the surgery and anaesthesia methods, what the duties of the anaesthesiologist are, what will happen and what equipment is used and some of the hospital staff involved in the procedure, and what the operating and recovery rooms look like (p.794) | The virtual reality storyline shows the child information about the hospital environment, staff, equipment and what will happen from admission to recovery (p.730). Different instruments can be explained by the child by pointing towards them creating an interactive explanation. | Partially. Video uses two clowns Dr Cloud and Dr Wisp who, funnily and engagingly, show the operating room and explain some of the equipment used (p.2). It does not provide any information on staff or the wider hospital environment. | Virtual reality storyline with game elements explaining the preoperative process through a 360-degree, three-dimensional virtual environment in first-person perspective. It uses famous characters to explain the preoperative process from putting on the hospital gown to being transported into the operating room and includes information on equipment, staff and how to use the anaesthetic mask etc (p.3). | The intervention provides web-based information across 4 modules: (1) at home before surgery, (2) holding area and anaesthesia induction, (3) recovery room and (4) at home after surgery (p.908). Provides information on the hospital environment, staff and equipment through videos and games. | The computer package provides information on the process before dental extraction. It includes details of the staff involved by clicking on each of their images and explains that the child is sent to sleep when 'magic wind from a space mask' is applied to the face. It provides some information on recovery when Scott wakes up and feels 'fizzy' and provides aftercare information (p.833). | Video provides information across 12 scenes from pre-hospital preparation (1,2 and 3) to arriving at the hospital and meeting the staff, preparing for surgery by changing and leaving the caregiver to walk to the operating room (4, 5, 6 and 7), to confirming child's identity and getting ready for surgery having equipment applied (8, 9, 10, 11 and 12). It provides information on the whole preoperative process, hospital environment and staff (p.395) | Partially. The intervention uses VR-simulated steps, viewed through eyeglasses in front of a computer monitor, of going to the operation room, with headphones placed on the child's ears to stimulate the sounds of the virtual environment (p.3). The Paper states information provided on the ward and operating room but otherwise lacks details to meet this domain fully. |
| **Skills** | Partially. The movie is not interactive but only informative (p.794), however paper states (p.789) that visuals modelling body language together with auditory information of two key elements for effective learning methods. | Partially. The child can point at different instruments with a motion-tracked controller and the staff in the environment then explain what they are for (p.730). | The movie is not interactive only informative (p.2) | VR incorporates games to challenge the child to defeat the germ monster and awards 'health points' each time child advances to the next preoperative step (p.3). | Use of games and videos to build coping skills, modelling behaviour and tailors the experience to the child's fear levels by providing information about the surgical process and the child character Anna displays more or less fearful responses (p.909 to 911). | Partially. At the end of the computer package, a list of activities is provided to support the prevention of tooth decay to enable improved oral health skills (p.833). However, insufficient information is provided to determine if other skills are addressed such as coping mechanisms. | Partially. Video available for multiple reviews in advance of surgery during the week before, allowing the child to develop an understanding of the preoperative process. However, the provided booklet for caregivers contained information on coping techniques which the child didn't have direct access to (p395). | Insufficient information was provided to judge if VR stimulated environment develops skills and was only viewed once lasting approx. 5 minutes (p.1). |
| **Emotion** | Unclear if the movie addresses emotion. Reference made to a child asking about pain and being told that pain relief is used, but no indication of any emotional coping (p.794) | VR includes a video with a nurse explaining what kind of feelings the child may experience e.g., nausea (p.730) | Partially. Dr Wisp explains that he is scared and shivering, and Dr Cloud tells him it is ok and that you do not need to feel like that (reference: video clip online) | Unclear if emotions are addressed in this VR game. | Intervention address emotion by tailoring information provided and fear responses of the character Anna to the child accessing the site (p.911) | No information to suggest emotions are addressed in the detail provided in the paper. Appears to largely be informative about the process and staff involved. | The video description in the paper appears to not address emotions with this covered by the booklet for the caregiver (p395).   Therefore scored 0 as not part of digital intervention. | Insufficient information is provided to judge if VR stimulated environment addresses emotions. |
| **Behavioural Regulation** | No information provided | Does not appear that VR includes any behavioural regulation and is purely informative. | The video does not address any behavioural regulation. | VR game incorporates breathing practice (p.3) | The intervention provides techniques for the self-management of anxiety through deep breathing and guided imagery. This is aimed at helping to reduce anxiety and lessen feelings of nervousness (p. 909 and 910). Also uses distraction techniques to help the child manage anxiety (p.910) and module 4 provides instruction on implementing behavioural strategies to minimise pain and distress (p.910). | The computer package does not appear to address behavioural regulation. | The video description in the paper appears to not address behavioural regulation with this covered by the booklet for the caregiver (p395).   Therefore scored 0 as not part of digital intervention. | Insufficient information is provided to judge if VR stimulated environment addresses behavioural regulation. |
| **Memory, Attention and Decision Processes** | Partially. Paper states (p.788 and 789) that behavioural programs to teach coping skills through modelling need to consider the child's age, developmental stage, and previous experience. It is therefore assumed that this was factored into the development of this program but is insufficient to fully meet the domain. | Partially. Two versions of the VR video were produced to address developmental differences in children aged 4 to 12 years, possibly considering memory retention ability and comprehension. | Partially. The video is short approx. 6 minutes, simple and engaging using humour and playfulness (p.2 and online video clip). | VR game is short, uses famous childhood characters from the animated film 'Hello Carbot' and has challenges and rewards to keep the child engaged (p.3) | The intervention uses animated characters Billy Bot and his sidekick Tot Bot to help the child navigate through the modules, using videos, games and humour to engage the child (p.911). A memory game is included to introduce the child to objects in PACU.   Additional printable resources are provided to reinforce learning from web-based modules.  Website is available 5 days before surgery and up to 10 days after surgery and can be accessed 24 hours a day, 7 days a week (p.913) | Unclear. While the computer package seems to be short it is not clear from the papers whether a child's attention and memory were factored into its development, nor how long it takes to navigate the package. | Partially. The video is short at approx. 9 minutes and provided a week in advance for children and caregivers to access as many times as they wished enabling information to be absorbed in a relaxed environment (p.395).  However, no interactive elements to this tool. | Insufficient information is provided to judge if VR stimulated environment addresses this domain. |
| **Environmental Context and Resources** | Video recording shows the equipment, operating room and recovery room. It also explains what to expect in terms of how the anaesthesia mask is used by demonstrating on a teddy bear. It includes the anaesthesiologist and nurse (p.794). | VR video provides detailed information and images of the hospital environment, staff and equipment (p.730). | Partially. The video includes information on the operating room and some of the equipment and explains the use of the pulse oximeter and anaesthesia mask but no information on staff or the wider hospital environment, including the recovery process (p.2 and online video clip) | VR storyline and games provide detailed information and images of the operating room environment, equipment and staff (p.3) | The intervention provides information on the hospital environment, staff and equipment through videos and games. It also uses a specific game to introduce the child to objects in the operating room (p.911) | Partially. The computer package includes information on staff and explains the use of the pulse oximeter and anaesthesia mask but no information wider environment or other equipment (p.833) | Video provides information on the hospital environment, staff and equipment (p.395) | Partially. The intervention uses VR-stimulated steps, viewed through eyeglasses in front of a computer monitor, of going to the operation room, with headphones placed on the child's ears to stimulate the sounds of the virtual environment (p.3). The Paper states information provided on the ward and operating room but otherwise lacks details to meet this domain fully. |
| **Beliefs about Capabilities** | Unclear as insufficient information provided in the paper, and no indication of building confidence or esteem through coping. | Unclear as insufficient information provided in the paper, and no indication of building confidence or esteem through coping. | Does not address this domain. The video is purely information provision and does not incorporate any activities or interaction to enable building self-confidence, self-esteem etc. | Assumed through the use of the challenging game to defeat germ monster (p.3) | Games used are designed to 'model, reinforce and practice strategies for keeping calm and reducing nervousness' (p.911). | Does not address this domain. The computer package is purely information provision and does not incorporate any activities or interaction to enable building self-confidence, self-esteem etc. | Does not address this domain. The video is purely information provision and does not incorporate any activities or interaction to enable building self-confidence, self-esteem etc. | Does not address this. |
| **Beliefs about Consequences** | The movie explains that pain is managed by anaesthesia and that child will fall asleep quickly and then awake after surgery with the parent in recovery. It shows how the anaesthesia will be administered through a vessel in the hand (p.794) | Assumed through step-by-step storyline from admission to anaesthesia, including enabling interactive motion control to seek additional information on equipment (p.730) | Partially. The video includes information on the operating room and some of the equipment, and explains the use of the pulse oximeter and anaesthesia mask this gives a sense of consequences, but insufficiently addresses this domain to score a 1 (p.2) | Assumed through child advancing through step-by-step preoperative process and interaction of operating room environment (p.3) | Assumed using tailoring information to the child's level of fear, through the provision of information about the surgical process and use of video and games on objects in the operating room and PACU and placing of anaesthesia masks on animals (p.911) | The computer package includes information about staff and some of the equipment and explains the use of the pulse oximeter and anaesthesia mask giving a sense of consequences. It also provides a list of activities to support the prevention of tooth decay to enable improved oral health skills (p.833). | Partially. The video includes information on the preoperative process, including the equipment and staff, but insufficiently addresses this domain to score a 1 (p.395) | Partially. VR stimulated environment includes information on the steps of going into the operating room but doesn't fully address this domain to score a 1 (p.3) |
| **Reinforcement** | A simple video explaining specific information about the preoperative expectations, but no use of interactive tools nor implied if the child could rewatch the video. | Although VR video uses interactive motion-controlled gesturing, this is simply to enable the further provision of information and no indication that any behaviour reinforcement is applied. | A simple video explaining specific information about the preoperative expectations, but no use of interactive tools nor implied if the child could rewatch the video. | VR uses interactive game activities after instructions to challenge the germ monster, it also includes the choice of a facial oxygen mask to practice breathing (p.3) | The use of deep breathing exercises, memory games for objects in the PACU and placing the anaesthesia mask on animals provides reinforcing behaviours (p.911). Additional printable activities, including colouring sheets, are also provided. | Simple cartoon computer package explaining specific information about staff and some of the processes, but no use of interactive tools nor implied if the child could rewatch the sequence. | Partially. Video is available for multiple reviews in advance of surgery during the week before, allowing the child to develop an understanding of the preoperative process (p395). | Does not address this. |
| **Intentions** | Does not address this. | Does not address this. | Does not address this. | Partially. Using breathing exercises and interactive gameplay with rewards (p.3). | Partially. Using breathing and coping exercises for both child and parents (p909 and 911). | Does not address this. | Does not address this. | Does not address this. |
| **Goals** | Does not address this. | Does not address this. | Does not address this. | VR game element uses rewards when the child advances to the next step (p.3) | Provision of certificate for completion of the program, advancement through the modules by playing games (p.911) | Partially. At the end of the computer package, a list of activities is provided to support the prevention of tooth decay to enable improved oral health skills (p.833). | Does not address this. | Does not address this. |
| **Social influences** | Does not address this. | VR video confirms that parents can stay with the child all the time until they are anaesthetised, it includes images of the parent and child wearing hospital gowns (p.730) | Does not address this. | Partially. VR game incorporates a famous animated character to socialise the content in a child-friendly manner (p.3) | Parental modules of the site aimed at teaching parents coping and modelling behaviours, with this part accessed before the child site which parents then go through with their child (p.908, 909) | Does not address this. | Does not address this. | Does not address this. |
| **Optimism** | Not demonstrated. | Does not address this. | Does not address this. | Assumed through rewarding 'health points' when child advances levels (p.3) | Partially met. Use of tailoring to create feelings of being safe and managing anxiety levels. Use of memory game to introduce objects in PACU and provision of completion certificate. | Does not address this. | Does not address this. | Does not address this. |
| **Co-production with: - healthcare professionals - children - parents** | No information was provided on the development of the video. | PARTIAL: A multidisciplinary team, consisting of child life specialists, child psychologists, a child psychiatrist, anaesthesiologists, a three-dimensional (3D) acting director, and a 3D project manager designed the script of the VRE (p.2). Paper indicated tested by children and adjusted to consider feedback, but no indication of parental involvement. | Beyond confirming that the video was produced in collaboration with the association of Soccorso Clown, it does not confirm if healthcare staff, children or parents were involved. While this may be assumed, it is not stated. | PARTIAL: study authors developed video in collaboration with VR game producing company, with study authors being healthcare professionals (confirmed via searching) | YES: Programme developed using task force of anaesthesiologists, psychologists, surgeons, nurses, paediatricians, child life specialists, parents, and children (p.906 Kain et al. (2015) paper) | No information was provided on the development of the computer package and referenced paper was not accessible. | YES: Video developed and edited by various medical staff (outpatient unit, surgical ward and operation department) and reviewed by families and experts (p.395) | No information was provided on the development of VR stimulated environment. |
| **Used a behaviour framework** | No information was provided on the development of video and therefore use of any behavioural frameworks. | Unclear. While various experts used to inform development, including psychologists it is unclear if any behaviour frameworks were used. | No information was provided on the development of video and therefore use of any behavioural frameworks. | No information was provided on the development of VR games and therefore use of any behavioural frameworks. | Yes. Paper Kain et al. (2015) states that the 'conceptual framework and content of WebTIPS was examined by a behavioural medicine, interventions, outcomes expert panel' (p.906). | No information was provided on the development of the computer package and referenced paper was not accessible. | No information was provided to determine is any theoretical frameworks used. | No information was provided. |

Table 9. Details of the type of assessments and the studies in which they applied.

| **Assessment of emotions and feelings – children and/or parents** | **Description** | **Applied in the following studies** |
| --- | --- | --- |
| Visual Analogue Scale (VAS) - children | Self-rated scale to assess emotions, including anxiety in context and intensity of the emotion. | Bray et al. (58), Wantanakorn et al. (64), Eijlers et al. (68), and Campbell et al. (72) |
| The State-Trait Anxiety Inventory (STAI) – parents | Anxiety inventory of 20 items related to state or trait anxiety | Stunden et al. (59), Wright et al.(62), Fernandes et al. (67), Eijlers et al. (68), Fortier et al. (71), and Wakimizu et al. (73) |
| Venham Picture Test – children | A pictorial measure of child anxiety during medical procedures | Stunden et al. (59) |
| Facial Image Scale (FIS) – children | 5-point pictorial faces scale from extremely anxious to not anxious | Huntington et al. (65) |
| Child Surgery Worries Questionnaire (CSWQ) – children | Self-reported 23-item scale for assessing worries about hospitalization, medical procedures, illness, and its negative consequences | Fernandes et al. (67) |
| The Self-Assessment Manikin – children | Non-verbal pictorial assessment of arousal (calm or aroused) and valence (happy or unhappy). | Fernandes et al. (67) |
| Wong-Baker Faces Scale (FACES) | Pictorial scale on the intensity of anxiety/pain from a happy to crying face | Wakimizu et al. (73) |
| Numerical rating scale (NRS) | One-item rating scale to define satisfaction | Park et al. (63) |
| Modified Child Dental Anxiety Scale (MCDAS) – parents | Pictorial scale ranging from 8-40 to assess the level of dental anxiety | Campbell et al. (72) |
| Temperament Survey for Children: Parental Ratings (EAS-P) | 20-item scale on four temperaments: emotionality, activity, sociability, and shyness | Fernandes et al. (67) – no results reported |
| Emotionality, Activity, Sociability, and Impulsivity Temperament Scale (EASI) – children | 20-item scale on four temperaments: emotionality, activity, sociability, and shyness | Wright et al. (62) – no results reported |
| **Assessment of behaviour** | **Further description** | **Applied in the following studies** |
| modified Yale Preoperative Anxiety Scale (mYPAS) | Observational rating scale covering five items: activity, vocalisations, emotional expressivity, state of apparent arousal and use of parent | Ryu et al. (60), Ryu et al. (61), Wright et al. (62), Park et al. (63), Wantanakorn et al. (64), Huntington et al. (65), Hatipoglu et al. (66), Eijlers et al. (68), Liguori et al. (69), Ryu (70), Fortier et al. (71) |
| Standardised Yale Preoperative Anxiety Scale (YPAS) | The observational rating scale covers four items: activity, vocalisations, emotional expressivity, and state of apparent arousal. | Dehghan et al. (74) |
| Visual Analogue Scale (VAS) | 10-cm long visual scale to score behaviour during induction with 10 equating to the worst behaviour | Huntington et al. (65) |
| Paediatric Anaesthesia Emergence Delirium (PAED) scale | Observational rating scale over five behaviours determining the degree of ED | Ryu et al. (60), Eijlers et al. (68), and Fortier et al. (71) |
| Incidence of ED | Number of children experiencing ED | Ryu et al. (60) |
| Procedural behaviour rating scale (PBRS) | Observational scale to identify behaviours (e.g., verbal pain, expressions of fear or anxiety, seeking emotional support and physical restraint) | Ryu et al. (61), and Ryu et al. (70) |
| Induction compliance checklist (ICC) | Observational scale to identify induction compliance, with ratings related to fear, anxiety, and negative behaviour | Ryu et al. (61), Wright et al. (62) Park et al. (63), and Ryu et al. (70) |
| Face, Legs, Arms, Cry, Consolability (FLACC) scale | Observational rating scale to identify emotional, verbal, or behavioural expressivity | Eijlers et al. (68) |
| Post-hospitalisation behavioural questionnaire (PHBQ) | Observational 27-item rating scale covering six categories: general anxiety, separation anxiety, sleep anxiety, eating disorder, aggressive behaviour against the authority, and apathy and withdrawal | Ryu et al. (60), Hatipoglu et al. (66) |
| Child Behaviour Checklist (CBCL) | Observational scale to identify attention problems, aggressive behaviour, and anxiety | Eijlers et al. (68) |
| Parents’ Postoperative Pain Measure (PPPM) | Observational 15-item scale to identify postoperative pain in children and its impact | Eijlers et al. (68) |
| **Assessment of physiological changes** | **Further description** | **Applied in the following studies** |
| Cardiovascular assessments | Changes in heart rate and blood pressure as a reaction to stimuli | Fernandes et al. (67) |
| **Assessment of clinical status** | **Further description** | **Applied in the following studies** |
| The Faces Pain Scale-Revised (FPS-R) | A self-report 0-to-10 measure of pictorial pain intensity for children | Eijlers et al. (68) |
| NRS | One-item rating scale to define pain | Fortier et al. (71) |
| Patient flow | Length of treatment or surgery, length of time in recovery or on the ward, measured in minutes | Fortier et al. (71), and Huntington et al. (65) |
| Medication usage | Amount of medication usage such as for sedation, pain management | Wantanakorn et al. (64) Eijlers et al. (68), and Fortier et al. (71) |
| Head movement | A measure of head movement in MRI threshold at 3-4mm | Stunden et al. (59) |
| Preparation and assessment time | Measured in minutes where preparation time is that spent preparing for the procedure using group assigned intervention and assessment time is the length of MRI stimulation | Stunden et al. (59) |
| **Assessment of usability, satisfaction, and knowledge** | **Further description** | **Applied in the following studies** |
| Visual Analogue Scale (VAS) | Rating scale to record satisfaction, knowledge, fun etc. (applied differentially across studies, with different rating scales) | Bray et al. (58), Stunden et al. (59), and Huntington et al. (65). |
| Numerical rating scale (NRS) | One-item rating scale to define satisfaction | Ryu et al. (61), Park et al. (63) and Ryu et al. (70) |
| Client Satisfaction Questionnaire (CSQ) | 8-item measure to assess client satisfaction | Wright et al. (62) |
| Smilyometer Likert Scale | From fun toolkit and is a non-verbal pictorial scale to determine how fun the DHI was to use | Stunden et al. (59) |
| Usefulness, Satisfaction and Ease of use (USE) | 7-point Likert rating scale for assessing the usability of a product or service | Stunden et al. (59) |
| Treatment Evaluation Inventory (TEI) | 19-item measure of treatment acceptability | Huntington et al. (65) |
| Generic rating scales, including the Likert scale or tick box forms | Created by researchers to determine satisfaction, degree of knowledge or information given, and tick box form used to determine parts of application child used and liked | Bray et al. (58), and Wakimizu et al. (73) |

Table 10. Study outcomes and measurements, and direction of effect calculation.

| **Author (year)** | **Primary outcome(s)**  **Secondary outcome(s)** | **Measures** | **Timings** | **Sample** | **Outcomes**  [mean (SD) unless otherwise stated] | | **Effect size and study reported findings** |  |
| --- | --- | --- | --- | --- | --- | --- | --- | --- |
| Bray et al. (58) | Trait anxiety (child & parents) | 10-point VAS (self-reported) | 3-5 days before the procedure | C=40  I= 40 | **Parents (3-5 days before):**  Control: 2.15 (0.770)  Intervention: 2.10 (0.709)  p=0.76  **Children (3-5 days before):**  Control: 2.35 (0.700)  Intervention: 2.38 (0.49)  p=0.85 | | **Parents:**  Cohen's d = (2.1 - 2.15) ⁄ 0.740129 = 0.067556  Glass's delta = (2.1 - 2.15) ⁄ 0.77 = 0.064935  Hedges' g = (2.1 - 2.15) ⁄ 0.740129 = 0.067556  Finding: no significant difference between groups.  **Children:**  Cohen's d = (2.38 - 2.35) ⁄ 0.604194 = 0.049653  Glass's delta = (2.38 - 2.35) ⁄ 0.7 = 0.042857  Hedges' g = (2.38 - 2.35) ⁄ 0.604194 = 0.049653  Finding: no significant difference between groups. |  |
|  | Procedural (state) anxiety (child & parents) | 10-point VAS (self-reported) | 3-5 days before the procedure and on arrival at the hospital | C=40  I= 40 | **Parents (3-5 days before):**  Control: 6.00 (2.97)  Intervention: 5.40 (2.193)  p=0.31  **Children (3-5 days before):**  Control: 6.98 (2.70)  Intervention: 6.68 (1.51)  p=0.54  **Parents (on arrival at hospital):**  Control: 6.18 (2.836)  Intervention: 5.10 (1.1919)  p=0.05  **Children (on arrival at hospital):**  Control: 7.15 (2.63)  Intervention: 5.82 (1.57)  p=0.008 | | **Parents:**  Cohen's d = (5.4 - 6) ⁄ 2.61057 = 0.229835  Glass's delta = (5.4 - 6) ⁄ 2.97 = 0.20202  Hedges' g = (5.4 - 6) ⁄ 2.61057 = 0.229835  Finding: no significant difference between groups, small effect.  **Children:**  Cohen's d = (6.68 - 6.98) ⁄ 2.187476 = 0.137144  Glass's delta = (6.68 - 6.98) ⁄ 2.7 = 0.111111  Hedges' g = (6.68 - 6.98) ⁄ 2.187476 = 0.137144  Finding: no significant difference between groups, small effect.  **Parents:**  Cohen's d = (5.1 - 6.18) ⁄ 2.175261 = 0.496492  Glass's delta = (5.1 - 6.18) ⁄ 2.836 = 0.380818  Hedges' g = (5.1 - 6.18) ⁄ 2.175261 = 0.496492  Finding: procedural anxiety scores were significantly lower in the intervention group.  **Children:**  Cohen's d = (5.82 - 7.15) ⁄ 2.165849 = 0.614078  Glass's delta = (5.82 - 7.15) ⁄ 2.63 = 0.505703  Hedges' g = (5.82 - 7.15) ⁄ 2.165849 = 0.614078  Finding: procedural anxiety scores were significantly lower in the intervention group. |  |
|  | Procedural knowledge (child & parent) | 10-point VAS (self-reported) | 3-5 days before the procedure and on arrival at the hospital | C=40  I= 40 | **Parents (3-5 days before):**  Control: 5.28 (2.29)  Intervention: 6.13 (1.70)  p=0.06  **Children (3-5 days before):**  Control: 4.32 (2.66)  Intervention: 5.85 (1.83)  p=0.006  **Parents (on arrival at hospital):**  Control: 5.200 (2.267)  Intervention: 6.28 (1.26)  p=0.01  **Children (on arrival at hospital):**  Control: 4.36 (2.66)  Intervention: 6.75 (1.51)  p<0.001 | | **Parents:**  Cohen's d = (6.13 - 5.28) ⁄ 2.016693 = 0.421482  Glass's delta = (6.13 - 5.28) ⁄ 2.29 = 0.371179  Hedges' g = (6.13 - 5.28) ⁄ 2.016693 = 0.421482  Finding: procedural knowledge scores marginally higher in the intervention group.  **Children:**  Cohen's d = (5.85 - 4.32) ⁄ 2.283035 = 0.67016  Glass's delta = (5.85 - 4.32) ⁄ 2.66 = 0.575188  Hedges' g = (5.85 - 4.32) ⁄ 2.283035 = 0.67016  Finding: procedural knowledge scores significantly higher in the intervention group.  **Parents:**  Cohen's d = (6.28 - 5.2) ⁄ 1.83397 = 0.588887  Glass's delta = (6.28 - 5.2) ⁄ 2.267 = 0.476401  Hedges' g = (6.28 - 5.2) ⁄ 1.83397 = 0.588887  Finding: procedural knowledge scores significantly higher in the intervention group.  **Children:**  Cohen's d = (6.75 - 4.36) ⁄ 2.162834 = 1.105032  Glass's delta = (6.75 - 4.36) ⁄ 2.66 = 0.898496  Hedges' g = (6.75 - 4.36) ⁄ 2.162834 = 1.105032  Finding: procedural knowledge scores significantly higher in the intervention group. |  |
|  | Procedural satisfaction (child & parent) | 10-point VAS (self-reported) | After the procedure up to 10 minutes | C=40  I= 40 | **Parents:**  Control: 6.63 (2.457)  Intervention: 6.80 (1.800)  p=0.72  **Children:**  Control: 5.98 (2.787)  Intervention: 6.88 (2.002)  p=0.10 | | **Parents:**  Cohen's d = (6.8 - 6.63) ⁄ 2.1537 = 0.078934  Glass's delta = (6.8 - 6.63) ⁄ 2.457 = 0.06919  Hedges' g = (6.8 - 6.63) ⁄ 2.1537 = 0.078934  Finding: procedural satisfaction was higher in the intervention group, but no overall effect.  **Children:**  Cohen's d = (6.88 - 5.98) ⁄ 2.426456 = 0.370911  Glass's delta = (6.88 - 5.98) ⁄ 2.787 = 0.322928  Hedges' g = (6.88 - 5.98) ⁄ 2.426456 = 0.370911  Finding: procedural satisfaction was higher in the intervention group. |  |
|  | Procedural involvement (child) | 5-point Likert scale (self-reported) | After the procedure up to 10 minutes | I=20 | p=0.03 | | Unable to calculate Cohen’s d due to the data reported.  Finding: procedural involvement was significantly higher in the intervention group. |  |
|  | Intervention engagement (child) | Yes/no tick-box for parts liked (self-reported) | After the procedure up to 10 minutes | I=20 | Qualitative measure | | Unable to calculate Cohen’s d due to the data reported.  Finding: 20 children who completed this section reported they liked the different components. |  |
| Stunden et al. (59) | Successful MRI stimulation | Head movement threshold 3-4mm | During MRI stimulation | 76 | **The average number of participants scoring above the threshold**  X^2^_2_= 2.7, P=0.07  **Differences between groups**  X^2^_2_= 2.6, P=0.27 | | The p-value of Chi-square:  d = 0.425  95% C.I = -0.0347, 0.8846  v = 0.055  Finding: no statistically significant difference in the average number of times a participant scored above the 4mm movement threshold, but a small effect was observed.  The p-value of Chi-square:  d = 0.2551  95% C.I = -0.1982, 0.7084  v = 0.0535  Finding: no statistically significant difference in simulated experience between the three groups, with a small negative effect with more participants successful in the control groups (C1=47% and C2=50%) compared to intervention (30%). |  |
|  | Child anxiety | Venham Picture Test scored 0-8 (child completed assessment) | Before preparing (T1), after preparing and upon entering the simulation room (T2) and after MRI stimulation (T3) | C1=24  C2=30  I=30 | **Child anxiety before (T1) and after preparing (T2)**  *Control 1 (Standard Preparatory Manual -SPM):*  -T1: median 0, IQR 1; SD 1.521  -T2: median 1, IQR 2; SD 2.311  *Control 2 (Child Life Program - CLP):*  -T1: median 0, IQR 0; SD 1.240  -T2: median 0, IQR 0; SD 1.350  *Intervention (VR-MRI group):*  -T1: median 0, IQR 1; SD 1.311  -T2: median 0, IQR 1; SD 0.819  **Child anxiety upon entering MRI stimulation (T3)**  *Control 1 (SPM):* median 0, IQR 1; SD 1.738  *Control 2 (CLP):* median 0, IQR 0; SD 0.468  *Intervention (VR-MRI group):* median 0, IQR 1; SD 0.434 | | Unable to calculate Cohen’s d due to data reported for all time points due to not providing IQR in full.  Finding: child anxiety was comparable across all groups and no significant differences were observed. Potential that children in the sample are generally less anxious. Children in the intervention group (VR-MRI) and control 2 (CLP) were anxiety free at all time points, while children in control 1 (SPM) were anxiety free before preparation and after the assessment but reported low anxiety after preparation upon entering the MRI simulation room. |  |
|  | Parental anxiety | STAI 6-item adapted version (parent completed assessment) | Before preparing (T1), after preparing and upon entering the simulation room(T2) and after MRI stimulation (T3) | C1=24  C2=30  I=30 | **Parental anxiety was measured before (T1) and after preparing (T2)**  *Control 1 (SPM):*  -T1: median 8, IQR 6; SD 4.945  -T2: median 10, IQR 6; SD 4.394  *Control 2 (CLP):*  -T1: median 7, IQR 6; SD 3.294  -T2: median 8, IQR 4; SD 3.224  *Intervention (VR-MRI group):*  -T1: median 6.5, IQR 4; SD 2.572  -T2: median 6, IQR 2; SD 1.744  **Parental anxiety measured upon entering MRI stimulation (T3)**  *Control 1 (SPM):*  -T3: median 8, IQR 4; SD 2.924  *Control 2 (CLP):*  -T3: median 6, IQR 4; SD 2.545  *Intervention (VR-MRI group):*  -T3: median 6, IQR 2; SD 1.810 | | Unable to calculate Cohen’s d due to data reported for all time points due to not providing IQR in full.  Finding: parental or caregiver anxiety increased in the control 1 (SPM) group from before to after preparation, decreasing to the original level upon entering the MRI stimulation. Parental or caregiver anxiety in the control 2 (CLP) group was compared across the three time points reporting low anxiety overall. Parental or caregiver anxiety in the intervention (VR-MRI) group was also compared across the three time points reporting low anxiety overall, and the lowest among the three groups. However, no clinical significance was noted between the three groups. |  |
|  | Procedural data | Preparation time  Assessment time | Started after the study staff finished describing the programme and was completed when the participant felt ready to take the assessment (maximum of 45 minutes to align to allotted time with a CLP).  Time spent in preparation room until participant discharged from experience (maximum 20 minutes). | C1=24  C2=30  I=30  C1=24  C2=30  I=30 | **Preparation time**  *Control 1 (SPM):* 9.98 minutes  *Control 2 (CLP):* 15.06 minutes  *Intervention (VR-MRI group):* 22.05 minutes  p<0.001  **Assessment time**  *Control 1 (SPM):* 11.19 minutes  *Control 2 (CLP):* 10.17 minutes  *Intervention (VR-MRI group):* 11.79 minutes  p=0.13 | | η^2^=0.568  Finding: preparation time for intervention (VR-MRI) group longer than controls and statistically significant.  Unable to calculate Cohen’s d due to the data reported.  Finding: assessment time comparable across the three groups, with intervention (VR-MRI) slightly higher, but no statistical significance reported. |  |
|  | Parental usability | USE questionnaire 7-point Likert rating (parent completed assessment) | After preparation | C1=24  C2=30  I=30 | *Control 1 (SPM):*  - somewhat agreed it was useful (median 28.5, IQR 6; SD 3.323)  - agreed easy to use (median 24, IQR 5; SD 2.167)  - agreed easy to learn (median 18, IQR 3; SD 2.183)  *Control 2 (CLP):*  - agreed was useful (median 30, IQR 6; SD 4.163)  - agreed easy to use (median 24, IQR 4; SD 2.937)  - agreed easy to learn (median 18, IQR 3; SD 3.059).  *Intervention (VR-MRI group):*  - agreed was useful (median 31, IQR 4; SD 3.562)  - agreed easy to use (median 24, IQR 3; SD 2.448)  - agreed easy to learn (median 18, IQR 2; SD 2.366)  Ease of use p=0.99  Ease of learning p=0.48  Ease of usefulness p=0.48 | | Unable to calculate Cohen’s d due to not providing IQR in full.  Finding: no significant difference was found between groups in terms of parental or caregiver usability. Parents or caregivers in control 1 (SPM) somewhat agreed it was useful and agreed it was easy to use and learn. Parents or caregivers in the control 2 (CLP) and intervention (VR-MRI) group agreed it was useful, easy to use and learn, with results comparable. |  |
|  | Child satisfaction and fun | Child satisfaction used VAS (0-100 scale) (self-reported)  Child fun used Smilyometer Likert Scale (self-reported) | After preparation  Before and after preparation | C1=20  C2=27  I=30 | **Child satisfaction:**  *Control 1 (SPM)*: median 73.5 (IQR 37, SD 27)  *Control 2 (CLP):* median 90 (IQR 23, SD 12)  *Intervention (VR-MRI group):* median 80 (IQR 22, SD 27)  **Child fun**  - Control 1 (Standard Preparatory Manual): “okay”  - Control 2 (Child Life Program): “really good”  - Intervention (VR-MRI group): “really good” | | Unable to calculate Cohen’s d due to not providing IQR in full.  Finding: children in control 1 (SPM) were significantly less satisfied with on average 73.5% of children satisfied. Children in the control 2 (CLP) and intervention (VR-MRI) group were more satisfied with on average 90% and 80% respectively reporting higher satisfaction. |  |
| Ryu et al. (60) | Incidence of ED | n (observed) | 5 minutes after PACU stay | C=14  I=16 | Control: 14 out of 39 participants (36%)  Intervention: 16 out of 41 participants (39%)  p=0.773 | | Finding: no significant difference between groups in terms of incidence of ED. |  |
|  | Severity of ED | PAED Scale (observer-rated) | 5 minutes after PACU stay | C=14  I=16 | *Control:* median 8 (IQR 5-12) converted to 8.3639 (5.7662)  *Intervention*: median 8 (IQR 3.5-12.5) converted to 8 (7.3154)  p=0.719 | | Cohen's d = (8 - 8.3639) ⁄ 6.586507 = 0.055249  Glass's delta = (8 - 8.3639) ⁄ 5.7662 = 0.063109  Hedges' g = (8 - 8.3639) ⁄ 6.641224 = 0.054794  Finding: no significant difference between groups in terms of severity of ED. |  |
|  | Child preoperative anxiety | Korean version of m-YPAS (observer-rated) | Before intervention (admission) and just before induction | C=39  I=41 | **Before intervention (baseline):**  *Control:* median 51.7 (31.7-61.7) converted to 48.15 (23.2896)  *Intervention:* median 46.7 (32.5-55.9) converted to 44.9262 (17.9768)  p=0.346  **At induction:**  *Control:* median 46.7 (33.3-63.3) converted to 47.836 (23.0896)  *Intervention:* median 38.3 (23.3-50.9) converted to 37.4486 (21.2035)  p=0.022 | | **Before intervention (baseline):**  Cohen's d = (44.9262 - 48.15) ⁄ 20.691725 = 0.155801  Glass's delta = (44.9262 - 48.15) ⁄ 23.0896 = 0.139621  Hedges' g = (44.9262 - 48.15) ⁄ 20.626576 = 0.156294  Finding: no significant difference between groups in terms of anxiety before intervention.  **At induction:**  Cohen's d = (37.4486 - 47.836) ⁄ 22.16662 = 0.468606  Glass's delta = (37.4486 - 47.836) ⁄ 23.0896 = 0.449874  Hedges' g = (37.4486 - 47.836) ⁄ 22.142447 = 0.469117  Finding: m-YPAS scored significantly decreased in the intervention group. |  |
|  | Post-operative behavioural disturbances | PHBQ-AS (observer asked questions) | Telephoning parents/caregivers 1 to 14 days after the procedure |  | At 1-day post procedure: p=0.671  *Control:* 0 (0-0)  *Intervention:* 0 (0-0)  At 14 days post-procedure: p=0.329  *Control:* 0 (0-0)  *Intervention:* 0 (0-0) | | Unable to calculate due to data available in paper.  Finding: no significant difference between groups postoperatively. |  |
| Ryu et al. (61) | Child preoperative anxiety | Korean version of m-YPAS (observer-rated) | 30 minutes after receiving intervention | C=35  I=34 | *Control:* median 51.7 (IQR 28.3-63.3) converted to 47.5043 (27.0543)  *Intervention*: median 31.7 (IQR 23.3-37.9) converted to 30.9174 (11.2995)  P<0.01 | | Cohen's d = (30.9174 - 47.5043) ⁄ 20.731785 = 0.800071  Glass's delta = (30.9174 - 47.5043) ⁄ 27.0543 = 0.613097  Hedges' g = (30.9174 - 47.5043) ⁄ 20.840257 = 0.795907  Finding: children in the intervention group had significantly lower anxiety compared to the control group. |  |
|  | Induction compliance | ICC (observer-rated) | During induction | C=35  I= 34 | *Control ICC score*:  -perfect: n=12 (34%)  -moderate: n=17 (49%)  -poor: n=6 (17%)  *Intervention ICC score*:  -perfect: n=28 (82%)  -moderate: n=5 (15%)  -poor: n=1 (3%)  P<0.001 | | Cohen’s d = 0.8629  95% C.I = 0.3489 (1.3768)  v = 0.0688  Finding: more children in the intervention group had perfect compliance compared to the control group. |  |
|  | Procedural behaviour | PBRS (observer-rated) | During induction | C=35  I= 34 | *Control*: median 1 (IQR 0-4)  *Intervention*: median 0 (IQR 0-1)  p=0.01 | | Unable to calculate Cohen’s d due to non-normal data distribution.  Finding: significantly lower PBRS scores in the intervention group. |  |
|  | Parental satisfaction | NRS where 1 = very dissatisfied to 10 = very satisfied (self-reported) | After procedure | C=35  I= 34 | *Control:* median 9.5 (IQR 9-10) converted to 9.5 (0.773)  *Intervention*: median 10 (IQR 9-10)  p=0.198 | | Unable to calculate Cohen’s d due to non-normal data distribution.  Finding: no significant difference between groups in terms of parental satisfaction. |  |
| Wright et al. (62) | Child anxiety | m-YPAS (observer-rated) | Before procedure (T1), transfer to the operating room (T2) and during induction (T3) | C1=34  C2=34  I=36 | **Before procedure (T1):**  *Control 1*: 32.0 (10.7)  *Control 2 (I-PPP+parent):* 32.8 (16.6)  *Intervention (I-PPP):* 33.1 (10.2)  **Transfer to the operating room (T2):**  *Control 1*: 41.1 (20.8)  *Control 2 (I-PPP+parent):* 39.6 (20.8)  *Intervention (I-PPP):* 36.8 (15.3)  **Anaesthetic Induction (T3):**  *Control 1*: 53.4 (25.5)  *Control 2 (I-PPP+parent):* 48.7 (23.9)  *Intervention (I-PPP):* 41.5 (18.3) | | **The effect between Control 1 and Intervention**  Cohen's d = (33.1 - 32) ⁄ 24.396004 = 0.045089  Glass's delt = (33.1 - 32) ⁄ 10.7 = 0.102804  Hedges' g = (33.1 - 32) ⁄ 24.684054 = 0.044563  **The effect between Control 2 and Intervention**  Cohen's d = (33.1 - 32.8) ⁄ 25.99423 = 0. 011541  Glass's delta = (33.1 - 32.8) ⁄ 16.6 = 0.018072  Hedges' g = (33.1 - 32.8) ⁄ 26.219627 = 0.011442  Finding: anxiety between groups comparable before the procedure with no significant effect observed.  **The effect between Control 1 and Intervention**  Cohen's d = (36.8 - 41.1) ⁄ 18.258286 = 0.23551  Glass's delta = (36.8 - 41.1) ⁄ 20.8 = 0.206731  Hedges' g = (36.8 - 41.1) ⁄ 18.17815 = 0.236548  **The effect between Control 2 and Intervention**  Cohen's d = (36.8 - 39.6) ⁄ 18.258286 = 0.153355  Glass's delta = (36.8 - 39.6) ⁄ 20.8 = 0.134615  Hedges' g = (36.8 - 39.6) ⁄ 18.17815 = 0.154031  Finding: anxiety between groups comparable at T2 transfer to operating room having increased from T1 at baseline. A small negative effect was observed in the intervention group compared to control 1, with the usual care group experiencing the highest anxiety  **The effect between Control 1 and Intervention**  Cohen's d = (41.5 - 53.4) ⁄ 22.193918 = 0.536183  Glass's delta = (41.5 - 53.4) ⁄ 25.5 = 0.466667  Hedges' g = (41.5 - 53.4) ⁄ 22.089191 = 0.538725  **The effect between Control 2 and Intervention**  Cohen's d = (41.5 - 48.7) ⁄ 21.284971 = 0.338267  Glass's delta = (41.5 - 48.7) ⁄ 23.9 = 0.301255  Hedges' g = (41.5 - 48.7) ⁄ 21.203177 = 0.339572  Finding: anxiety between groups again comparable at T3 induction having increased from T1 (baseline) and T2 (transfer to the operating room). However, a medium negative effect was observed between intervention and control 1 and a small negative effect between intervention and control 2, showing reduced anxiety in the intervention group across time to the control group 1 and 2. |  |
|  | Induction compliance | ICC (observer-rated) | During induction | C1=34  C2=34  I=36 | *Control 1*: 2.33 (2.84)  *Control 2 (I-PPP+parent):* 1.53 (2.34)  *Intervention (I-PPP):* 1.06 (1.73) | | **The effect between Control 1 and Intervention**  Cohen's d = (1.06 - 2.33) ⁄ 2.351436 = 0.540096  Glass's delta = (1.06 - 2.33) ⁄ 2.84 = 0.447183  Hedges' g = (1.06 - 2.33) ⁄ 2.33552 = 0.543776  **The effect between Control 2 and Intervention**  Cohen's d = (1.06 - 1.53) ⁄ 2.057729 = 0.228407  Glass's delta = (1.06 - 1.53) ⁄ 2.34 = 0.200855  Hedges' g = (1.06 - 1.53) ⁄ 2.048839 = 0.229398  Finding: Induction compliance was associated with a medium negative effect between intervention and control 1 and a small negative effect between intervention and control 2. |  |
|  | Child temperament | EASI (parental self-reported) | 1 week before the procedure | N/A | N/A | | N/A – no analysis was done on this data due to internal inconsistencies ranging from poor to adequate. |  |
|  | Parental anxiety | STAI-T (self-reported)  STAI-S (self-reported) | 1 week before the procedure (baseline)  On the day before the procedure and after the procedure | C1=34  C2=34  I=36  C1=34  C2=34  I=35  C1=34  C2=34  I=33 | **Parental trait anxiety at baseline:**  *Control 1*: 33.6 (9.0)  *Control 2 (I-PPP+parent):* 33.9 (9.2)  *Intervention (I-PPP):* 33.6 (8.1)  **Parental strait anxiety pre-procedure:**  *Control 1*: 33.7 (10.7)  *Control 2 (I-PPP+parent):* 35.4 (8.1)  *Intervention (I-PPP):* 40.0 (10.9)  **Parental strait anxiety post-procedure:**  *Control 1*: 26.9 (7.9)  *Control 2 (I-PPP+parent):* 31.4 (8.6)  *Intervention (I-PPP):* 30.2 (7.5) | | **The effect between Control 1 and Intervention**  Cohen's d = (33.6 - 33.6) ⁄ 8.561834 = 0  Glass's delta = (33.6 - 33.6) ⁄ 9 = 0  Hedges' g = (33.6 - 33.6) ⁄ 8.548607 = 0  **The effect between Control 2 and Intervention**  Cohen's d = (33.6 - 33.9) ⁄ 8.667468 = 0.034612  Glass's delta = (33.6 - 33.9) ⁄ 9.2 = 0.032609  Hedges' g = (33.6 - 33.9) ⁄ 8.651309 = 0.034677  Findings: parental anxiety comparable at baseline, with no effect observed between groups.  **The effect between Control 1 and Intervention**  Cohen's d = (40 - 33.7) ⁄ 10.800463 = 0.583308  Glass's delta = (40 - 33.7) ⁄ 10.7 = 0.588785  Hedges' g = (40 - 33.7) ⁄ 10.801955 = 0.583228  **The effect between Control 2 and Intervention**  Cohen's d = (40 - 35.4) ⁄ 9.602604 = 0.479037  Glass's delta = (40 - 35.4) ⁄ 8.1 = 0.567901  Hedges' g = (40 - 35.4) ⁄ 9.623254 = 0.478009  Findings: parental anxiety was higher in the intervention group pre-procedure with a medium effect observed in comparison to control 1 and a small effect observed in comparison to control 2.  **The effect between Control 1 and Intervention**  Cohen's d = (30.2 - 26.9) ⁄ 7.702597 = 0.428427  Glass's delta = (30.2 - 26.9) ⁄ 7.9 = 0.417722  Hedges' g = (30.2 - 26.9) ⁄ 7.705672 = 0.428256  **The effect between Control 2 and Intervention**  Cohen's d = (30.2 - 31.4) ⁄ 8.068767 = 0.148722  Glass's delta = (30.2 - 31.4) ⁄ 8.6 = 0.139535  Hedges' g = (30.2 - 31.4) ⁄ 8.077204 = 0.148566  Findings: parental anxiety decreased pre- to post-procedure, with a small effect observed in the intervention group compared to control 1 but no effect compared to control 2. However, anxiety is lowest in control 1. |  |
|  | Parental satisfaction | CSQ (parental self-reported) | After procedure | C1=34  C2=34  I=33 | *Control 1*: 29.2 (4.3)  *Control 2 (I-PPP+parent):* 30.9 (1.5)  *Intervention (I-PPP):* 29.9 (2.4) | | **The effect between Control 1 and Intervention**  Cohen's d = (29.9 - 29.2) ⁄ 3.482097 = 0.201028  Glass's delta = (29.9 - 29.2) ⁄ 4.3 = 0.162791  Hedges' g = (29.9 - 29.2) ⁄ 3.49613 = 0.200221  **The effect between Control 2 and Intervention**  Cohen's d = (29.9 - 30.9) ⁄ 2.00125 = 0.499688  Glass's delta = (29.9 - 30.9) ⁄ 1.5 = 0.666667  Hedges' g = (29.9 - 30.9) ⁄ 1.994492 = 0.501381  Findings: parental satisfaction was highest in control group 2, with a negative medium effect observed compared to the intervention group, suggesting parents were more satisfied when able to be present at induction. |  |
| Park et al. (63) | Child preoperative anxiety | Korean version of m-YPAS (observer-rated) | Before admission and before induction | C=40  I=40 | **Child preoperative anxiety at admission**  *Control:* median 36.7 (IQR 23.3-47.5) converted to 35.7773 (18.6081)  *Intervention:* median 32.5 (IQR 23.3-47.5) converted to 34.5583 (18.6081)  p=0.743  **Child preoperative anxiety before induction**  *Control*: median 38.3 (IQR 23.3-44.2) cannot convert due to skewed data  *Intervention:* median 28.3 (IQR 23.3-36.7) converted to 29.5066 (10.3037)  p=0.025 | | **Child preoperative anxiety at admission**  Cohen's d = (34.5583 - 35.7773) ⁄ 18.6081 = 0.065509  Glass's delta = (34.5583 - 35.7773) ⁄ 18.6081 = 0.065509  Hedges' g = (34.5583 - 35.7773) ⁄ 18.6081 = 0.065509  Finding: child preoperative anxiety similar at admission.  **Child preoperative anxiety before induction**  Unable to calculate Cohen’s d due to non-normal data distribution.  Finding: significant difference in m-YPAS scored for the intervention group before induction than in the control group. |  |
|  | Induction compliance | ICC (observer-rated) | During induction | C=40  I=40 | *Control ICC score:*  -perfect: n=26 (65%)  -moderate: n=13 (32%)  -poor: n=1 (3%)  *Intervention ICC score:*  -perfect: n=30 (75%)  -moderate: n=9 (22%)  -poor: n=1 (3%)  p=0.722 | | Cohen’s d = 0.0796  95% C.I = -0.359 (0.5182)  v = 0.0501  Finding: no significant difference between the groups. |  |
|  | Parental preoperative anxiety | NRS using a 101 scale where 0 = no anxiety, 100 = high anxiety (self-reported) | Before admission and before induction | C=40  I=40 | **Parental preoperative anxiety before admission**  *Control:* median 55 (IQR 27.5-80) converted to 54.1128 (40.3688)  *Intervention:* median 50 (IQR 23.75-70) converted to 47.782 (35.563)  p=0.503  **Parental preoperative anxiety before induction**  *Control*: median 55 (IQR 40-80) converted to 58.5488 (30.7572)  *Intervention:* median 30 (IQR 10-62.5) converted to 34.4359 (40.3688)  p=0.009 | | **Parental preoperative anxiety before admission**  Cohen's d = (47.782 - 54.1128) ⁄ 38.041865 = 0.166417  Glass's delta = (47.782 - 54.1128) ⁄ 40.3688 = 0.156824  Hedges' g = (47.782 - 54.1128) ⁄ 38.041865 = 0.166417  Finding: no significant difference between the groups, with no effect.  **Parental preoperative anxiety before induction**  Cohen's d = (34.4359 - 58.488) ⁄ 35.886246 = 0.670232  Glass's delta = (34.4359 - 58.488) ⁄ 30.7572 = 0.781999  Hedges' g = (34.4359 - 58.488) ⁄ 35.886246 = 0.670232  Finding: preoperative anxiety in parents was significantly lower in the intervention group. |  |
|  | Parental satisfaction | NRS using 101 scale where 0 = very dissatisfied and 100 = very satisfied (self-reported) | After procedure | C=40  I=40 | Control: median 85 (IQR 70-100) converted to 85 (23.0679)  Intervention: median 100 (IQR 90-100) cannot convert due to skewed data  p=0.008 | | Unable to calculate Cohen’s d due to non-normal data distribution.  Finding: satisfaction in the intervention group was higher than in the control group. |  |
| Wantanakorn et al. (64) | Child anxiety | Child-A-VAS (self-reported)  Post-intervention Child-A-VAS (self-reported)  m-YPAS (observer-rated) | DHI and control group: an hour before routine information on the day of the procedure  DHI group: after routine information and after completing all parts of DHI or within a maximum of 1 hour  On the day of the procedure after the intervention | C=30  I=30 | **Child self-reported anxiety at baseline**  *Contro*l: 6.56 (1.77)  *Intervention*: 6.70 (2.76)  p=0.82  **Child self-reported anxiety post-DHI intervention**  *Contro*l: not reported  *Intervention*: 5.06 (2.70)  p=0.012  **The child observed anxiety post-intervention**  *Contro*l: 63.08 (13.05)  *Intervention*: 55.37 (12.86)  p=0.001 | | **Child self-reported anxiety at baseline**  Cohen's d = (6.7 - 6.56) ⁄ 2.318459 = 0.060385  Glass's delta = (6.7 - 6.56) ⁄ 1.77 = 0.079096  Hedges' g = (6.7 - 6.56) ⁄ 2.318459 = 0.060385  Finding: anxiety at baseline between groups similar.  **Child self-reported anxiety in intervention group post-DHI intervention compared to baseline**  Cohen's d = (5.06 - 6.7) ⁄ 2.730165 = 0.600696  Glass's delta = (5.06 - 6.7) ⁄ 2.76 = 0.594203  Hedges' g = (5.06 - 6.7) ⁄ 2.730165 = 0.600696  Finding: anxiety in the intervention group significantly decreased after using DHI compared to baseline.  **The child observed anxiety post-intervention**  Cohen's d = (55.37 - 63.08) ⁄ 12.955348 = 0.595121  Glass's delta = (55.37 - 63.08) ⁄ 13.05 = 0.590805  Hedges' g = (55.37 - 63.08) ⁄ 12.955348 = 0.595121  Finding: anxiety in the intervention group was significantly lower compared to the control. |  |
|  | Amount of sedation drugs | Dose of drug ug/kg or mg/kg | During procedure | C=30  I=30 | *Control*: median 4 (IQR 2-5) converted to 3.6435 (2.3353)  *Intervention*: median 4 (IQR 2-4) cannot convert due to skewed data  p=0.74 | | Unable to calculate Cohen’s d due to non-normal data distribution.  Finding: no significant difference in the dosage of sedative drugs between groups. |  |
| Huntington et al. (65) | Child Anxiety | FIS (child self-reported)  m-YPAS (observer-rated) | Recruitment into the study, on arrival in the day surgery unit, 48 hours and 1 week post-operatively  Before entry and inside anaesthesia induction room | C1=59  C2=55  I=60  C1=56  C2=55  I=55 | **Intervention group (preparatory game) relative to controls OR at recruitment**  *Control 1 (usual care):* 1.18 (2.57, 2.5) p=0.65  *Control 2 (handwashing game):* 1.25 (0.59, 2.7) p=0.56  **Intervention group (preparatory game) relative to controls OR on the ward**  *Control 1 (usual care):* 1.08 (0.56, 2.1) p=0.82  *Control 2 (handwashing game):* 0.90 (0.46, 1.8) p=0.77  **Intervention group (preparatory game) relative to controls OR 48 hours post procedure**  *Control 1 (usual care):* 1.31 (0.57, 3.0) p=0.52  *Control 2 (handwashing game):* 1.73 (0.73, 4.1) p=0.22  **Intervention group (preparatory game) relative to controls OR 1-week post procedure**  *Control 1 (usual care):* 0.54 (0.20, 1.49) p=0.23  *Control 2 (handwashing game):* 1.31 (0.39, 4.4) p=0.66  **The child observed anxiety pre-induction and at-induction**  *Control 1 (usual care):* 45.1 (20.5) range 22.9-100  *Control 2 (handwashing game):* 43.2 (20.7) range 22.9-95.8  *Intervention:* 47.6 (22.2) range 23-100  **Intervention group (preparatory game) relative to controls OR pre-induction**  *Control 1 (usual care):* 1.26 (0.61-2.6) p=0.53  *Control 2 (handwashing game):* 1.82 (0.87-3.81) p=0.11  **Intervention group (preparatory game) relative to controls OR at induction**  *Control 1 (usual care):* 1.02 (0.40-2.6) p=0.97  *Control 2 (handwashing game):* 1.38 (0.56, 3.4) p=0.49 | | Unable to calculate Cohen’s d due to the data presented.  Finding: the OR shows that anxiety is highest when children wait in the ward but otherwise anxiety is comparable between groups at all time points.  **The effect between Control 1 and Intervention pre- and at induction**  Cohen's d = (47.6 - 45.1) ⁄ 21.366914 = 0.117003  Glass's delta = (47.6 - 45.1) ⁄ 20.5 = 0.121951  Hedges' g = (47.6 - 45.1) ⁄ 21.35912 = 0.117046  **The effect between Control 2 and Intervention pre- and at induction**  Cohen's d = (47.6 - 43.2) ⁄ 21.463108 = 0.205003  Glass's delta = (47.6 - 43.2) ⁄ 20.7 = 0.21256  Hedges' g = (47.6 - 43.2) ⁄ 21.463108 = 0.205003  Finding: the same mean and standard deviations were recorded pre-induction and at induction. Overall, no significant difference in anxiety before and during induction between the three groups, although a small direction of effect was noted between the intervention and control groups.  Unable to calculate Cohen’s d due to data presented in the paper.  Finding: no significant difference in anxiety before and during induction between the three groups. |  |
|  | Induction behaviour | VAS (observer-rated and video recording) | During induction | C1=56  C2=55  I=55 | *Control 1 (usual care): 3.5* (2.5) range 0-9.5  *Control 2 (handwashing game):* 3.7 (2.4) range 0-10  *Intervention: 3.5* (2.6) range 0-10  **Intervention group (preparatory game) relative to controls OR pre-induction**  *Control 1 (usual care):* 2.0 (0.36-11.6) p=0.42  *Control 2 (handwashing game):* 1.53 (0.25,9.5) p=0.65  **Intervention group (preparatory game) relative to controls OR at induction**  *Control 1 (usual care):* 1.02 (0.40-2.6) p=0.97  *Control 2 (handwashing game):* 1.38 (0.56, 3.4) p=0.49 | | **The effect between Control 1 and Intervention**  Cohen's d = (3.5 - 3.5) ⁄ 2.55049 = 0  Glass's delta = (3.5 - 3.5) ⁄ 2.5 = 0  Hedges' g = (3.5 - 3.5) ⁄ 2.550031 = 0  **The effect between Control 2 and Intervention**  Cohen's d = (3.5 - 3.7) ⁄ 2.501999 = 0.079936  Glass's delta = (3.5 - 3.7) ⁄ 2.4 = 0.083333  Hedges' g = (3.5 - 3.7) ⁄ 2.501999 = 0.079936  Finding: no significant difference nor overall effect in behaviour between the three groups.  Unable to calculate Cohen’s d due to data presented in the paper.  Finding: no significant difference in induction behaviour between the three groups. |  |
|  | Parental satisfaction | VAS (observer asked questions)  TEI (observer asked questions) | In-person immediately before discharge from the ward  By telephone 48 hours after the procedure | ?  ? | **Satisfaction for VAS scores of 9 or 10 only across three areas:**  1. Hospital service (p=0.95):  *Control 1 (usual care):* 8.8 (1.2) range 6-10  *Control 2 (handwashing game)*: 8.8 (1.5) range 4-10  *Intervention*: 8.2 (2.5) range 0-10  2. Preparatory information (p=0.71):  *Control 1 (usual care):* 9.0 (1.0) range 5-10  *Control 2 (handwashing game)*: 8.9 (1.6) range 4-10  *Intervention:* 8.8 (1.9) range 0-10  3. Preparatory information assisting the child to prepare (p=0.63):  *Control 1 (usual care):* 9.0 (1.0) range 6-10  *Control 2 (handwashing game)*: 8.9 (1.4) range 3.5-10  *Intervention:* 8.8 (2.0) range 0-10  **Intervention group (preparatory game) relative to controls OR for the likelihood of ‘a lot/very satisfied’ for TEI1 “Do you think the information received helped your child to handle the visit better?”**  *Control 1 (usual care):* 12 (4.7-32) p<0.001  *Control 2 (handwashing game):* 8.2 (3-22) p<0.001  **Intervention group (preparatory game) relative to controls OR for the likelihood of ‘a lot/very satisfied’ for TEI2 “Do you think the information improved your child’s ability to cope?”**  *Control 1 (usual care):* 21 (8-56) p<0.001  *Control 2 (handwashing game):* 13 (5-34) p<0.001 | | Unable to calculate Cohen’s d due to lack of sample size information on those reporting VAS scores of 9 or 10.  Finding: every family reported high levels of satisfaction, but no significant differences were found between the three groups.  Unable to calculate Cohen’s d due to lack of sample size information.  Finding: significant difference between intervention and controls in terms of parental satisfaction for two questions scoring parents’ perception of how the preparation helped the child’s experience and improved the child’s ability to cope. No significant differences were reported for the other four questions. |  |
|  | Service throughput for anaesthetic induction | Measured in minutes | Data from routine NHS monitoring operating room management | C1=56  C2=55  I=55 | *Control 1 (usual care):* 9.2 (5.1) range 3-7  *Control 2 (handwashing game)*: 9.5 (4.3) range 0-21  *Intervention:* 9.33 (5.5) range 2-34 | | **The effect between Control 1 and Intervention**  Cohen's d = (9.33 - 9.2) ⁄ 5.303772 = 0.024511  Glass's delta = (9.33 - 9.2) ⁄ 5.1 = 0.02549  Hedges' g = (9.33 - 9.2) ⁄ 5.301938 = 0.024519  **The effect between Control 2 and Intervention**  Cohen's d = (9.33 - 9.5) ⁄ 4.936598 = 0.034437  Glass's delta = (9.33 - 9.5) ⁄ 4.3 = 0.039535  Hedges' g = (9.33 - 9.5) ⁄ 4.936598 = 0.034437  Finding: induction time comparable between the three groups with no overall effect observed. |  |
|  | Service throughput for time in recovery | Measured in minutes | Data from routine NHS monitoring operating room management | C1=59  C2=55  I=60 | *Control 1 (usual care):* mean 16.7 range 6-37 (95% CI 15,18.5)  *Control 2 (handwashing game)*: mean 15.3 range 6-34 (95% CI 13.8,17)  *Intervention:* mean 17.2 range 6-61 (95% CI 14.5,19.8) | | **RevMan Calculator (91) was used to determine Standard Error (0.892857) using 95% CI for control 1, then used to calculate Standard Deviation (4.869788) and effect**  Cohen's d = (17.2 - 16.7) ⁄ 4.869788 = 0.102674  Glass's delta = (17.2 - 16.7) ⁄ 4.869788 = 0.102674  Hedges' g = (17.2 - 16.7) ⁄ 4.869788 = 0.102674  **RevMan Calculator (91) was used to determine Standard Error (-0.81633) using 95% CI for control 2, then used to calculate Standard Deviation (-4.37294) and effect**  Cohen's d = (17.2 - 15.3) ⁄ 6.062269 = 0.313414  Glass's delta = (17.2 - 15.3) ⁄ 4.37294 = 0.43449  Hedges' g = (17.2 - 15.3) ⁄ 6.126265 = 0.31014  Finding: recovery time was comparable between the three groups with a slightly longer recovery time observed in the intervention group. |  |
|  | Service throughput for time on the ward after recovery | Measured in minutes | Data from routine NHS monitoring operating room management | C1=56  C2=55  I=55 | *Control 1 (usual care):* 55 (31) range 14-217  *Control 2 (handwashing game)*: 49 (22) range 20-120  *Intervention:* 47 (25) range 15-123 | | **The effect between Control 1 and Intervention**  Cohen's d = (47 - 55) ⁄ 28.160256 = 0.284088  Glass's delta = (47 - 55) ⁄ 31 = 0.258065  Hedges' g = (47 - 55) ⁄ 28.187609 = 0.283813  **The effect between Control 2 and Intervention**  Cohen's d = (47 - 49) ⁄ 23.547824 = 0.084934  Glass's delta = (47 - 49) ⁄ 22 = 0.090909  Hedges' g = (47 - 49) ⁄ 23.547824 = 0.084934  Finding: time of ward comparable between the three groups with a small direction of effect observed between control 2 (handwashing game) and intervention (video). |  |
| Fernandes et al. (67) | Child preoperative worries | CSWQ 5-point Likert scale from 0=not at all worried to 4=extremely worried across four domains  (self-reported) | After DHI before the procedure | C1=30  C2=30  I=30 | **Negative consequence**  *Control 1 (no intervention):* 1.91 (0.86)  *Control 2 (video game)*: 1.56 (0.73)  *Intervention:* 0.46 (0.18)  **Hospitalisation**  *Control 1 (no intervention):* 1.43 (0.86)  *Control 2 (video game)*: 1.24 (0.81)  *Intervention:* 0.37 (0.29)  **Medical procedure**  *Control 1 (no intervention):* 2.21 (1.09)  *Control 2 (video game)*: 1.82 (0.92)  *Intervention:* 0.44 (0.33)  **Illness**  *Control 1 (no intervention):* 2.08 (0.97)  *Control 2 (video game):* 1.63 (0.83)  *Intervention:* 0.58 (0.36) | | **The effect between Control 1 and Intervention for negative consequence**  Cohen's d = (0.46 - 1.91) ⁄ 0.621289 = 2.333858  Glass's delta = (0.46 - 1.91) ⁄ 0.86 = 1.686047  Hedges' g = (0.46 - 1.91) ⁄ 0.621289 = 2.333858  **The effect between Control 2 and Intervention for negative consequence**  Cohen's d = (0.46 - 1.56) ⁄ 0.531648 = 2.069037  Glass's delta = (0.46 - 1.56) ⁄ 0.73 = 1.506849  Hedges' g = (0.46 - 1.56) ⁄ 0.531648 = 2.069037  **The effect between Control 1 and Intervention for hospitalisation**  Cohen's d = (0.37 - 1.43) ⁄ 0.641755 = 1.65172  Glass's delta = (0.37 - 1.43) ⁄ 0.86 = 1.232558  Hedges' g = (0.37 - 1.43) ⁄ 0.641755 = 1.65172  **The effect between Control 2 and Intervention for hospitalisation**  Cohen's d = (0.37 - 1.24) ⁄ 0.608358 = 1.430078  Glass's delta = (0.37 - 1.24) ⁄ 0.81 = 1.074074  Hedges' g = (0.37 - 1.24) ⁄ 0.608358 = 1.430078  **The effect between Control 1 and Intervention for the medical procedure**  Cohen's d = (0.44 - 2.21) ⁄ 0.805295 = 2.197952  Glass's delta = (0.44 - 2.21) ⁄ 1.09 = 1.623853  Hedges' g = (0.44 - 2.21) ⁄ 0.805295 = 2.197952  **The effect between Control 2 and Intervention for the medical procedure**  Cohen's d = (0.44 - 1.82) ⁄ 0.691122 = 1.996752  Glass's delta = (0.44 - 1.82) ⁄ 0.92 = 1.5  Hedges' g = (0.44 - 1.82) ⁄ 0. 691122 = 1.996752  **The effect between Control 1 and Intervention for illness**  Cohen's d = (0.58 - 2.08) ⁄ 0.731608 = 2.050279  Glass's delta = (0.58 - 2.08) ⁄ 0.97 = 1.546392  Hedges' g = (0.58 - 2.08) ⁄ 0.731608 = 2.050279  **The effect between Control 2 and Intervention for illness**  Cohen's d = (0.58 - 1.63) ⁄ 0.639727 = 1.641326  Glass's delta = (0.58 - 1.63) ⁄ 0.83 = 1.26506  Hedges' g = (0.58 - 1.63) ⁄ 0.639727 = 1.641326  Finding: Across all four domains child self-reported worries were lower in the intervention group, showing a large negative direction of effect. Noted additionally that the means for negative consequences, medical procedures, and illness are lower in control 2 than in control 1. |  |
|  | Emotional response | SAM assesses feelings of arousal (clam/aroused) and valence (happy/unhappy) across five graphic figures (self-reported) | Before and after DHI and before Omron | C1=30  C2=30  I=30 | **Valence pre-DHI and before Omron**  *Control 1 (no intervention):* 4.03 (0.72)  *Control 2 (video game):* 4.00 (0.91)  *Intervention:* 4.17 (0.75)  **Valence post-DHI and before Omron**  *Control 1 (no intervention):* 4.30 (0.75)  *Control 2 (video game):* 4.57 (0.68)  *Intervention:* 4.47 (0.63)  **Arousal pre-DHI and before Omron**  *Control 1 (no intervention):* 2.37 (1.00)  *Control 2 (video game):* 2.57 (1.01)  *Intervention:* 2.17 (0.99)  **Arousal post-DHI and before Omron**  *Control 1 (no intervention):* 2.00 (0.87)  *Control 2 (video game):* 2.07 (0.83)  *Intervention:* 1.60 (0.62) | | **The effect between Control 1 and Intervention for valence pre-DHI and before Omron**  Cohen's d = (4.17 - 4.03) ⁄ 0.735153 = 0.190437  Glass's delta = (4.17 - 4.03) ⁄ 0.72 = 0.194444  Hedges' g = (4.17 - 4.03) ⁄ 0.735153 = 0.190437  **The effect between Control 2 and Intervention for valence pre-DHI and before Omron**  Cohen's d = (4.17 - 4) ⁄ 0.833847 = 0.203874  Glass's delta = (4.17 - 4) ⁄ 0.91 = 0.186813  Hedges' g = (4.17 - 4) ⁄ 0.833847 = 0.203874  **The effect between Control 1 and Intervention for valence post-DHI and before Omron**  Cohen's d = (4.47 - 4.3) ⁄ 0.676498 = 0.251294  Glass's delta = (4.47 - 4.3) ⁄ 0.72 = 0.236111  Hedges' g = (4.47 - 4.3) ⁄ 0.676498 = 0.251294  **The effect between Control 2 and Intervention for valence post-DHI and before Omron**  Cohen's d = (4.47 - 4.57) ⁄ 0.655477 = 0.152561  Glass's delta = (4.47 - 4.57) ⁄ 0.68 = 0.147059  Hedges' g = (4.47 - 4.57) ⁄ 0.655477 = 0.152561  Finding: no effect between intervention and control 1 pre-DHI but a small effect between DHI and control 2, with effect reversed for post-DHI. Overall, the results are comparable between the groups, and children generally felt happier after the intervention. The DHI did not lead to significantly higher emotions.  **The effect between Control 1 and Intervention for arousal pre-DHI and before Omron**  Cohen's d = (2.17 - 2.37) ⁄ 0.995013 = 0.201002  Glass's delta = (2.17 - 2.37) ⁄ 1 = 0.2  Hedges' g = (2.17 - 2.37) ⁄ 0.995013 = 0.201002  **The effect between Control 2 and Intervention for arousal pre-DHI and before Omron**  Cohen's d = (2.17 - 2.57) ⁄ 1.00005 = 0.3998  Glass's delta = (2.17 - 2.57) ⁄ 1.01 = 0.39604  Hedges' g = (2.17 - 2.57) ⁄ 1.00005 = 0.39998  **The effect between Control 1 and Intervention for arousal post-DHI and before Omron**  Cohen's d = (1.6 - 2) ⁄ 0.755414 = 0.529511  Glass's delta = (1.6 - 2) ⁄ 0.87 = 0.45977  Hedges' g = (1.6 - 2) ⁄ 0.755414 = 0.529511  **The effect between Control 2 and Intervention for arousal post-DHI and before Omron**  Cohen's d = (1.6 - 2.07) ⁄ 0.732564 = 0.641582  Glass's delta = (1.6 - 2.07) ⁄ 0.83 = 0.566265  Hedges' g = (1.6 - 2.07) ⁄ 0.732564 = 0.641582  Finding: a small effect was noted between intervention and both controls pre-DHI, with this increasing to a medium effect post-DHI. Overall, the results are comparable between the groups, but children generally felt calmer after the intervention. The DHI did not lead to significantly less arousal. |  |
|  | Physiological response | Omron R3 pulse monitor to collect heart rate & blood pressure stats | Before and after DHI and after SAM | C1=30  C2=30  I=30 | **Blood pressure pre-DHI and after SAM**  *Control 1 (no intervention):* 77.19 (11.95)  *Control 2 (video game):* 79.98 (11.64)  *Intervention:* 78.46 (11.25)  **Blood pressure post-DHI and after SAM**  *Control 1 (no intervention):* 76.39 (10.04)  *Control 2 (video game):* 76.94 (10.33)  *Intervention:* 75.01 (10.88)  **Heart rate pre-DHI and after SAM**  *Control 1 (no intervention):* 83.37 (18.29)  *Control 2 (video game):* 81.73 (16.74)  *Intervention:* 76.63 (10.68)    **Heart rate post-DHI and after SAM**  *Control 1 (no intervention):* 82.80 (17.66)  *Control 2 (video game):* 82.80 (13.90)  *Intervention:* 75.17 (9.90) | | **The effect between Control 1 and Intervention for blood pressure pre-DHI and after SAM**  Cohen's d = (78.46 - 77.19) ⁄ 11.605279 = 0.109433  Glass's delta = (78.46 - 77.19) ⁄ 11.95 = 0.106276  Hedges' g = (78.46 - 77.19) ⁄ 11.605279 = 0.109433  **The effect between Control 2 and Intervention for blood pressure pre-DHI and after SAM**  Cohen's d = (78.46 - 79.98) ⁄ 11.446661 = 0.13279  Glass's delta = (78.46 - 79.98) ⁄ 11.64 = 0.130584  Hedges' g = (78.46 - 79.98) ⁄ 11.446661 = 0.13279  **The effect between Control 1 and Intervention for blood pressure post-DHI and after SAM**  Cohen's d = (75.01 - 76.39) ⁄ 10.468429 = 0.131825  Glass's delta = (75.01 - 76.39) ⁄ 10.04 = 0.13745  Hedges' g = (75.01 - 76.39) ⁄ 10.468429 = 0.131825  **The effect between Control 2 and Intervention for blood pressure post-DHI and after SAM**  Cohen's d = (75.01 - 76.94) ⁄ 10.608565 = 0.181928  Glass's delta = (75.01 - 76.94) ⁄ 10.33 = 0.186834  Hedges' g = (75.01 - 76.94) ⁄ 10.608565 = 0.181928  Finding: Blood pressure results pre- and post-DHI and after SAM are comparable between the intervention and controls, and across all three groups.  **The effect between Control 1 and Intervention for heart rate pre-DHI and after SAM**  Cohen's d = (76.63 - 83.37) ⁄ 14.976423 = 0.450041  Glass's delta = (76.63 - 83.37) ⁄ 18.29 = 0.368507  Hedges' g = (76.63 - 83.37) ⁄ 14.976423 = 0.450041  **The effect between Control 2 and Intervention for heart rate pre-DHI and after SAM**  Cohen's d = (76.63 - 81.73) ⁄ 14.040833 = 0.363226  Glass's delta = (76.63 - 81.73) ⁄ 16.74 = 0.304659  Hedges' g = (76.63 - 81.73) ⁄ 14.040833 = 0.363226  **The effect between Control 1 and Intervention for heart rate post-DHI and after SAM**  Cohen's d = (75.17 - 82.8) ⁄ 14.315823 = 0.532977  Glass's delta = (75.17 - 82.8) ⁄ 17.66 = 0.43205  Hedges' g = (75.17 - 82.8) ⁄ 14.315823 = 0.532977  **The effect between Control 2 and Intervention for heart rate post-DHI and after SAM**  Cohen's d = (75.17 - 82.8) ⁄ 12.066897 = 0.632308  Glass's delta = (75.17 - 82.8) ⁄ 13.9 = 0.548921  Hedges' g = (75.17 - 82.8) ⁄ 12.066897 = 0.632308  Finding: Heart rate results pre-DHI and after SAM are comparable between controls 1 and 2 but show a small effect between the intervention and both controls. Similarly, heart rate post-DHI and after SAM is comparable between controls 1 and 2 but compared to the intervention shows a medium negative effect. Overall, the change in heart rate over time was not significant across all groups and participants. |  |
|  | Parental anxiety | STAI-Y 4-point scale on feeling from 1= not at all to 4=very much (observer asked questions) | After DHI | C1=30  C2=30  I=30 | *Control 1 (no intervention):* 2.19 (0.60)  *Control 2 (video game):* 1.85 (0.50)  *Intervention:* 1.89 (0.54) | | **The effect between Control 1 and Intervention for parental anxiety (p=0.033)**  Cohen's d = (1.89 - 2.19) ⁄ 0.570789 = 0.525588  Glass's delta = (1.89 - 2.19) ⁄ 0.6 = 0.5  Hedges' g = (1.89 - 2.19) ⁄ 0.570789 = 0.525588  **The effect between Control 2 and Intervention for parental anxiety (p=0.805)**  Cohen's d = (1.89 - 1.85) ⁄ 0.520384 = 0.076866  Glass's delta = (1.89 - 1.85) ⁄ 0.5 = 0.08  Hedges' g = (1.89 - 1.85) ⁄ 0.520384 = 0.076866  Finding: Parental anxiety was significantly lower in the intervention group compared to control group 1, with a negative medium effect. However, no difference (no effect) was observed between the intervention group and the control 2 group. |  |
|  | Child’s temperament | EAS-P (parental self-reported) | After DHI | ? | Results were not reported for temperament but rather data reported as correlations between the predictors and dependant variables. | | Unable to calculate Cohen’s d due to data presented in the paper. |  |
| Hatipoglu et. al (66) | Child preoperative anxiety | m-YPAS (observer-rated) | During induction | C1=33  C2=33  I=33 | *Control 1 (usual care):* 73.1 (18.0)  *Control 2 (voice recording):* 39.3 (19.2)  *Intervention:* 27.4 (7.1)  p<0.001 for Control 1 versus Control 2  p<0.001 for Control 1 versus Intervention  p<0.001 for Control 2 versus Intervention | | **The effect between Control 1 and Intervention**  Cohen's d = (27.4 - 73.1) ⁄ 13.682288 = 3.340085  Glass's delta = (27.4 - 73.1) ⁄ 18 = 2.538889  Hedges' g = (27.4 - 73.1) ⁄ 13.682288 = 3.340085  **The effect between Control 2 and Intervention**  Cohen's d = (27.4 - 39.3) ⁄ 14.474978 = 0.822108  Glass's delta = (27.4 - 39.3) ⁄ 19.2 = 0.619792  Hedges' g = (27.4 - 39.3) ⁄ 14.474978 = 0.822108  Finding: a large effect was observed between intervention and control groups 1 and 2, with preoperative anxiety of children significantly lower in the intervention group. |  |
|  | Behavioural changes | PHBQ (observer asked questions) | By telephone 7 days after hospital discharge | C1=33  C2=33  I=33  Unclear | *Control 1 (usual care):* 87.6 (3.4)  *Control 2 (voice recording):* 82.1 (1.8)  *Intervention:* 81.4 (2.6)  p<0.001 for Control 1 versus Control 2  p<0.001 for Control 1 versus Intervention  **Predictors of behavioural change as an OR with 95% CI**  *m-YPAS scores*: 1.03, 1.01-1.06 p=0.002  *Parental gender (female/male):* 4.05, 1.39-1.06 p=0.01  *Child age*: 0.40, 0.13-1.16 p=0.09 | | **The effect between Control 1 and Intervention**  Cohen's d = (81.4 - 87.6) ⁄ 3.026549 = 2.048538  Glass's delta = (81.4 - 87.6) ⁄ 3.4 = 1.823529  Hedges' g = (81.4 - 87.6) ⁄ 3.026549 = 2.048538  **The effect between Control 2 and Intervention**  Cohen's d = (81.4 - 82.1) ⁄ 2.236068 = 0.31305  Glass's delta = (81.4 - 82.1) ⁄ 1.8 = 0.388889  Hedges' g = (81.4 - 82.1) ⁄ 2.236068 = 0.31305  Finding: a large effect was observed between intervention and control group 1, with behavioural changes of children significantly better in both control 2 and intervention group compared to control 1. A small negative effect was observed between intervention and control group 2  Unable to calculate Cohen’s d due to no sample size information.  Finding: Anxious children (<30^th^ percentile) have a 1.03 times greater risk of adopting negative behaviours than calm children (>30^th^ percentile). Parental gender significantly correlates with behavioural change scores, with female parents having a 4.05 times greater risk of negative behaviours. No correlation between child age and behaviour  . |  |
| Eijlers et al. (68) | Child induction anxiety | m-YPAS (observer-rated) | During induction (T3) | C=97  I=94 | *Control*: median 38.3 (IQR 28.3-53.3) converted to 40.0601 (18.8136)  *Intervention*: median 40.0 (IQR 28.3-58.3) converted to 42.3237 (22.5872)  p=0.862 | | Cohen's d = (42.3237 - 40.0601) ⁄ 20.786211 = 0.108899  Glass's delta = (42.3237 - 40.0601) ⁄ 18.8136 = 0.120317  Hedges' g = (42.3237 - 40.0601) ⁄ 20.756364 = 0.109056  Finding: no effect was observed with results comparable between the two groups. |  |
|  | Child self-reported anxiety | m-YPAS (observer-rated)  VAS (self-reported) | At hospital admission (T1) and holding area before the procedure (T2)  At hospital admission (T1), in the holding area before the procedure (T2), in the recovery room (T4) and at home post procedure (T5) | C=97  I=94 | **m-YPAS at admission (T1)**  *Control*: median 26.7 (IQR 23.3-32.5) converted to 27.5448 (6.9234)  *Intervention*: median 28.3 (IQR 23.3-31.7) converted to 27.7367 (6.3244)  p=0.697  **m-YPAS in holding area (T2)**  *Control*: median 28.3 (IQR 23.3-41.7) cannot convert due to skewed data  *Intervention*: median 28.3 (IQR 23.3-36.7) converted to 29.4971 (10.089)  p=0.765  **VAS at admission (T1)**  *Control*: median 1.5 (IQR 0.0-5.0) cannot convert due to skewed data  *Intervention*: median 3.0 (IQR 0.1-5.0) converted to 2.6831 (3.6892)  p=0.407  **VAS in holding area (T2)**  *Control*: median 3.5 (IQR 0.0-6.0) converted to 3.148 (4.5153)  *Intervention*: median 3.0 (IQR 1.0-5.5) converted to 3.176 (3.3881)  p=0.753  **VAS in recovery (T4)**  *Control*: median 0.0 (IQR 0.0-2.0) cannot convert due to skewed data  *Intervention*: median 0.0 (IQR 0.0-2.0) cannot convert due to skewed data  p=0.735  **VAS at home (T5)**  *Control*: median 0.3 (IQR 0.0-2.0) cannot convert due to skewed data  *Intervention*: median 0.5 (IQR 0.0-1.0) converted to 0.5 (0.7529)  p=0.727 | | **The effect between Control and Intervention for m-YPAS at admission (T1)**  Cohen's d = (27.7367 - 27.5448) ⁄ 6.630667 = 0.028941  Glass's delta = (27.7367 - 27.5448) ⁄ 6.9234 = 0.027718  Hedges' g = (27.7367 - 27.5448) ⁄ 6.635415 = 0.028921  Finding: no effect was observed with results comparable between the two groups.  Unable to calculate Cohen’s d due to non-normal data distribution.  Finding: no effect was observed with results comparable between the two groups.  Unable to calculate Cohen’s d due to non-normal data distribution.  Finding: no effect was observed with results comparable between the two groups.  **The effect between Control and Intervention for VAS in holding area (T2)**  Cohen's d = (3.176 - 3.148) ⁄ 3.991689 = 0.007015  Glass's delta = (3.176 - 3.148) ⁄ 4.5153 = 0.006201  Hedges' g = (3.176 - 3.148) ⁄ 4.000535 = 0.006999  Finding: no effect was observed with results comparable between the two groups.  Unable to calculate Cohen’s d due to non-normal data distribution.  Finding: no effect was observed with results comparable between the two groups.  Unable to calculate Cohen’s d due to non-normal data distribution.  Finding: no effect was observed with results comparable between the two groups. |  |
|  | Child behaviour | CBCL (by parent) | At hospital admission (T1) | C=97  I=94 | *Control*: median 46.0 (IQR 39.0-53.0) converted to 46 (10.5356)  *Intervention*: median 47.0 (IQR 41.0-56.0) converted to 48.0562 (11.2936)  p=0.251 | | **The effect between Control and Intervention for CBCL in the holding area (T1)**  Cohen's d = (48.0562 - 46) ⁄ 10.921178 = 0.188276  Glass's delta = (48.0562 - 46) ⁄ 10.5356 = 0.195167  Hedges' g = (48.0562 - 46) ⁄ 10.915164 = 0.18838  Finding: no effect was observed with results comparable between the two groups. |  |
|  | Self-reported and observed pain | FPS-r (child self-reported)  PPPM (parent-reported)  FLACC (observer-rated) | In recovery room (T4) and at home (T5)  At home (T5)  In the recovery room (T4) | C=97  I=94 | **FPS-r at recovery (T4)**  *Control*: median 2.0 (IQR 0.0-2.5) cannot convert due to skewed data  *Intervention*: median 2.0 (IQR 0.0-4.0) converted to 2 (3.0116)  p=0.699  **FPS-r at recovery (T4)**  *Control*: median 0.0 (IQR 0.0-2.0) cannot convert due to skewed data  *Intervention*: median 0.0 (IQR 0.0-2.0) cannot convert due to skewed data  p=0.454  **PPPM at home (T5)**  *Control*: median 3.0 (IQR 1.0-8.0) cannot convert due to skewed data  *Intervention*: median 3.0 (IQR 0.0-5.0) converted to 2.6479 (3.7645)  p=0.410  **FLACC in recovery (T4)**  *Control*: median 0.0 (IQR 0.0-2.0) cannot convert due to skewed data  *Intervention*: median 0.0 (IQR 0.0-2.0) cannot convert due to skewed data  p=0.454 | | Unable to calculate Cohen’s d due to non-normal data distribution.  Finding: no effect was observed with results comparable between the two groups.  Unable to calculate Cohen’s d due to non-normal data distribution.  Finding: no effect was observed with results comparable between the two groups.  Unable to calculate Cohen’s d due to non-normal data distribution.  Finding: no effect was observed with results comparable between the two groups.  Unable to calculate Cohen’s d due to non-normal data distribution.  Finding: no effect was observed with results comparable between the two groups. |  |
|  | ED | PAED (observer-rated) | In the recovery room (T4) | C=97  I=94 | *Control*: median 6.0 (IQR 5.0-9.0) cannot convert due to skewed data  *Intervention*: median 7.5 (IQR 5.0-9.0) converted to 7.1479 (3.0116)  p=0.266 | | Unable to calculate Cohen’s d due to non-normal data distribution.  Finding: no effect was observed with results comparable between the two groups. |  |
|  | Need for rescue analgesia | Administration of morphine | In the recovery room (T4) | C=97  I=94 | **Overall:**  *Control*: frequency 39 (40.2%)  *Intervention*: frequency 28 (29.8%)  p=0.131  **Adenoidectomy and tonsillectomy**:  *Control*: frequency 22 (95.7%)  *Intervention*: frequency 11 (55%)  p=0.002 | | **Chi-square p-value calculation (85):**  Cohen’s d = 0.2199  95% C.I = -0.0655 (0.5052)  v = 0.0212  **Chi-square p-value calculation (85):**  Cohen’s d = 0.4588  95% C.I = 0.1678 (0.7498)  v = 0.022  Finding: no significant difference but a small negative effect in the intervention compared to control, with less overall need for rescue analgesia. A significant difference between groups for adenoidectomy and tonsillectomy was found. |  |
|  | Parental anxiety | STAI (parent self-reported)  VAS (observer-rated) | Immediately after child induction (T3)  Immediately after child induction (T3) | C=97  I=94 | **STAI at induction (T3)**  *Control*: median 40.5 (IQR 33.0-50.0) converted to 41.204 (12.7933)  *Intervention*: median 41.0 (IQR 34.5-48.5) converted to 41.3521 (10.5407)  p=0.753  **VAS at induction (T3)**  *Control*: median 3.5 (IQR 2.0-5.0) converted to 3.5 (2.2576)  *Intervention*: median 3.0 (IQR 2.0-5.0) cannot convert due to skewed data  p=0.418 | | **The effect between Control and Intervention for STAI at induction (T3)**  Cohen's d = (41.3521 - 41.204) ⁄ 11.721239 = 0.012635  Glass's delta = (41.3521 - 41.204) ⁄ 12.7933 = 0.011576  Hedges' g = (41.3521 - 41.204) ⁄ 11.73902 = 0.012616  Finding: no effect was observed with results comparable between the two groups.  Unable to calculate Cohen’s d due to non-normal data distribution.  Finding: no effect was observed with results comparable between the two groups. |  |
| Liguori et al. (69) | Child preoperative anxiety | m-YPAS (observer-rated) | The afternoon before the procedure immediately before DHI (T1) and immediately on entering the operating room (T2) | C=20  I=20 | **m-YPAS before the procedure and DHI (T1)**  *Control*: 37.1 (13.8)  *Intervention*: 37.3 (21.7)  **m-YPAS on entering the operating room (T2)**  *Control*: 48.6 (15.9)  *Intervention*: 33.0 (18.4)  p=0.009  **m-YPAS mean the difference between the first and second measurement**  *Control:* 10.7 (2.8)  *Intervention*: -2.8 (7.2)  p=0.003 | | **The effect between Control and Intervention m-YPAS before the procedure and DHI (T1)**  Cohen's d = (37.3 - 37.1) ⁄ 18.184196 = 0.010999  Glass's delta = (37.3 - 37.1) ⁄ 13.8 = 0.014493  Hedges' g = (37.3 - 37.1) ⁄ 18.184196 = 0.010999  Finding: no effect was observed with anxiety comparable between the two groups before the intervention.  **The effect between Control and Intervention for m-YPAS holding area (T2)**  Cohen's d = (33 - 48.6) ⁄ 17.195494 = 0.907214  Glass's delta = (33 - 48.6) ⁄ 15.9 = 0.981132  Hedges' g = (33 - 48.6) ⁄ 17.195494 = 0.907214  Finding: a large effect was observed, with anxiety significantly lower in the intervention group.  **The effect between Control and Intervention f*or VAS in holding area (T2)***  Cohen's d = (-2.8 - 10.7) ⁄ 5.4626 = 2.471351  Glass's delta = (-2.8 - 10.7) ⁄ 2.8 = 4.821429  Hedges' g = (-2.8 - 10.7) ⁄ 5.4626 = 2.471351  Finding: a large effect was observed, with average anxiety between time points significantly different. |  |
| Ryu et al. (70) | Child preoperative anxiety | Korean version of m-YPAS (observer-rated) | At admission before DHI (T1) and just before transportation to the operating room (T2) | C=35  I=34 | **m-YPAS at admission (T1)**  *Control:* median 50.0 (IQR 43.3-65) converted to 52.9512 (16.7737)  *Intervention:* median 51.7 (IQR 46.7-67.5) cannot convert due to skewed data  p=0.389  **m-YPAS before transportation (T2)**  *Control:* median 46.7 (IQR 31.7-51.7) cannot convert due to skewed data  *Intervention:* median 28.3 (IQR 23.3-36.7) converted to 29.5095 (10.3707)  p<0.001  **m-YPAS mean the difference between the first and second measurement**  *Control:* median 0 (IQR -20-4.2) cannot convert due to skewed data  *Intervention:* median -22.5 (IQR -29.6- -14.2) converted to -22.0731 (11.9186)  p=0.002 | | Unable to calculate Cohen’s d due to non-normal data distribution.  Finding: anxiety at baseline was comparable between the two groups.  Unable to calculate Cohen’s d due to non-normal data distribution.  Finding: anxiety pre-anaesthesia (on transportation to the operating room) was significantly lower in the intervention group.  Unable to calculate Cohen’s d due to non-normal data distribution.  Finding: anxiety at admission before the DHI and on transportation to the operating room was significantly lower in the intervention group. |  |
|  | Induction compliance | ICC (observer-rated) | During induction (T3) | C=35  I=34 | *Control ICC score:*  -perfect: n=19 (54%)  -moderate: n=13 (37%)  -poor: n=3 (9%)  *Intervention ICC score:*  -perfect: n=27 (79%)  -moderate: n=7 (21%)  -poor: n=0 (0%)  p=0.038 | | Cohen’s d =0.5159  95% C.I =0.0286 , 1.0033  v = 0.0618  Finding: children in the intervention group were more compliant during induction than children in the control group. |  |
|  | Procedural behaviour | PBRS (observer-rated) | During induction (T3) | C=35  I=34 | *Control:* median 1 (IQR 0-2) converted 1 (1.546)  *Intervention:* median 0 (IQR 0-1) cannot convert due to skewed data  p=0.92 | | Unable to calculate Cohen’s d due to non-normal data distribution.  Finding: procedural behaviour comparable between the two groups. |  |
|  | Parental satisfaction | NRS where 0 = very dissatisfied and 100 = very satisfied (self-reported) | After the parent is guided to the waiting room (T4) | C=35  I=34 | *Control:* median 100 (IQR 90-100) cannot convert due to skewed data  *Intervention:* median 100 (IQR 90-100) cannot convert due to skewed data  p=0.268 | | Unable to calculate Cohen’s d due to non-normal data distribution.  Finding: parental satisfaction is comparable between the two groups. |  |
| Fortier et al. (71) | Child preoperative anxiety | m-YPAS (observer-rated) | Parental separation to the operating room (T1b), entrance to the operating room (T2) and during induction (T3) | C=41  I=33 | **m-YPAS at separation (T1b)**  *Control:* 40.7 (16.6)  *Intervention:* 36.4 (12.7)  p=0.23  **m-YPAS at the entrance to operating room (T2)**  *Control:* 46.0 (19.0)  *Intervention:* 36.2 (14.1)  p=0.02  **m-YPAS at induction (T3)**  *Control:* 57.0 (21.2)  *Intervention:* 43.5 (21.7)  p=0.01 | | Cohen’s d = 0.29  Finding: child anxiety is comparable between groups at separation, with an overall small effect.  Cohen’s d = 0.59  Finding: child anxiety was significantly lower in the intervention group at the entrance to operating room, with an overall medium effect.  Cohen’s d = 0.63  Finding: child anxiety was significantly lower in the intervention group at induction, with an overall medium effect. |  |
|  | Parental preoperative anxiety | STAI (self-reported) | In the preoperative holding area (T1a) and upon separation to go to the operating room (T1b) | C=42  I=38 | **STAI in holding area (T1a)**  *Control:* 36.8 (7.1)  *Intervention:* 32.7 (7.9)  p=0.004  **STAI at separation (T1b)**  *Control:* 47.2 (12.8)  *Intervention:* 43.8 (13.4)  p=0.06 | | Cohen’s d = 0.65  Finding: parental anxiety was significantly lower in the intervention group in the holding area, with an overall medium effect.  Cohen’s d = 0.25  Finding: parental anxiety is comparable between groups at separation, with an overall small effect. |  |
|  | ED | PAED (observer-rated) | ? (Unclear but potential from medical records) | C=44  I=38 | *Control:* 13.6 (2.9)  *Intervention:* 12.3 (2.9)  p=0.04 | | Cohen's d = (12.3 - 13.6) ⁄ 2.9 = 0.448276  Glass's delta = (12.3 - 13.6) ⁄ 2.9 = 0.448276  Hedges' g = (12.3 - 13.6) ⁄ 2.9 = 0.448276  Finding: ED was significantly lower in the intervention group compared to the control with a small negative effect. |  |
|  | Intraoperative Analgesic consumption | Measured in mg/kg | From medical records post procedure | Unclear | Morphine (mg/kg):  *Control:* 0.17 (0.36)  *Intervention*: 0.09 (0.01)  p= 0.741  Meperidine (mg/kg)  *Control:* 0.74 (0.32)  *Intervention:* 0.76 (0.30)  p=0.825  Fentanyl (mcg/kg)  *Control:* 1.48 (0.89)  *Intervention:* 1.40 (0.47)  p=0.856  Acetaminophen (mg/kg)  *Control:* 29.34 (5.10)  *Intervention:* 26.62 (2.26)  p=0.204 | | Unable to calculate Cohen’s d due to lack of information on sample size for each measure.  Finding: no significant difference in analgesics use between the two groups. |  |
|  | Pain ratings | NRS 0-10 scale (nurse rated) | From medical records post procedure | C=44  I=38 | *Control:* median 0.38 (IQR 1.76)  *Intervention*: median 0 (IQR 0.67)  p=0.30 | | Unable to calculate Cohen’s d due to IQR not being provided as a range.  Finding: pain rating comparable between the groups. |  |
|  | Length of surgery | Measured in minutes | From medical records post procedure | C=44  I=38 | *Control:* 33 (18)  *Intervention*: 35 (IQR 22)  p=0.708 | | Unable to calculate Cohen’s d due to IQR not being provided as a range.  Finding: no effect between the groups, with the length of surgery generally comparable. |  |
|  | Length in PACU | Measured in minutes | From medical records post procedure | C=44  I=38 | *Control:* median 88 (IQR 54)  *Intervention*: median 71 (IQR 68)  p=0.26 | | Unable to calculate Cohen’s d due to IQR not being provided as a range.  Finding: pain rating comparable between the groups. |  |
| Campbell et al. (72) | Child dental anxiety levels | MCDAS (parent-reported) | At the time of recruitment into the study (T1) | C1=66  C2=63  I=63 | *Control 1 (usual care):* phobic n=58 (26%)  *Control 2 (cartoon):* phobic n=45 (29%)  *Intervention:* phobic n= 59 (22%)  P=0.720 | | Unable to calculate Cohen’s d based on data.  Finding: parents reported higher anxiety in children which contrasts with the child's self-reported anxiety which was much lower. |  |
|  | Preoperative anxiety | VAS (child self-reported)  VAS (observer-rated) | At the time of recruitment into the study (T1)  During induction (T2) and recovery (T3) | C1=66  C2=63  I=63  C1=58  C2=55  I=55  C1=56  C2=55  I=55 | **VAS at time of recruitment (T1)**  *Control 1 (usual care):* median 2 (IQR 0-10) cannot convert due to skewed data  *Control 2 (cartoon):* median 1 (IQR 0-10) cannot convert due to skewed data  *Intervention:* median 1 (IQR 0-10) cannot convert due to skewed data  p=0.790  **VAS at induction (T2)**  *Control 1 (usual care):* median 3 (IQR 0-10) cannot convert due to skewed data  *Control 2 (cartoon):* median 1 (IQR 0-10) cannot convert due to skewed data  *Intervention:* median 1 (IQR 0-10) cannot convert due to skewed data  Intervention versus control 1: p=0.014  Intervention versus control 2: p=0.798  Control 1 versus control 2: p=0.076  **VAS at recovery (T3)**  *Control 1 (usual care):* median 2.5 (IQR 0-10) cannot convert due to skewed data  *Control 2 (cartoon):* median 4 (IQR 0-10) converted to 4.7071 (7.6127)  *Intervention:* median 0 (IQR 0-10) cannot convert due to skewed data  Intervention versus control 1: p=0.121  Intervention versus control 2: p=0.016  Control 1 versus control 2: p=0.36 | | Unable to calculate Cohen’s d due to non-normal data distribution.  Finding: preoperative anxiety was comparable across three groups at the time of recruitment with no statistically significant difference observed.  Unable to calculate Cohen’s d due to non-normal data distribution.  Finding: preoperative anxiety comparable between intervention and control 2 at induction with no statistically significant difference observed. However, the intervention group had statistically significant lower preoperative anxiety than control 1 but not between the two controls.  Unable to calculate Cohen’s d due to non-normal data distribution.  Finding: preoperative anxiety at recovery was lower in the intervention group compared to controls. The intervention group had statistically significant lower preoperative anxiety than the control 2. |  |
| Wakimizu et al. (73) | Child anxiety | Wong-Baker FACES Rating Scale | Before DHI, twice perioperatively, once at discharge and three times post discharge with the last being 1-month post procedure | C=71  I=73 | **Baseline:**  *Control:* 1.12 (1.33)  *Intervention:* 1.31 (1.52)  **Preoperative**  *Control:* 2.06 (1.89)*  *Intervention:* 1.30 (1.42)*  **Postoperative**  *Control:* 2.13 (1.81)  *Intervention:* 2.03 (SD 1.77)  **Discharge**  *Control:* 0.79 (1.10)  *Intervention:* 0.73 (1.05)  **3 days after**  *Control:* 0.61 (0.98)  *Intervention:* 0.50 (0.83)  **1 week after**  *Control:* 0.45 (1.04)  *Intervention:* 0.30 (0.66)  **1 month after**  *Control:* 0.33 (0.84)  *Intervention:* 0.15 (0.46)  p=0.048 for group and p=0.038 group x time | | **The effect between Control and Intervention at baseline**  Cohen's d = (1.31 - 1.12) ⁄ 1.428163 = 0.133038  Glass's delta = (1.31 - 1.12) ⁄ 1.33 = 0.142857  Hedges' g = (1.31 - 1.12) ⁄ 1.429498 = 0.132914  **The effect between Control and Intervention preoperatively**  Cohen's d = (1.3 - 2.06) ⁄ 1.671601 = 0.454654  Glass's delta = (1.3 - 2.06) ⁄ 1.89 = 0.402116  Hedges' g = (1.3 - 2.06) ⁄ 1.668321 = 0.455548  **The effect between Control and Intervention postoperatively**  Cohen's d = (2.03 - 2.13) ⁄ 1.790112 = 0.055862  Glass's delta = (2.03 - 2.13) ⁄ 1.81 = 0.055249  Hedges' g = (2.03 - 2.13) ⁄ 1.78983 = 0.055871  **The effect between Control and Intervention at discharge**  Cohen's d = (0.73 - 0.79) ⁄ 1.075291 = 0.055799  Glass's delta = (0.73 - 0.79) ⁄ 1.1 = 0.054545  Hedges' g = (0.73 - 0.79) ⁄ 1.074939 = 0.055817  **The effect between Control and Intervention 3 days after**  Cohen's d = (0.5 - 0.61) ⁄ 0.908102 = 0.121132  Glass's delta = (0.5 - 0.61) ⁄ 0.98 = 0.112245  Hedges' g = (0.5 - 0.61) ⁄ 0.907049 = 0.121272  **The effect between Control and Intervention 1 week after**  Cohen's d = (0.3 - 0.45) ⁄ 0.870976 = 0.17222  Glass's delta = (0.3 - 0.45) ⁄ 1.04 = 0.144231  Hedges' g = (0.3 - 0.45) ⁄ 0.868361 = 0.172739  **The effect between Control and Intervention 1 month after**  Cohen's d = (0.15 - 0.33) ⁄ 0.6772 = 0.2658  Glass's delta = (0.15 - 0.33) ⁄ 0.84 = 0.214286  Hedges' g = (0.15 - 0.33) ⁄ 0.674627 = 0.266814  Finding: child anxiety comparable between groups at baseline, postoperatively, at discharge and 2 days after the procedure with no overall effect noted. Child anxiety was significantly lower preoperatively, with a small negative effect between the groups. Similarly, child anxiety was lower 1 week after and 1 month after in the intervention group with an overall small negative effect between the groups. Over time, anxiety was significantly lower with p=0.038. Scores were always lower in the intervention group compared to the control group. |  |
|  | Caregiver anxiety | STAI | 1 week before the procedure, preoperative period, postoperative period on the day of the procedure, 1 week after the procedure and 1 month after the procedure |  | **Baseline:**  *Control:* 45.36 (7.94)  *Intervention:* 44.10 (7.66)  **Preoperative**  *Control:* 48.16 (8.99)  *Intervention:* 46.58 (8.15)  **Postoperative**  *Control:* 42.11 (7.54)*  *Intervention:* 37.55 (7.54)*  **1 week after**  *Control:* 33.99 (7.21)  *Intervention:* 32.15 (8.42)  **1 month after**  *Control:* 30.12 (7.87)  *Intervention:* 30.03 (7.80)    p=0.017 for group and p=0.101 group x time | | **The effect between Control and Intervention at baseline**  Cohen's d = (44.1 - 45.36) ⁄ 7.801256 = 0.161512  Glass's delta = (44.1 - 45.36) ⁄ 7.94 = 0.15869  Hedges' g = (44.1 - 45.36) ⁄ 7.799285 = 0.161553  **The effect between Control and Intervention preoperatively**  Cohen's d = (46.58 - 48.16) ⁄ 8.580286 = 0.184143  Glass's delta = (46.58 - 48.16) ⁄ 8.99 = 0.175751  Hedges' g = (46.58 - 48.16) ⁄ 8.574375 = 0.18427  **The effect between Control and Intervention postoperatively**  Cohen's d = (37.55 - 42.11) ⁄ 7.54 = 0.604775  Glass's delta = (37.55 - 42.11) ⁄ 7.54 = 0.604775  Hedges' g = (37.55 - 42.11) ⁄ 7.54 = 0.604775  **The effect between Control and Intervention 1 week after**  Cohen's d = (32.15 - 33.99) ⁄ 7.838383 = 0.234742  Glass's delta = (32.15 - 33.99) ⁄ 7.21 = 0.255201  Hedges' g = (32.15 - 33.99) ⁄ 7.846874 = 0.234488  **The effect between Control and Intervention 1 month after**  Cohen's d = (30.03 - 30.12) ⁄ 7.835078 = 0.011487  Glass's delta = (30.03 - 30.12) ⁄ 7.87 = 0.011436  Hedges' g = (30.03 - 30.12) ⁄ 7.834585 = 0.011488  Finding: parental anxiety comparable between groups at baseline, preoperatively and 1 week after the procedure, with a small negative effect noted at 1 week. Parental anxiety was significantly lower postoperatively, with a medium negative effect between the groups. Parental anxiety at 1 month was comparable between the two groups Over time, parental anxiety had no significant difference p=0.101, but did show a significant difference between groups at p=0.017. Scores were always lower in the intervention group compared to the control group. |  |
|  | Degree of knowledge & information given | 4-point scale from ‘not explained at all’ to ‘explained sufficiently’ across 5 domains | Post DHI use | C=76  I=74 | Reason for undergoing surgery  Group effects: Z=-2.843, p= 0.004  The outline of surgery and hospitalisation  Group effects: Z=-0.059, p= 0.953  Anaesthesia induction  Group effects: Z=-2.19, p= 0.029  Post-surgery pains and daily life  Group effects: Z=-1.775, p= 0.076  Caregivers rooming-in  Group effects: Z=-0.997, p= 0.319 | | Unable to calculate Cohen’s d due to data reported  Finding: parents or caregivers provided more information in the intervention group compared to the control group. Of the 5 domains, a statistically significant difference was observed in ‘reason for undergoing surgery’ and ‘anaesthesia induction’ in terms of information provided by parents or caregivers in the intervention to control groups, with no statistically significant difference in the other domains. |  |
|  | Parental satisfaction | 4-point scale | Post DHI | I=72 | n=66 (91.7%) parents or caregivers in the intervention group expressed satisfaction with the DHI.  n=6 (8.3%) parents or caregivers in the intervention group expressed less or no satisfaction with the DHI. | | Unable to calculate Cohen’s d due to the data reported.  Finding: most parents or caregivers in the intervention group were satisfied with the DHI. |  |
| Dehghan et al. (74) | Child preoperative anxiety | Standardised YPAS | Before intervention in one DHI group and one control pre-test group, after intervention in all four groups | Unclear | **YPAS at baseline in intervention group 1 with pre-test (E1) and control group 1 with pre-test (C1) using Mann-Whitney test:**  *Activity E1 & C1:* p=0.143  *Vocalization E1 & C1:* p=0.019  *Emotional expressivity E1 & C1*: p=0.393  *State of apparent arousal E1 & C1*: p=0.912  **YPAS post-intervention scores in each domain before (b) and after (a) the intervention in intervention group 1 with pre-test (E1) and control group 1 with pre-test (C1) using the Wilcoxon test:**  *Activity E1b & E1a:* p=0.026  *Vocalization E1b & E1a:* p=0.007  *Emotional expressivity E1b & E1a*: p=0.015  *State of apparent arousal E1b & E1a*: p=0.063  *Activity C1b & C1a:* p=0.334  *Vocalization C1b & C1a:* p=0.054  *Emotional expressivity C1b & C1a*: p=0.023  *State of apparent arousal C1b & C1a*: p=1 | | Unable to calculate Cohen’s d lack of data reported.  Finding: a significant difference was found only in the vocalisation domain between E1 and C1.  Unable to calculate Cohen’s d lack of data reported.  Finding: a significant difference was found in all domains except arousal for intervention with the pre-test group (E1) before and after the intervention. A significant difference was found in only the emotional expressivity domain for the control with the pre-test group before and after the intervention.  Overall findings: the DHI was used for distraction, not preparation, and the quality of the study was considered poor. The results are unclear although the study authors suggest there was reduced anxiety in the intervention groups compared to the control group. |  |
| **Abbreviations used in table**  A-VAS - Anxiety Visual Analogue Scale  C - Control group  CBCL - Child Behaviour Checklist  CLP - Child Life Program  CSWQ - Child Surgery Worries Questionnaire  EAS-P - Emotionality Activity Sociability Temperament Survey for Children: Parental Ratings  ED - Emergence Delirium  FIS - Facial Image Scale  FLACC - Face, Legs, Activity, Cry and Consolability  FPS-r - Faces Pain Scale (self-reported)  I - Intervention group – the tested DHI  ICC - Induction Compliance Checklist  MCDAS - Modified Child Dental Anxiety Scale  m-YPAS - Modified Yale Preoperative Anxiety Scale  NRS - Numerical Rating Scale | | | | | | P - p-value  PACU - Post-Anaesthesia Care Unit  PAED - Paediatric Anaesthesia Emergence Delirium  PBRS - Procedural Behaviour Rating Score  PHBQ - Post-Hospitalisation Behaviour Questionnaire  PHBQ-AS - Post-Hospitalisation Behaviour Questionnaire for Ambulatory Surgery  PPPM - Parents Postoperative Pain Measure  SAM - Self-Assessment Manikin  SMP - Standard Preparatory Manual  STAI - State-Trait Anxiety Inventory  STAI-Y - State-Trait Anxiety Inventory Form Y  TEI - Treatment Evaluation Inventory  USE - Usefulness, Satisfaction and Ease of Use Questionnaire  VAS - Visual Analogue Scale  YPAS - Yale Preoperative Anxiety Scale  Z - Z statistic in Mann-Whitney U-test | | |
